# Chemical Upcycling of Expired Pharmaceuticals as a Source of Value-Added Chemicals for Organic Synthesis and Medicinal Chemistry

**DOI:** 10.3390/molecules29204811

**Published:** 2024-10-11

**Authors:** Teresa Abad-Grillo, Grant McNaughton-Smith

**Affiliations:** 1 Departamento de Química Orgánica, Universidad de La Laguna, Avenida Astrofísico Francisco Sánchez, 2, 38206 La Laguna, Tenerife, Spain; 2 Centro Atlántico del Medicamento S.A. (CEAMED S.A.), PCTT, 38200 La Laguna, Tenerife, Spain; gmcsmith@ceamedsa.com

**Keywords:** expired pharmaceuticals, extraction of active ingredients, chemical upcycling, azaspiro compounds, potentially bioactive compounds

## Abstract

Pharmaceutical and veterinary products are a class of contaminants of emerging concern, and their presence in the environment is due to continuous and incorrect disposal. Environmental scientists have been accumulating data on their adverse effects on animal populations since toxicological effects on wildlife were first published. Therefore, recycling strategies are needed. Valuable active ingredients can be extracted from expired pharmaceuticals and recycled according to various strategies. In an effort to reveal the potential of the chemical upcycling of expired pharmaceuticals, the active ingredients gabapentin and pregabalin were extracted and used as starting materials to prepare a small collection of promising substrates endowed with functionalities and structural three-dimensionality. Gabapentin **1** was transformed into aminoalcohol **3**, spiroamine **4,** and the bioactive azaspirolactam **5**. The lactam analog **6** was synthesized from pregabalin **2**. Due to the biological profile of **5** and the structural similarity of the *N*-alkylated derivatives **5l** and **6b** with the drug piracetam, a collection of potentially bioactive structural analogs **5a-l** and **6a-b** were also prepared. Simple extraction, synthesis, and purification procedures were used as a means of chemical and economic revaluation, resulting in moderate to good yields at a low cost.

## 1. Introduction

Major improvements in analytical science at the end of the twentieth century aroused special concern regarding the presence of pharmaceuticals in the environment and its consequences. Their presence in the environment is caused by continual discharges from households, animal farms, industry, waste-water treatment plant (WWTP), and hospitals [1,2]. The toxicological effects on wildlife caused by pharmaceutical residues at environmentally relevant concentrations have been reported [3,4,5]. The threats to wildlife are real and there is growing concern about human exposure, including the long-term effects, mixing of compounds, and effects at different ages [6,7,8].

A recycling strategy is needed. The recycling of pharmaceuticals, which are essentially chemical waste, can be achieved through the distribution of unopened and non-expiring medicines to patients in need of the medicines after quality checking. On the other hand, valuable active ingredients can be extracted from expired pharmaceuticals and recycled using various strategies. They can then be used in education and research at universities and schools [9,10] and as useful reagents for organic synthesis and industrial production [11,12,13,14,15,16,17] or reformulated into new drug products [18,19].

**Scheme 1:**
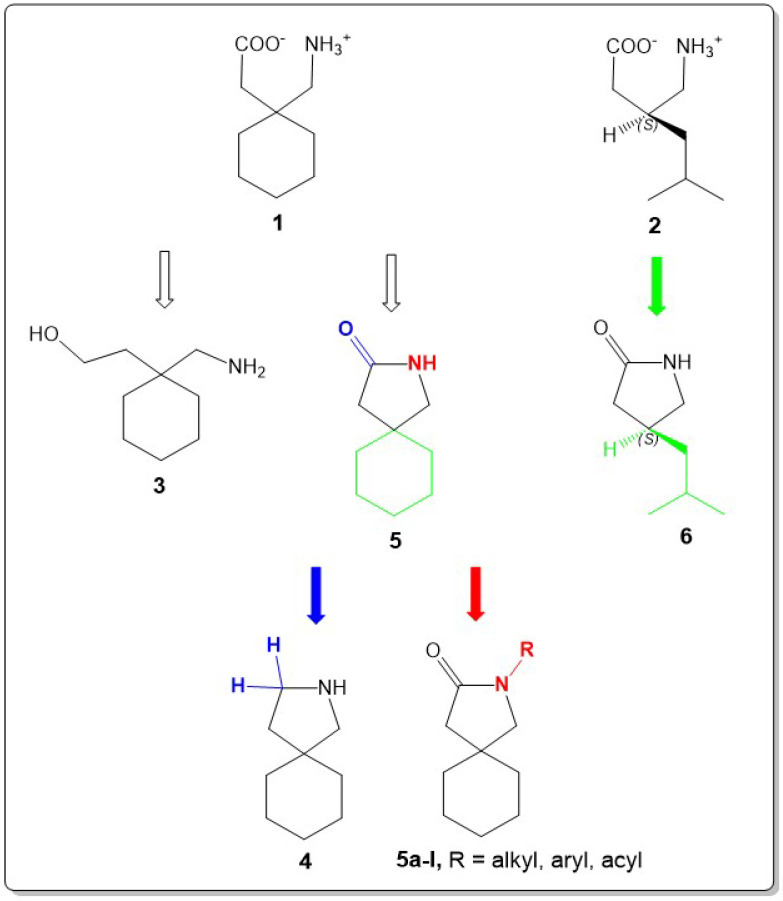
Compounds of interest prepared from **1** and **2** described in this work. The sites susceptible to modification in **5** are indicated in colors. Blue: reduction of the carbonyl group; red: nitrogen alkylation; green: change from a spirocycle to an open linear chain.

Gabapentin **1** and pregabalin **2** (Scheme 1) are two γ-aminobutyric acid (GABA) analogs that have been indicated for the treatment of epilepsy, peripheral neuropathic pain, diabetic neuropathy, and postherpetic neuralgia [20]. Both exist as zwitterions in their solid state [21,22,23]. Furthermore, gabapentin exhibits different polymorphs [21,22]. Only one hydrate form (Form I) of gabapentin is known to date. The anhydrous commercial form (Form II) is the most stable, while Form I is more stable in the presence of water [24]. In the last four years, the prescription of these drugs has increased by 50% in some countries, to the point of being the two most used active ingredients within their therapeutic group [25,26]; therefore, it is expected that their presence as chemical waste in the environment will increase.

The importance of recycling **1** and **2** using simple chemical transformations is that it leads to attractive chemical reagents for organic synthesis and medicinal chemistry, such as aminoalcohol **3**, azaspirocycle compounds **4** and **5,** and the pregabalin lactam **6** (Scheme 1). Medicinal chemists are interested in new lead compounds and structural platforms for drug discovery, as well as methods that can be used to improve existing drugs by obtaining analogs similar in size and shape. There is interest in the little-explored chemical space that includes more conformationally restricted three-dimensional compounds, such as azaspirocycles, with two rings that fuse at a quaternary carbon atom [27], in contrast to the relatively planar structures that are used most [28]. 

**Figure 1:**
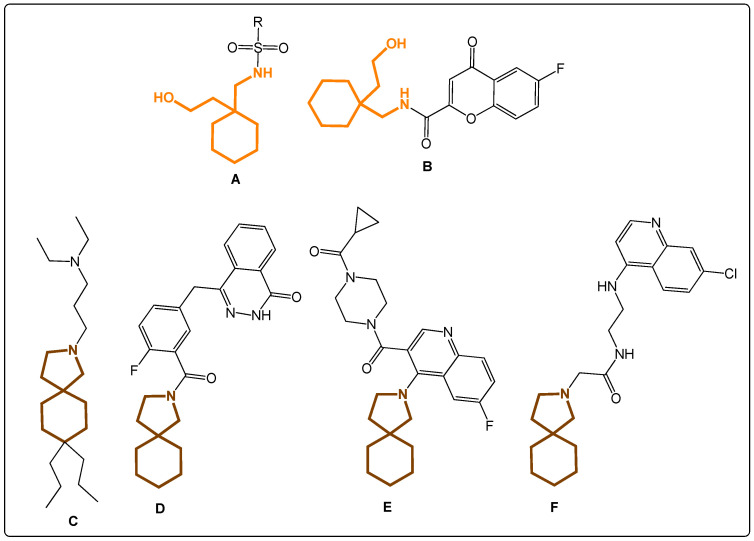
Aminoalcohol **3**- and azaspiroamine **4**-containing analogs described in the literature.

Aminoalcohol **3** has been used as a chemical reagent for the synthesis of **A** [29] and anti-infective agent **B** (Figure 1) [30]. Spiroamine **4** (Scheme 1) has been studied as an antiviral [31] and as a structural motif for the synthesis of various biologically active compounds (Figure 1), e.g., the antitumor drug Atiprimod **C** [32], the analog of the cancer treatment drug Olaparib **D** [33], the quinoline-based aldehyde dehydrogenase 1A1 inhibitor **E** [34], and antiplasmodial compound **F** [35].

**Figure 2:**
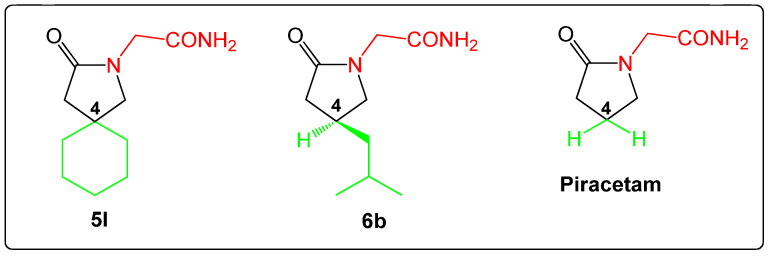
Some compounds of interest (**5l** and **6b**) described in this work.

Spirolactam **5** has interesting and potentially useful biological activities, e.g., it has been isolated as a natural product with antifeedant, larvicidal and pupicidal properties [36], it is a K^+^ channel activator, it possess neuroprotective effects in degenerative disorders such as Huntington’s disease [37,38,39], it is neurotrophic [40], and it enhances new bone formation in vitro [41]. It is also useful for the preparation of *N*-derivative spirolactams, e.g., compound **5l** (R = CH_2_CONH_2_, Scheme 1), which has been studied as an *N*-methyl-D-aspartate (NMDA) receptor modulator [42] due to its analogous structural to the cognitive enhancer drug piracetam (Figure 2) [43]. 

It is estimated that in 2050 the population group aged 60 years or more will be more than 4 billion [44]. Therefore, based on the pharmacological properties of piracetam and the biological profile of **5**, the synthesis of *N*-derivatives such as **5l** and **6b** (Figure 2) could contribute to the discovery of new, more specific and/or powerful drugs, responding to the needs of people with chronic health problems, such as Huntington’s neurodegeneration, Parkinson’s disease, or chronic pain.

Therefore, with this work, we wish to show how the use of recovered active ingredients **1** and **2** as starting materials can lead to promising substrates, endowed with functionalities and structural three-dimensionality, as reagents for organic synthesis and/or of possible interest in the field of medicinal chemistry.

## 2. Results and Discussion

Gabapentin **1** and pregabaline **2** were extracted with methanol from expired tablets and capsules, respectively. Gabapentin **1** was obtained as a white solid (95% yield), along with a very small amount (<1%) of the excipient macrogol 4000 (polyethylene glycol 4000), which was co-extracted from the tablets. Our gabapentin had a melting point of 154–156 °C, agreeing with the mono-hydrate form of gabapentin [45]. Pregabalin **2** was slightly impure and contained excipients; however, this did not pose any problems in the next step.

**Scheme 2:**
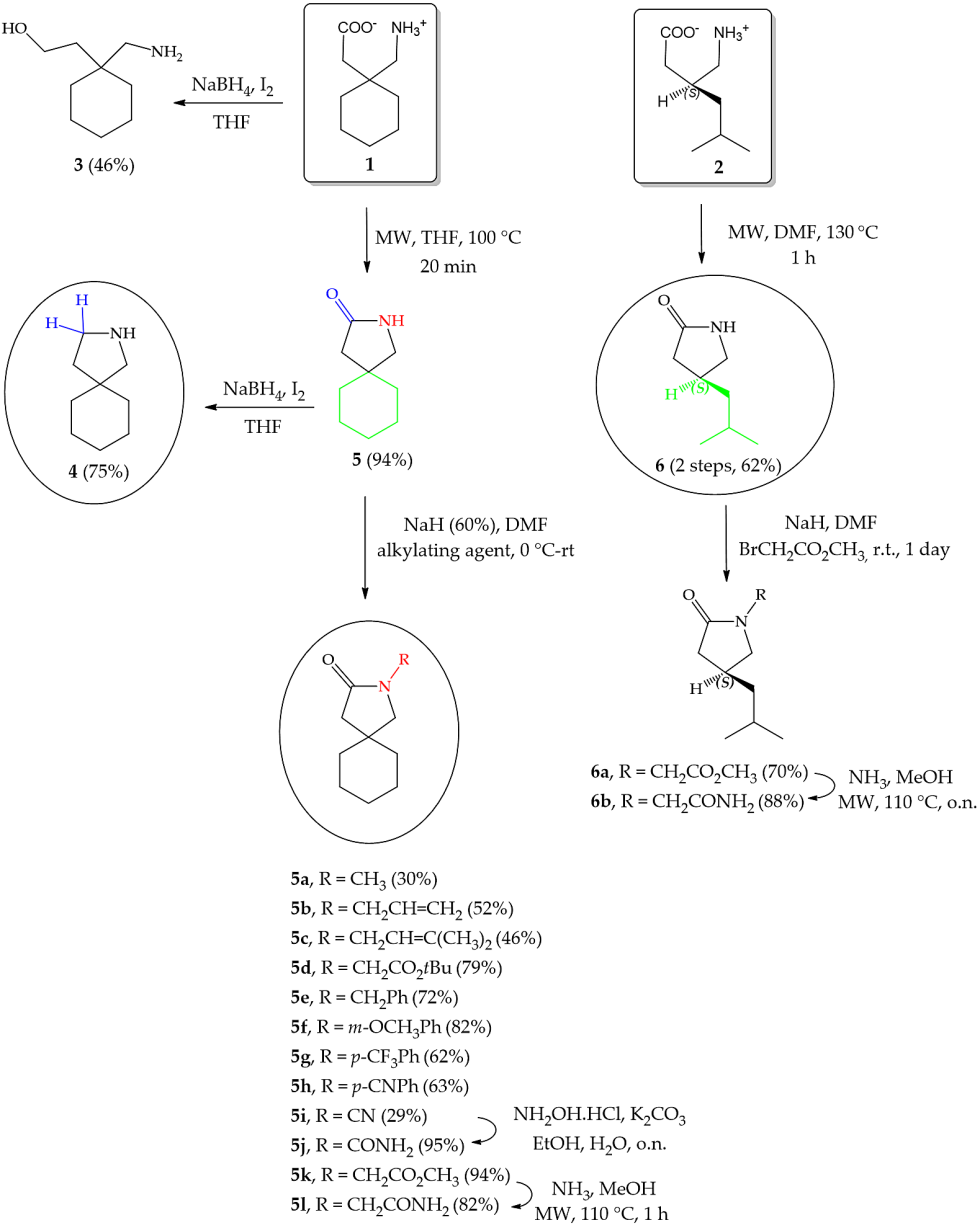
Synthesis of substrates of interest.

To reduce aminoacids to aminoalcohols, the lithium aluminum hydride (LiAlH_4_) procedure is one of the most widely used techniques; however, a safer and simpler process was used, as described in the literature for the reduction of other types of amino acids (ω- and α-amino acids) [46,47]. Thus, aminoalcohol **3** was prepared from **1** using NaBH_4_ and I_2_. A yield of 46% was obtained, along with a 17% yield of azaspiroamine **4**. The reaction was carried out at room temperature because lower yields were obtained when heated (Scheme 2). Although **3** has been used previously as a chemical reagent, its synthesis has not been mentioned [29,30].

Several synthetic routes to the bioactive lactam **5** have been described in the literature [48,49,50,51,52,53,54,55,56,57,58]. They generally require several steps and use hazardous reagents such as Raney nickel [55] or sodium cyanide [50]. Gabapentin **1** is known to convert from its zwitter-ionic form to its more stable lactam, even in its solid state, under either thermal or mechanical stress [59,60,61,62,63]. In the solution phase, the kinetics of the formation of **5** in water (80 °C, various pHs) has been studied [64]. The authors, based on previous cyclization studies of other amino acids [65,66], described the conversion of gabapentin to the lactam as proceeding via an initial intramolecular attack of the amine, in the neutral form of gabapentin, on the carbonyl group, forming a tetrahedral intermediate, which upon the loss of water, generates the lactam. Under these reaction conditions, the lactam is stable, and it is this irreversibility that converts the reversible forms of gabapentin into the lactam. Unfortunately, no yields or isolation procedures to obtain the lactam were reported in the publication. Given this information, we investigated whether it would be possible to convert **1** into **5** using an appropriate organic solvent and thermal conditions. The premise was that the small amounts of gabapentin, present in its neutral form, would be soluble in the organic solvent and would be rapidly converted to a stable lactam. The equilibrium between the various ionic and neutral forms of gabapentin would be continually re-established, driving the reaction towards the stable lactam. THF was chosen as our candidate solvent. Reactions were performed in sealed tubes, which were heated using microwave irradiation. These conditions were chosen as they allowed us to vary the temperature of the reaction more easily and heat the mixture above its boiling point if required. Although gabapentin has very low solubility in THF at room temperature, we noticed that when using temperatures of 100 °C or above, a clear solution resulted after only 20 min. Upon cooling to room temperature, white crystals formed. Spectroscopic and physical evidence confirmed the new crystals to be the desired lactam **5** (Scheme 2). ^1^H NMR and LC-MS analysis conducted during the reaction (see Supplementary Materials) confirmed the rapid and clean conversion of **1** to **5**. Under these conditions, **5** was reproducible and generated in high yields (94–100%) with a high purity (typically > 96% HPLC). Furthermore, the reaction could be easily scaled to generate multigram quantities using 20 mL vials. As expected, the reaction also occurred using non-MW irradiation conditions; however, interestingly, it required much longer reaction times (>4 h versus 20 min). To the best of our knowledge, this is the first time the lactamization of **1** has been reported in an organic solvent. Crude pregabalin **2** was also successfully cyclized to **6** using the same microwave-induce heating procedure. In this case, the use of DMF was necessary to obtain good yields (62% yield with two steps: extraction and lactamization). In terms of upcycling, it must be noted that 6 is an expensive and fine chemical intermediate. To investigate the generality of this cyclization method, we used the same reaction conditions with the parent amino acid (γ-aminobutanoic acid, GABA). In THF (130 °C, 30 min, MW), no cyclization was observed (^1^H NMR); however, a 40% conversion was observed when using DMF (130 °C, 50 min, MW), with the remaining material being GABA (^1^H NMR). These results indicate that although it is possible to cyclize GABA under these conditions, gabapentin and pregabalin are more susceptible to these conditions, which is probably due to a combination of their increased solubility in organic solvents and their substituents (gem-dimethyl effect).

The structural analogs of the bioactive lactam **5** were synthesized, taking into account the sites susceptible to modification (Scheme 1). Although azaspiroamine **4** has been previously prepared from **5** with LiAlH_4_ [47], we synthetized it with a good yield following the same reduction procedure used to obtain aminoalcohol **3**. In this case, heating was required (Scheme 2). A small collection of *N*-analogs **5a-l** was synthetized with moderate to good yields according to a known procedure [67], consisting of deprotonation of 5 with NaH followed by the alkylation of nitrogen with different alkylating agents (Scheme 2). Attempts at the *N*-acylation of **5** with acid chlorides turned out to be unsuccessful; however, we were able to prepare the *N*-cyano derivative **5i**, which can give access to *N*-acyl derivatives, e.g., carboxamide **5j** was obtained via the treatment of **5i** with NH_2_OH.

The *N*-alkylation of **6** with NaH and methyl 2-bromoacetate gave **6a**. The treatment of **5k** and **6a** with a solution of NH_3_ in MeOH afforded two-piracetam analogs, **5l** and **6b**, with good yields (Scheme 2).

**Scheme 3:**
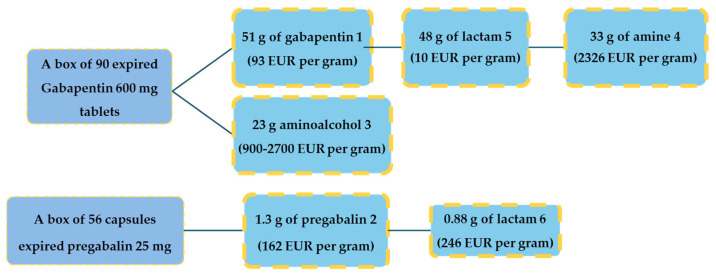
Revaluation of the expired medications used in this work.

The outcome of this work indicates that the chemical recycling of two expired drugs, which are essentially chemical waste, leads to structures that have chemical and biological interest at a lower cost, and for some of them, these costs are cheaper than their original prices. Scheme 3 shows an estimate of the grams obtainable from a box of expired gabapentin and pregabalin based on the yields of the chemical procedures used in this work. Their prices, shown in brackets, are approximate as they can vary depending on purity and chemical supplier. Even in cases where the price is not prohibitive, the search for synthesis from low-cost substrates and that require few steps will be worth it.

## 3. Materials and Methods

### 3.1. General Methods

The expired drugs Gabapentin Sandoz^®^ 600 mg (Sandoz Farmacéutica, S.A. Centro Empresarial Osa Mayor, Avda. Osa Mayor, 4, 28023 Aravaca, Madrid, Spain) and Lyrica^®^ 25 mg (Pfizer. Upjohn EESV, Rivium Westlaan 142, 2909 LD Capelle aan den IJssel, The Netherlands) were obtained from pharmacies. All of the reagents and solvents were obtained from Aldrich Chemical Co. and used without further purification. Reactions with sensitive reagents were performed under an inert atmosphere (argon or nitrogen), and organic solvents were dried using standard methods. Unless otherwise stated, solvents were removed under reduced pressure using a rotary evaporator at 40–60 °C. All of the reactions were monitored via analytical thin layer chromatography (TLC) on POLIGRAM^®^ SIL G/ UV_254_ silica gel-coated plates (0.20 mm) from MACHEREY-NAGEL (Düren, Germany). Column chromatography was performed on silica gel 60 (0.063–0.20 mm) from MERCK (Rahway, NJ, USA). Preparative thin layer chromatography was carried out with GF silica gel plates (1 mm) with fluorescent indicator 254 nm (UNIPLATE). The compounds were visualized using ultraviolet light (254 nm). The purity of the final compounds was determined to be ≥96% via high-pressure liquid chromatography (HPLC) using a Jasco PU-2080 intelligent HPLC pump (Jasco MD 2020 Plus multiwavelength detector from Jasco Analítica Spain, S.L., Madrid, Spain). Monitorization of the lactamization reaction of **1** via LC-MS was performed with UPLC Aquity Hclass Waters with a mass spectrometer Vion IMS Qtof Waters. ^1^H and ^13^C NMR spectra were recorded at room temperature (rt) on a Bruker Avance 500 or 600 MHz NMR spectrometer in the solvent indicated. Data for ^1^H NMR are reported as follows: chemical shift (δ ppm), integration, multiplicity and coupling constant (Hz). ^13^C NMR analyses are reported in terms of chemical shift. Melting points were determined using a Stuart Scientific SMP11 instrument. Low-resolution (EIMS) and high-resolution mass spectrometry (HRMS) were performed on a Micromass AutoSpec magnetic tri-sector (EBE geometry) mass spectrometer. Microwave reactions were performed using a Biotage^®^ Initiator (software version 2.5). Lyophilization was performed using a CHRIST ALPHA 2-4 lyophilizer.

### 3.2. Extraction

#### 3.2.1. Extraction of 2-(1-(Aminomethyl)cyclohexyl)acetic Acid (**1**)

Each tablet of Gabapentin Sandoz^®^ 600 mg contains 600 mg of gabapentin **1** and 113 mg of excipient (macrogol 4000, pregelatinized cornstarch, colloidal anhydrous silica, magnesium stearate, polyvinyl alcohol, titanium dioxide, talc, lecithin, and xanthan gum). For the extraction of **1**, four of these tablets were crushed in a mortar. Next, the solid was suspended in MeOH (240 mL) and filtered. The filtering operation was repeated until a transparent solution was obtained. After removing the solvent, 2.3 g (95% yield) of a white solid was obtained. ^1^H NMR spectrum (CD_3_OD) showed the characteristic signals of **1**. ^1^H NMR (500 MHz, CD_3_OD) δ: 2.90 (2 H, s, H-1′), 2.47 (2 H, s, H-2), 1.59–1.54 (6 H, m, H-2″, 2 × H-3″, H-4″, 2 × H-5″ and H-6″), 1.41–1.39 (4 H, m, H-2″, H-4″ and H-6″). ^13^C NMR (125 MHz, CD_3_OD) δ: 178.53 (s, C-1), 48.85 (t, C-1′), 46.72 (t, C-2), 34.35 (s, C-1″), 33.65 (t, C-2″ and C-6″), 25.57 (t, C-4″), and 21.05 (t, C-3″ and C′-5″). Mp: 154–156 °C.

#### 3.2.2. Extraction of (S)-3-(Aminomethyl)-5-Methylhexanoic Acid (**2**)

Each Lyrica^®^ 25 mg capsule contains 25 mg of active ingredient pregabalin **2** and 74.4 mg of excipients (lactose monohydrate, cornstarch, and talc). For the extraction of 2, 14 capsules of 25 mg were used. The contents of the capsules were suspended in 30 mL of MeOH, heated in an oil bath at 50 °C for 15 min and filtered. The filtering operation was repeated until a transparent solution was obtained. After removing the solvent, crude **2** was obtained (320 mg) as a white solid. ^1^H NMR spectrum (CD_3_OD) showed the characteristic signals of **2**. ^1^H NMR (500 MHz, CD_3_OD) δ: 2.98 (1 H, dd, J_1_ = 3.5 Hz, J_2_ = 12.8 Hz, H-1′), 2.86 (1 H, dd, J_1_ = 8.0 Hz, J_2_ = 12.8 Hz, H-1′), 2.45 (1 H, dd, J_1_ = 3.3 Hz, J_2_ = 15.7 Hz, H-2), 2.28 (1 H, dd, J_1_ = 8.7 Hz, J_2_ = 15.7 Hz, H-2), 2.1 (1 H, m, H-3), 1.71 (1 H, m, H-5), 1.28 (1 H, m, H-4), 1.21 (1 H, m, H-4), 0.96 (3 H, d, J = 7.4 Hz, CH_3_), and 0.94 (3 H, d, J = 6.9 Hz, CH_3_).

### 3.3. Synthesis

#### 3.3.1. Synthesis of 2-(1-(Aminomethyl)cyclohexyl)ethanol (**3**)

Gabapentin **1** (200 mg, 1.17 mmol) was added in one portion to a suspension of NaBH_4_ (132 mg, 3.5 mmol) in anhydrous THF (9 mL) under an argon atmosphere. The reaction mixture was cooled to 0 °C in an ice bath. A solution of I_2_ (900 mg, 3.5 mmol) in anhydrous THF (4 mL) was added slowly over 30 min, resulting in vigorous hydrogen evolution. When the addition of iodine was complete and gas evolution ceased, the flask containing the reaction mixture was stirred at room temperature. After 24 h, 2 more equivalents of NaBH_4_/I_2_ were added, and the reaction mixture was stirred for another 24 h. Methanol was then added cautiously until the mixture became clear. After stirring for 30 min, the solvent was removed in the rotary evaporator, leaving a white paste that was dissolved in 11 mL of 20% aqueous KOH. The solution was stirred for 4 h and extracted with CH_2_Cl_2_ (3 × 10 mL). The organic extracts were dried with Na_2_SO_4_ and concentrated under vacuum. The crude oil was purified via silica gel column chromatography (hexane/EtOAc, 7:3, 3:2, 1:1), obtaining product **3** (85 mg, 46%) as a white solid and compound **4** (28 mg, 17%) as a colorless oil. ^1^H NMR (500 MHz, CDCl_3_) δ: 4.23 (2 H, brs, NH_2_), 3.75 (2 H, t, J = 5.15 Hz, H-1), 2.70 (2 H, m, H-1′), 2.49 (1 H, brs, OH), 1.61 (2 H, d, J = 5.3 Hz, H-2), 1.44 (6H, m, cyclohexyl), 1.34 (4 H, m, cyclohexyl). ^13^C NMR (125 MHz, CDCl_3_) δ: 57.86 (t, C-1), 56.70 (t, C-1′), 38.46 (s, C-2), 35.28 (s, C-1″), 34.46 (t, 2 × C, C-2″ and C-6″), 26.02 (t, C-4″), and 21.11 (t, 2 × C, C-3″ and C-5″). Mp: 68–70 °C.

#### 3.3.2. Synthesis of 2-Azaspiro [4.5]Decane (**4**)

Lactam **5** (39 mg, 0.25 mmol) was added in one portion to a reaction flask containing NaBH_4_ (30 mg, 0.78 mmol) and anhydrous THF (5 mL) under an argon atmosphere. The reaction mixture was cooled to 0 °C in an ice bath. A solution of I_2_ (200 mg, 0.78 mmol) in anhydrous THF (1 mL) was added slowly, resulting in vigorous hydrogen evolution. When the addition of iodine was complete and gas evolution ceased, the flask containing the reaction mixture was heated at reflux for 18 h. It was then cooled to room temperature, and methanol was added cautiously until the mixture became clear. After stirring for 30 min, the solvent was removed under vacuum, leaving a white paste that was dissolved in 2 mL of 20% aqueous KOH. The solution was stirred for 4 h and extracted with CH_2_Cl_2_ (3 × 5 mL). The organic extracts were dried with Na_2_SO_4_ and concentrated under vacuum. The crude oil was purified via silica gel column chromatography (hexane/EtOAc, 7:3, 3:2, 1:1), obtaining product **4** (26 mg, 75%) as a colorless oil. ^1^H NMR (500 MHz, CDCl_3_) δ: 4.69 (1 H, brs, NH), 3.24 (1 H, m, H-3), 3.04 (1 H, dd, J_1_ = 5.9 Hz, J_2_ = 12.1 Hz, H-3), 2.74 (1 H, m, H-1), 2.43 (1 H, dd, J_1_ = 10.3 Hz, J_2_ = 12.0 Hz, H-1), 1.71 (1 H, m, H-4), 1.62 (1 H, m, H-4), 1.42- 1.34 (10 H, m, 2 × H-6, 2 × H-7, 2 × H-8, 2 × H-9 and 2 × H-10). ^13^C NMR (125 MHz, CDCl_3_) δ: 65.19 (t, C-3), 53.42 (t, C-1), 43.17 (t, C-4), 37.38 (s, C-5), 37.02 (t, C-6 or C-10), 36.88 (t, C-6 or C10), 25.63 (t, C-8), 23.62 (t, C-7 or C-9), 23.15 (t, C-7 or C-9). EIMS: m/z 150 (100), 152 (98), and 139 (M^+^, 36). HRMS: calcd. for C_9_H_17_N (M^+^) 139.1361, found 139.1358.

#### 3.3.3. Synthesis of 2-Azaspiro [4.5]Decan-3-One (**5**)

A solution of **1** (1 g, 5.8 mmol) in anhydrous THF (15 mL) was microwaved at 100 °C for 20 min. Once room temperature was reached, the crystallized product was filtered, washed with hexane and dried, obtaining product **5** (0.85 g, 95%) as a white solid. ^1^H NMR (600 MHz, CDCl_3_) δ: 6.28 (1H, brs, NH), 3.19 (2H, s, H-1), 2.23 (2H, s, H-4), 1.55-1.48 (10H, m). ^13^C NMR (150 MHz, CDCl_3_) δ: 178.1 (s, C-3), 53.8 (t, C-1), 43.03 (t, C-4), 39.50 (s, C-5), 36.82 (t, 2 × C), 25.60 (t, C-8), and 22.85 (t, 2 × C). Mp: 89–90 °C

#### 3.3.4. Synthesis of Derivatives of **5a-5i** and **5k**. General Procedure

To a solution of **5** (100 mg, 0.65 mmol) in anhydrous DMF at 0 °C, NaH (60% dispersion in mineral oil) was added and stirred at this temperature for 15–20 min under an argon atmosphere. Once room temperature was reached, the alkylating agent was added, and the mixture was stirred at room temperature. EtOAc (5–10 mL) was added and the crude mixture was washed with water (3 × 5–10 mL). The organic phase was dried with anhydrous Na_2_SO_4_, filtered and the solvent was removed under a vacuum. The crude was purified via silica gel column chromatography (hexane/EtOAc, gradient) to give products **5a-5i** and **5k**.

##### Synthesis of 2-Methyl-2-Azaspiro [4.5]Decan-3-One (**5a**)

From 5, the conditions were DMF (4 mL), NaH (104 mg, 2.6 mmol), methyl iodide (0.081 mL, 1.3 mmol), 2 days. Product 5a (33.2 mg, 30%) was obtained as a colorless oil. ^1^H NMR (500 MHz, CDCl_3_) δ: 3.06 (2 H, s, H-1), 2.76 (3 H, s, H-1′), 2.18 (2 H, s, H-4), 1.41-1.34 (10 H, m, H-6 to H-10). ^13^C NMR (125 MHz, CDCl_3_) δ: 174.20 (s, C-3), 61.30 (t, C-1), 43.96 (t, C-4), 37.17 (t, 2 × C, C-6 and C-10), 36.05 (t, C-5), 29.71 (q, C-1′), 25.56 (t, C-8), 22.85 (t, 2 × C, C-7 and C-9). EIMS: m/z 167 (M^+^, 100), 166 (53), and 149 (45). HRMS: calcd. for C_10_H_17_NO (M^+^) 167.1310, found 167.1298.

##### Synthesis of 2-Allyl-2-Azaspiro [4.5]Decan-3-One (**5b**)

From 5, the conditions were DMF (2 mL), NaH (62.65 mg, 2.61 mmol), allyl bromide (254.85 mg, 1.3 mmol) and 2 days. Product **5b** (65 mg, 52%) was obtained as a colorless oil. ^1^H NMR (500 MHz, CDCl_3_) δ: 5.64 (1 H, m, H-2′), 5.10 (2 H, m, H-3′), 3.8 (2 H, d, J = 6 Hz, H-1′), 3.02 (2 H, s, H-1), 2.21 (2 H, s, H-4), 1.40-1.34 (10 H, m, H-6 to H-10). ^13^C NMR (125 MHz, CDCl_3_) δ: 173.73 (s, C-3), 132.57 (d, C-2′), 117.80 (t, C-3′), 58.27 (t, C-1), 45.06 (t, C-1), 44.13 (t, C-4), 37.05 (t, 2 × C, C-6 and C-10), 36.12 (s, C-5), 25.59 (t, C-8), 22.82 (t, 2 × C, C-7 and C-9). EIMS: m/z 193 (M^+^, 70), and 70 (100). HRMS: calcd. for C_12_H_19_NO (M^+^) 193.1467, found 193.1459.

##### Synthesis of 2-(3-Methylbut-2-en-1-Yl)-2-Azaspiro [4.5]Decan-3-One (**5c**)

From **5**, the conditions were DMF (2 mL), NaH (52 mg, 1.3 mmol), 1-bromo-3-methylbut-2-ene (193 mg, 0.15 mL, 1.3 mmol) and 2 days. Product **5c** (66 mg, 0.3 mmol, 46%) was obtained as a colorless oil. ^1^H NMR (500 MHz, CDCl_3_) δ: 5.10 (1 H, t, J = 7.08 Hz, H-2′), 3.86 (2 H, d, J = 7.1 Hz, H-1′), 3.07 (2 H, s, H-1), 2.26 (2 H, s, H-4), 1.74 (3 H, s, CH_3_), 1.70 (3 H, s, CH_3_), 1.49 (10 H, t, H-6 to H-10). ^13^C NMR (125 MHz, CDCl_3_) δ: 173.52 (s, C-3); 136.90 (s, C-3′); 118.58 (d, C-2′); 58.21 (t, C-1); 44.09 (t, C-1′); 40.05 (t, C-4); 37.02 (t, 2 × C, C-6 y C-10); 36.11 (s, C-5); 25.67 (t, C-8); 25.50 (t, 2 × C, C-7 y C-9); 17.84 (q, 2 × CH_3_). EIMS: m/z 221 (M^+^, 37), 206 (71), 166 (76), and 153 (100). HRMS: calcd. for C_14_H_23_NO (M^+^) 221.1780, found 221.1777.

##### Synthesis of Tert-Butyl 2-(3-Oxo-2-Azaspiro [4.5]Decan-2-Yl)Acetate (**5d**)

From **5**, the conditions were DMF (4 mL), NaH (104 mg, 2.6 mmol), *tert*-butyl 2-bromoacetate (507 mg, 0.4 mL, 2.6 mmol) and 2 days. Product **5d** (136 mg, 0.51 mmol, 79%) was obtained as a colorless oil. ^1^H NMR (500 MHz, CDCl_3_) δ: 3.94 (2 H, s, H-2′), 3.23 (2 H, s, H-1), 2.28 (2 H, s, H-4), 1.57-1.46 (19 H, m, H-6 to H-10, *t*Bu). ^13^C NMR (125 MHz, CDCl_3_) δ: 174.54 (s, C-3), 167.74 (s, C-1′), 82.01 (s, OC*t*Bu), 59.10 (t, C-1), 44.70 (t, C-2′), 43.55 (t, C-4), 36.87 (t, 2 × C, C-6 and C-10), 36.40 (s, C-5), 28.07 (q, 3 × CH_3_), 25.59 (t, C-8), 22.84 (t, 2 × C, C-7 and C-9). EIMS: m/z 267 (M^+^, 3.4), 211 (53), 167 (54), and 166 (100). HRMS: calcd. for C_15_H_25_NO_3_ (M^+^) 267.1834, found 267.1841.

##### Synthesis of 2-Benzyl-2-Azaspiro [4.5]Decan-3-One (**5e**)

From **5** (100 mg, 0.65 mmol), the conditions were DMF (4 mL), NaH (104 mg, 2.6 mmol), benzyl bromide (0.3 mL, 2.6 mmol), with a stirring time of 2 days. Product **5e** (114.3 mg, 72%) was obtained as a colorless oil. ^1^H NMR (500 MHz, CDCl_3_) δ: 7.37-7.24 (5 H, m, H-2″ to H-6″), 4.46 (2 H, s, H-1′), 3.02 (2 H, s, H-1), 2.36 (2 H, s, H-4), 1.49-1.37 (10 H, m, H-6 to H-10). ^13^C NMR (125 MHz, CDCl_3_) δ: 174.16 (s, C-3), 136.45 (s, C-1″), 128,67 (d, 2 × C), 128.09 (d, 2 × C), 127.56 (d, C-4″), 58.18 (t, C-1), 46.58 (t, C-1′), 43.98 (t, C-4), 36.91 (t, 2 × C, C-6 and C-10), 36.22 (s, C-5), 25.54 (t, C-8), and 22.77 (t, 2 × C, C-7 and C-9). EIMS: m/z 243 (M^+^, 90), 91 (100). HRMS: calcd. for C_16_H_21_NO (M^+^) 243.1623, found 243.1616.

##### Synthesis of 2-(3-Methoxybenzyl)-2-Azaspiro [4.5]Decan-3-One (**5f**)

From **5** (147.7 mg, 0.964 mmol), the conditions were DMF (2 mL), NaH (50 mg, 1.25 mmol), 3-methoxybenzyl bromide (0.15 mL, 1.07 mmol), with a stirring time of 24 h. Product **5f** (216.9 mg, 82%) was obtained as a colorless oil. ^1^H NMR (600 MHz, CDCl_3_) δ: 7,24 (1 H, t, J = 9,5 Hz, H-5″), 6,82-6,77 (3 H, m, H-4″, H-2″, H-6″), 4,41 (2 H, s, H-1′), 3,78 (3 H, s, OCH_3_), 3,01 (2 H, s, H-1), 2,32 (2 H, s, H-4), 1,48-1,35 (10 H, m). ^13^C NMR (150 MHz, CDCl_3_) δ: 174.07 (s, C-3), 159.85 (s, C-3″), 138.16 (s, C-1″), 129.68 (d, C-5″), 120.33 (d, C-6″), 113.09 (d, C-2″ or C-4″), 113.06 (d, C-2″or C-4″), 55.27 (2 × C), 46.43 (2 × C), 36.86 (t, C-6 and C-10), 36.14 (s, C-5), 25.56 (t, C-8), and 22.76 (t, C-7 and C-9).

##### Synthesis of 2-(4-(Trifluoromethyl)benzyl)-2-Azaspiro [4.5]Decan-3-One (**5g**)

From **5** (100 mg, 0.65 mmol), the conditions were DMF (4 mL), NaH (104 mg, 2.6 mmol), 1-(bromomethyl)-4-(trifluoromethyl)benzene (180 mg, 0.75 mmol) and 2 days. Product **5g** (125 mg, 0.4 mmol, 62%) was obtained as a colorless oil. ^1^H NMR (500 MHz, CDCl3) δ: 7.60 (2 H, d, J = 7.98 Hz), 7.36 (2 H, d, J = 7.9 Hz), 4.50 (2 H, s, H-1′), 3.02 (2 H, s, H-1), 2.34 (2 H, s, H-4), 1.49-1.26 (10 H, m, H-6 to H-10). ^13^C NMR (125 MHz, CDCl3) δ: 174.32 (s, C-3), 140.68 (s, C-4″), 128.27 (s, 5 × C, C1″, C-2″, C-3″, C-5″ and C-6″), 125.65 (s, CF_3_), 58.30 (t, C-1), 46.12 (t, C-1′), 43.76 (t, C-4), 36.92 (t, 2 × C, C-6 and C-10), 36.27 (s, C-5), 25.49 (t, C-8), and 22.74 (t, 2 × C, C-7 and C-9). EIMS: m/z 311 (M^+^, 100), 159 (53). HRMS: calcd. for C_17_H_20_NOF_3_ (M^+^) 311.1497, found 311.1483.

##### Synthesis of 4-((3-Oxo-2-Azaspiro [4.5]Decan-2-Yl)methyl)benzonitrile (**5h**)

From **5** (100 mg, 0.65 mmol), the conditions were DMF (2 mL), NaH (62.65 mg, 2.61 mmol), 4-cyanobenzyl bromide (254.85 mg, 1.3 mmol) and 2 days. Product **5h** (110.3 mg, 63%) was obtained as a colorless oil. ^1^H NMR (500 MHz, CDCl_3_) δ: 7.64 (2 H, d, J = 8.1, H-2″ and H-6″), 7.35 (2 H, d, J = 8.1, H-3″ and H-5″), 4.49 (2 H, s, H-1′), 3.02 (2 H, s, H-1), 2.34 (2 H, s, H-4), 1.51-1.37 (10 H, m, H-6 to H-10). ^13^C NMR (125 MHz, CDCl_3_) δ: 174.36 (s, C-3), 142.17 (s, C-1″), 132.54 (d, 2 × C, C-2″ and C-6″), 128.60 (d, 2 × C, C-3″ and C-5″), 118.55 (s, CN or C-4″), 111.60 (s, CN or C-4″), 58.38 (t, C-1), 46.23 (t, C-1′), 43.69 (t, C-4), 36.94 (t, 2 × C, C-6 and C-10), 36.32 (s, C-5), 25.47 (t, C-8), 22.74 (t, 2 × C, C-7 and C-9). EIMS: m/z 268 (M^+^, 100), and 116 (35). HRMS: calcd. for C_17_H_20_N_2_O (M^+^) 268.1576, found 268.1591.

##### Synthesis of 3-Oxo-2-Azaspiro [4.5]Decane-2-Carbonitrile (**5i**)

From **5** (500 mg, 3.26 mmol), the conditions were DMF (16 mL), NaH 60% (260 mg), cyanogen bromide (690 mg, 6.52 mmol) and 1h. Product **5i** (167 mg, 29%) was obtained as a colorless oil. ^1^H NMR (500 MHz, CDCl_3_) δ: 3.49 (2 H, s, H-1), 2.27 (2 H, s, H-4), 1.49-1.40 (10 H, m, H-6 to H-10). ^13^C NMR (125 MHz, CDCl_3_) δ: 174.29 (s, C-3), 107.29 (s, CN), 58.10 (t, C-1), 41.72 (t, C-4), 38.65 (s, C-5), 35.96 (t, C-6 a C-10), 25.20 (t, C-8), 22.52 (t, 2 × C, C-7 and C-9). EIMS: m/z 178 (M^+^, 39), 123 (100), and 96 (40). HRMS: calcd. for C_10_H_14_N_2_O (M^+^) 178.1106], found 178.1106.

##### Synthesis of Methyl 2-(3-Oxo-2-Azaspiro [4.5]Decan-2-Yl)Acetate (**5k**)

From **5**, the conditions were DMF (4 mL), 60% NaH (54 mg, 2.25 mmol), methyl 2-bromoacetate (198 mg, 0.124 mL, 1.3 mmol) and 2 days. EtOAc (5 mL) was added to the reaction crude and washed with water (3 × 4 mL). Product **5k** (138.6 mg, 94%) was obtained as a colorless oil. ^1^H NMR (500 MHz, CDCl_3_) δ: 4.06 (2 H, s, H-2′), 3.74 (3 H, s, OCH_3_), 3.24 (2 H, s, H-1), 2.30 (2 H, s, H-4), 1.55-1.44 (10 H, m, H-6 to H-10). ^13^C NMR (125 MHz, CDCl_3_) δ: 174.83 (s, C-3), 169.15 (s, C-1′), 59.12 (t, C-1), 52.16 (s, OCH_3_), 43.86 (t, C-2′), 43.52 (t, C-4), 36.82 (t, 2 × C, C-6 and C-10), 36.60 (s, C-5), 25.58 (t, C-8), 22.83 (t, 2 × C, C-7 and C-9). EIMS: m/z 225 (M^+^, 55), 166 (100), and 152 (57). HRMS: calcd. for C_12_H_19_NO_3_ (M^+^) 225.1365, found 225.1358.

#### 3.3.5. Synthesis of 3-Oxo-2-Azaspiro [4.5]Decane-2-Carboxamide (**5j**)

To a solution of **5i** (25 mg, 0.134 mmol) in EtOH (1 mL), K_2_CO_3_ was added (56 mg, 0.4 mmol), followed by the addition of a solution of NH_2_OH.HCl (18.6 mg, 0.268 mmol) in H_2_O (0.5 mL). The reaction mixture was stirred at room temperature overnight. Then, H_2_O (2 mL) was added, and the aqueous phase was extracted using EtOAc (3 × 2 mL). The organic extracts were washed with brine, dried with Na_2_SO_4_ and concentrated under a vacuum. The crude mixture was purified via silica gel column chromatography (hexane/EtOAc, 4:1, 3:2, 1:1) to obtain **5j** (25 mg, 95%) as a white solid. ^1^H NMR (500 MHz, CDCl_3_) δ: 8.22 (1 H, brs, NH), 5.18 (1 H, brs, NH), 3.64 (2 H, s, H-1), 2.48 (2 H, s, H-4), 1.55-1.48 (10 H, m, H-6 to H-10). ^13^C NMR (125 MHz, CDCl_3_) δ: 176.29 (s, C-3), 153.47 (s, CONH_2_), 55.92 (t, C-1), 45.84 (t, C-4), 36.31 (t, 2 × C, C-6 and C-10), 34.90 (t, C-5), 25.46 (t, C-8), and 22.64 (t, 2 × C, C-7 and C-9). EIMS: m/z 196 (M^+^, 100), 153 (28), 140 (38). HRMS: calcd. for C_10_H_16_N_2_O2 (M^+^) 196.1212, found 196.1198.

#### 3.3.6. Synthesis of 2-(3-Oxo-2-Azaspiro [4.5]Decan-2-Yl)acetamide (**5l**)

**5k** (15 mg, 0.066 mmol) and NH_3_ (2 mL, 4 M in MeOH) were added to a sealed tubed, and then the reaction mixture was irradiated with microwave radiation at 100 °C for 1 h and concentrated. The residue was purified via recrystallization from CH_2_Cl_2_/hexane to afford **5l** (11.4 mg, 82% yield) as a white solid. ^1^H NMR (500 MHz, CDCl_3_) δ: 6.20 (1 H, brs, NH), 5.65 (1 H, brs, NH), 3.84 (2 H, s, H-2′), 3.20 (2 H, s, H-1), 2.22 (2 H, s, H-4), 1.49-1.40 (10 H, m, H-6 to H-10). ^13^C NMR (125 MHz, CDCl_3_) δ: 175.13 (s, C-3), 170.70 (s, C-1′), 59.85 (t, C-1), 46.81 (t, C-2′), 43.50 (t, C-4), 36.88 (t, 2 × C, C-6 and C-10), 36.58 (s, C-5), 25.50 (t, C-8), and 22.78 (t, 2 × C, C-7 and C-9).

#### 3.3.7. Synthesis of (S)-4-Isobutylpyrrolidin-2-One (**6**)

A solution of crude **2** (122 mg, 0.766 mmol) in anhydrous DMF (5 mL) was microwaved at 130 °C for 1 h. Once room temperature was reached, the solvent was lyophilized and the crude reaction was purified via silica gel column chromatography (hexane/EtOAc, 3:1; 1:1; EtOAc) to give **6** (74 mg, 62%, two steps) as a colorless oil. ^1^H NMR (500 MHz, CDCl_3_) δ: 6.13 (1 H, brs, NH), 3.42 (1 H, t, J = 8.6 Hz, H-5), 2.93 (1 H, dd, J_1_ = 7.4 Hz, J_2_ = 9.1 Hz, H-5), 2.48 (1 H, m, H-4), 2.35 (1 H, dd, J_1_ = 8.6 Hz, J_2_ = 16.6 Hz, H-3), 1.92 (1 H, dd, J_1_ = 8.5 Hz, J_2_ = 16.6 Hz, H-3), 1.50 (1 H, m, H-2′), 1.28 (2 H, m, H-1′), 0.84 (3 H, d, J = 6.0 Hz, CH_3_), 0.82 (3 H, d, J = 6.0 Hz, CH_3_). ^13^C NMR (125 MHz, CDCl_3_) δ: 178.55 (s, C-2), 48.27 (t, C-5), 43.88 (d, C-3), 36.99 (d, C-4), 33.04 (d, C-2′), 26.17 (t, C-1′), 22.7 (q, CH_3_), 22.5 (q, CH_3_). EIMS: m/z 142 (55), 141 (M^+^, 100), and 111 (61). HRMS: calcd. for C_8_H_15_NO (M^+^) 141.1154, found 141.1148.

#### 3.3.8. Synthesis of (S)-Methyl 2-(4-Isobutyl-2-Oxopyrrolidin-1-Yl)Acetate (**6a**)

To a solution of **6** (114 mg, 0.807 mmol) in anhydrous DMF (4 mL), NaH was added (60% dispersion in mineral oil) (90 mg, 2.25 mmol) and stirred at room temperature under an argon atmosphere for 20 min. Next, methyl 2-bromoacetate (198 mg, 0.124 mL, 1.3 mmol) was added, and the mixture was stirred at room temperature for 12 h. The solvent was lyophilized, and the reaction crude was purified via silica gel column chromatography (hexane/EtOAc, 4:1; 1:1) to give **6a** (118 mg, 70%) as a colorless oil. ^1^H NMR (500 MHz, CDCl_3_) δ: 4.08 (1 H, d, J = 17.6 Hz, H-2), 4.01 (1 H, d, J = 17.6 Hz, H-2), 3.73 (3 H, s, OCH_3_), 3.52 (1 H, dd, J_1_ = 8.0 Hz, J_2_ = 8.9 Hz, H-5′), 3.11 (1H, dd, J_1_ = 7.0 Hz, J_2_ = 9.0 Hz, H-5′), 2.50 (1 H, dd, J_1_ = 8.6 Hz, J_2_ = 16.0 Hz, H-3′), 2.49 (1 H, m, H-4′), 2.10 (1 H, dd, J_1_ = 7.3 Hz, J_2_ = 16.0 Hz, H-3′), 1.50 (1 H, m, H-2″), 1.36 (2 H, m, H-1″), 0.91 (3 H, d, J = 6.6 Hz, H-3″), 0.90 (3 H, d, J = 6.6 Hz, H-3″). ^13^C NMR (125 MHz, CDCl_3_) δ: 175.18 (s, C-2′), 169.18 (s, C-1), 53.73 (t, OCH_3_), 52.14 (t, C-2), 43.84 (t, C-5′), 43.82 (t, C-3′), 37.25 (d, C-4′), 29.99 (t, C-2″), 26.13 (d, C-1″), 22.66 (q, C-3″), and 22.50 (q, C-3″). EIMS: m/z 236 (M^+^ + Na, 100), 214 (52). HRMS: calcd. for C_11_H_19_NO_3_Na (M^+^ + Na) 236.1263, found 236.1258.

#### 3.3.9. Synthesis of (S)-2-(4-Isobutyl-2-Oxopyrrolidin-1-Yl)acetamide (**6b**)

**6a** (28 mg, 0.13 mmol) and NH_3_ (7 N en MeOH, 1 mL) were added to a sealed tube. Then, the reaction mixture was heated to reflux at 110 °C overnight. The solvent was removed under vacuum, and the residue was purified via silica gel column chromatography (hexane/EtOAc, 1:1, 1:2, EtOAc) to obtain **6b** (22.6 mg, 88%) as a white solid. ^1^H NMR (500 MHz, CDCl_3_): δ: 6.18 (1 H, brs, NH), 5.60 (1 H, brs, NH), 3.88 (1 H, d, J = 15.8 Hz, H-2), 3.82 (1 H, d, J = 15.8 Hz, H-2), 3.52 (1 H, dd, J_1_ = 8.4 Hz, J_2_ = 17.2 Hz, H-5′), 3.06 (1 H, dd, J_1_ = 7.3 Hz, J_2_ = 14.1 Hz, H-5′), 2.48 (1 H, dd, J_1_ = 8.6 Hz, J_2_ = 16.1 Hz, H-3′), 2.43 (1 H, m, H-4′), 2.02 (1 H, dd, J_1_ = 16.1 Hz, J_2_ = 7.6 Hz, H-3′), 1.51 (1 H, m, H-2″), 1.29 (2 H, ddd, J_1_ = 14.0 Hz, J_2_ = 7.0 Hz, J_3_ = 2.9 Hz, H-1″), 0.84 (3 H, d, J = 6.0 Hz, H-3″), 0.83 (3 H, d, J = 6.0 Hz, H-3″). ^13^C NMR (125 MHz, CDCl_3_) δ: 175.6 (s, C-2′), 170.6 (s, C-1), 54.48 (t, C-5′), 46.78 (t, C-2), 43.84 (t, C-1″), 37.36 (t, C-3′), 30.10 (d, C-4′), 26.15 (d, C-2″), 22.66 (q, C-3″), 22.50 (q, C-3″). EIMS: m/z 236 (26), and 221 (M^+^ + Na, 100). HRMS: calcd. for C_10_H_18_N_2_O_2_Na (M^+^ + Na) 221.1266, found 221.1267.

## 4. Conclusions

Expired and unwanted pharmaceuticals, which are essentially chemical waste, have been ignored as a large source of valuable chemicals. Most pharmaceuticals are costly to make, and as such, are classified as high-value chemicals. Furthermore, their synthesis is generally a lot more expensive and generates more waste products than their recovery from drug products (tablets, capsules, etc.) Moreover, many recovered drugs can be reutilized as building blocks, fragments, or reagents for the generation of new chemical entities. In this paper, we have demonstrated these possibilities. We have extracted and upcycled two expired active ingredients and converted them into a collection of interesting pharmacological products using simple synthetic routes and purification techniques. Of note, we have successfully transformed both gabapentin and pregabalin into their respective lactams using a new rapid, cheap, and scalable method. The gabapentin lactam possesses interesting biological properties, and the pregabalin lactam is classified as an expensive, fine chemical. These intermediates were then used to generate a collection of new chemical entities to be tested in future screening programs. The results described here are positive examples of how expired pharmaceuticals can be reutilized and upcycled. 

## Data Availability

The data are contained within the article and Supplementary Materials.

## Supplementary Materials

The following supporting information can be downloaded at https://www.mdpi.com/article/10.3390/molecules29204811/s1, Scheme S1: Mechanism of the reduction of 1 with NaBH_4_ and I_2_ to give 4; copies of the ^1^H and ^13^C NMR spectra of products; copies of the high-resolution mass spectra; HPLC conditions and HPLC spectral data; monitorization of the lactamization reaction of **1** by ^1^H NMR and LC-MS; copy of the ^1^H NMR spectrum of GABA lactamization reaction in DMF.

## References

[1] Calvo-Flores F.G., Isac-García J., Dobado J.A. (2018). Chapter 4: Overview of Pharmaceutical Products as Emerging Pollutants. Emerging Pollutants. Origin, Structure and Properties.

[2] Wydro U., Wołejko E., Luarasi L., Puto K., Taraseviciene Ž., Jabłonska-Trypuc A. (2024). A Review on Pharmaceuticals and Personal Care Products Residues in the Aquatic Environment and Possibilities for Their Remediation. Sustainability.

[3] Purdom C.E., Hardiman P.A., Bye V.J., Eno N.C., Tyler C.R., Sumpter J.P. (1994). Estrogenic effects of effluents from sewage treatment works. Chem. Ecol..

[4] Oaks J.L., Gilbert M., Virani M.Z., Watson R.T., Meteyer C.U., Rideout B.A., Shivaprasad H.L., Ahmed S., Chaudhry M.J.I., Arshad M. (2004). Diclofenac residues as the cause of vulture population decline in Pakistan. Nature.

[5] Smits A.P., Skelly D.K., Bolden S.R. (2014). Amphibian intersex in suburban landscapes. Ecosphere.

[6] Duarte D.J., Oldenkamp R., Ragas A.M.J. (2022). Human health risk assessment of pharmaceuticals in the European Vecht River. Integr. Environ. Assess Manag..

[7] Arnold K.E., Brown A.R., Ankley G.T., Sumpter J.P. (2014). Medicating the environment: Assessing risks of pharmaceuticals to wildlife and ecosystems. Phil. Trans. R. Soc. B.

[8] Commission Implementing Decision (EU) 2018/840 of 5 June 2018 Establishing a Watch List of Substances for Union-Wide Monitoring in the Field of Water Policy Pursuant to Directive 2008/105/EC of the European Parliament and of the Council and Repealing Commission Implementing Decision (EU) 2015/495. http://data.europa.eu/eli/dec_impl/2018/840/oj.

[9] Barcena H., Maziarz K. (2017). Chemical Upcycling of Expired Drugs: Synthesis of Guaifenesin Acetonide. J. Chem. Educ..

[10] Markus R. (2023). Heinrich, Recycling: Labor statt Müllcontainer. Nachr. Chem..

[11] Puzanova I., Lozynskyi A., Lesyk L., Hromovyk B., Lesyk R. (2019). Recycling of expired paracetamol-containing drugs as source of useful reagents for an organic synthesis. J. Appl. Pharm. Sci..

[12] Hou H., Li D., Liu X., Yao Y., Dai Z., Yu C. (2020). Recovery of Expired Lithium Carbonate Tablets for LiFePO_4_/C Cathode. Waste Biomass Valor..

[13] Kim Y., Hwang S.J., Kim D., Park J., Kim Y., Kim J., Bae M., Hong H., Park S., Park J. (2024). Direct Utilization of Expired Waste Acetaminophen as Organic Anode in Lithium-Ion Batteries. Adv. Mater. Interfaces.

[14] Hameed R.S., Aljuhani E., Felaly R., Munshi A. (2020). Effect of expired paracetamol–Zn^+2^ system and its synergistic effect towards iron dissolution inhibition and green inhibition performance. J. Adhes. Sci. Technol..

[15] Vaszilcsin N., Ordodi V., Borza A. (2012). Corrosion inhibitors from expired drugs. Int. J. Pharm..

[16] Sharma M., Kumar A., Chaudhary A. (2019). Reuse API (Active Pharmaceutical Ingredients) from Expired Dosage form. Int. J. Eng. Res. Technol..

[17] Alsaiari R.A., Kamel M.M., Mohamed M.M. (2024). Corrosion Inhibition of Expired Cefazolin Drug on Copper Metal in Dilute Hydrochloric Acid Solution: Practical and Theoretical Approaches. Molecules.

[18] Basha C., Babu K.R., Madhu M., Kumar Y.P., Gopinath C. (2015). Recycling of Drugs from Expired Drug products: Comprehensive Review. JGTPS.

[19] Jaber Y.S. Valorization of Pharmaceutical Waste by Recovery of Active Pharmaceutical Ingredients from Expired or Unused Finished Pharmaceutical Products (Fpps) with Thermodynamic Modeling. 32 Pages Posted: 26 February 2024. https://ssrn.com/abstract=4739545.

[20] Spanish Agency for Medicines and Health Products Drug Information Centre. https://cima.aemps.es/cima/publico/home.html.

[21] Ibers J.A. (2001). Gabapentin and gabapentin monohydrate. Acta Cryst..

[22] Reece H.A., Levendis D.C. (2008). Polymorphs of gabapentin. Acta Cryst..

[23] Venu N., Vishweshwar P., Ram T., Surya D., Apurba B. (2007). (*S*)-3-(Ammoniomethyl)-5-methylhexanoate (pregabalin). Acta Cryst..

[24] Braga D., Grepioni F., Maini L., Rubini K., Polito M., Brescello R., Cotarca L., Duarte M.T., André V., Piedade M.F.M. (2008). Polymorphic gabapentin: Thermal behaviour, reactivity and interconversion of forms in solution and solid-state. New J. Chem..

[25] Gorriti A.E., Istúriz N.A., González P.G., González J.F., Álvarez L.S., Parra J.G. (2023). Off-label use of gabapentinoid drugs: Is it necessary a deprescription strategy?. Gac. Sanit..

[26] FDA Warns about Serious Breathing Problems with Seizure and Nerve Pain Medicines Gabapentin (Neurontin, Gralise, Horizant) and Pregabalin (Lyrica, Lyrica CR). https://www.fda.gov/drugs/fda-drug-safety-podcasts/fda-warns-about-serious-breathing-problems-seizure-and-nerve-pain-medicines-gabapentin-neurontin.

[27] Chambers S.J., Coulthard G., Unsworth W.P., O’Brien P., Taylor R.J.K. (2016). From Heteroaromatic Acids and Imines to Azaspirocycles: Stereoselective Synthesis and 3D Shape Analysis. Chem. Eur. J..

[28] Lovering F., Bikker J., Humblet C. (2009). Escape from Flatland: Increasing Saturation as an Approach to Improving Clinical Success. J. Med. Chem..

[29] Bogolubsky A.V., Moroz Y.S., Mykhailiuk P.K., Pipko S.E., Konovets A.I., Sadkova I.V., Tolmachev T. (2014). Sulfonyl Fluorides as Alternative to Sulfonyl Chlorides in Parallel Synthesis of Aliphatic Sulfonamides. ACS Comb. Sci..

[30] Forte B., Norcross N., Jansen C., Baragana B., Gilbert I., Cleghorn L., Davis S., Walpole C. (2022). Preparation of Chromenone Carboxamides as Anti-Infective Agents. PCT International Application.

[31] Wang J., Cady S.D., Balannik V., Pinto L.H., DeGrado W.F., Hong M. (2009). Discovery of Spiro-Piperidine Inhibitors and Their Modulation of the Dynamics of the M2 Proton Channel from Influenza A Virus. J. Am. Chem. Soc..

[32] Badger A.M., Handler J.A., Genell C.A., Gore D.H.E., Polsky R., Webb L., Bugelski P.J. (1999). Atiprimod (SK&F 106615), a novel macrophage targeting agent, enhances alveolar macrophage candidacidal activity and is not immunosuppresive in candida-infected mice. Int. J. Immunopharmacol..

[33] Reilly S.W., Puentes L.N., Wilson K., Hsieh C.-J., Weng C.-C., Makvandi M., Mach R.H. (2018). Examination of Diazaspiro Cores as Piperazine Bioisosteres in the Olaparib Framework Shows Reduced DNA Damage and Cytotoxicity. J. Med. Chem..

[34] Yang S.M., Martinez N.J., Yasgar A., Danchik C., Johansson C., Wang Y., Baljinnyam B., Wang A.Q., Xu X., Shah P. (2018). Discovery of Orally Bioavailable, Quinoline-Based Aldehyde Dehydrogenase 1A1 (ALDH1A1) Inhibitors with Potent Cellular Activity. J. Med. Chem..

[35] Martyn D.C., Ramirez A.P., Beattie M.J., Cortese J.F., Patel V., Rush M.A., Woerpel K.A., Clardy J. (2008). Synthesis of spiro-1,2-dioxolanes and their activity against Plasmodium falciparum. Bioorg. Med. Chem. Lett..

[36] Ignacimuthu S.J., Baskar K., Duraipandiyan V. (2009). A Process for Preparing Compound Atalantiamide with Antifeedant, Larvicidal and Pupicidal Activities. India.

[37] Lagreze W.A., Muller-Velten R., Feuerstein T.J. (2001). The neuroprotective properties of gabapentin-lactam. Graefes Arch. Clin. Exp. Ophthalmol..

[38] Zucker B., Ludin D.E., Gerds T.A., Lücking C.H., Landwehrmeyer G.B., Feuerstein T.J. (2004). Gabapentin-lactam, but not gabapentin, reduces protein aggregates and improves motor performance in a transgenic mouse model of Huntington’s disease. Naunyn Schmiedeb. Arch. Pharmacol..

[39] Pielen A., Kirsch M., Hofmann H.D., Feuerstein T.J., Lagrèze W.A. (2004). Retinal ganglion cell survival is enhanced by gabapentin-lactam in vitro: Evidence for involvement of mitochondrial KATP channels. Graefes Arch. Clin. Exp. Ophthalmol..

[40] Henle F., Leemhuis J., Fischer C., Bock H.H., Lindemeyer K., Feuerstein T.J., Meyer D.K. (2006). Gabapentin-lactam induces dendritic filopodia and motility in cultured hippocampal neurons. J. Pharmacol. Exp. Ther..

[41] Oshima T., Duttenhoefer F., Xavier S., Nelson K., Sauerbier S. (2014). Can mesenchymal stem cells and novel gabapentin-lactam enhance maxillary bone formation?. J. Oral Maxillofac. Surg..

[42] Khan M.A. (2019). Spiro-Lactam NMDA Receptor Modulators and Uses Thereof. Patent.

[43] Agencia Española de Medicamentos y Productos Sanitarios Centro de Información Online de Medicamentos de la AEMPS. https://cima.aemps.es/cima/pdfs/es/ft/55917/55917_ft.pdf.

[44] World Health Organization Ageing and Health. https://www.who.int/es/news-room/fact-sheets/detail/ageing-and-health.

[45] Butler D.E., Greenman B.J. (1989). Gabapentin Monohydrate and a Process for Producing the Same. U.S. Patent.

[46] Lukàc M., Mrva M., Fischer-Fodor E., Lacko I., Bukovský M., Miklašova N., Ondriska F., Devinsky F. (2009). Synthesis and biological activity of dialkylphosphocholines. Bioorg. Med. Chem. Lett..

[47] McKennon M.J., Meyers A.I., Darauz K., Schwarm M. (1993). A convenient reduction of amino acids and their derivatives. J. Org. Chem..

[48] Sircar S.S.G. (1928). The Preparation of the Substituted Butyrolactams. J. Indian Chem. Soc..

[49] Geibel W., Hartenstein J., Herrmann W., Witzke J. (1992). Process for the Preparation of 1-Aminomethyl-Cyclohexaneacetic Acid. U.S. Patent.

[50] Steiner K., Herrmann W., Crone G., Combs C.S. (1991). Process for the Preparation of Cyclic Amino Acids and Intermediates Useful in the Process. U.S. Patent.

[51] Yerande S.G., Thakur R.M., Sharma S.S., Gangopadhyay A.K., Rupp H., Kubavat H.T., Nagarajan K., Selvan A., Nunna R., Jalajakshi V.I. (2013). A Process for the Preparation of Gabapentin. PCT International Application.

[52] Padgaonkar S.G., Thennati R. (2001). Process Suitable for Industrial Scale Production of Gabapentin. Indian Patent.

[53] Nagarajan K., Sivaramakrishnan H., Arulselvan M. (2009). Process for the Preparation of Gabalactam. U.S. Patent.

[54] Bryans J.S., Chessum N.E.A., Huther N., Parsons A.F., Ghelfi F. (2003). Metal-catalysed radical cyclisations leading to N-heterocycles: New approaches to gabapentin and pulchellalactam. Tetrahedron.

[55] Cagnoli R., Ghelfi F., Pagnoni U.M., Parsons A.F., Schenetti L. (2003). Hydro-de-halogenation and consecutive deprotection of chlorinated *N*-amido-pyrrolidin-2-ones with Raney-Ni: An effective approach to gabapentin. Tetrahedron.

[56] Chen Z., Chen Z., Jiang Y., Hu W. (2003). A Concise Synthesis of Gabapentin via Intramolecular C-CH Insertion Reaction. Synlett..

[57] Chen Z., Chen Z., Jiang Y., Hu W. (2005). The synthesis of baclofen and GABOB via Rh(II) catalyzed intramolecular C–H insertion of α-diazoacetamides. Tetrahedron.

[58] Katuri J.V.P., Ekkundi V.S., Kuppuswamy N. (2016). A Simple and Expedient Procedure for the Preparation of Gabapentin Lactam (2-aza-spiro[4,5]decan-3-one). Org. Process Res. Dev..

[59] Hadidi S., Shiri F., Norouzibazaz M. (2020). Theoretical Mechanistic Insight into the Gabapentin Lactamization by an Intramolecular Attack: Degradation Model and Stabilization Factors. J. Pharm. Biomed. Anal..

[60] Dempah K.E., Barich D.H., Kaushal A.M., Zong Z., Desai S.D., Suryanarayanan R., Kirsch L., Munson E.J. (2013). Investigating Gabapentin Polymorphism Using Solid-State Nmr Spectroscopy. AAPS PharmSciTech..

[61] Lin S., Hsu C., Ke W.-T. (2010). Solid-State Transformation of Different Gabapentin Polymorphs upon Milling and Co-Milling. Int. J. Pharm..

[62] Zong Z., Desai S.D., Kaushal A.M., Barich D.H., Huang H., Munson E.J., Suryanarayanan R., Kirsch L.E. (2011). The Stabilizing Effect of Moisture on the Solid-State Degradation of Gabapentin. AAPS PharmSciTech..

[63] Zong Z., Qiu J., Tinmanee R., Kirsch L.E. (2012). Kinetic Model for Solid-State Degradation of Gabapentin. J. Pharm. Sci..

[64] Kearney A.S., Mehta S.C., Radebaugh G.W. (1992). The effect of cyclodextrins on the rate of intramolecular lactamization of gabapentin in aqueous solution. Int. J. Pharm..

[65] Camilleri P., Ellul R., Kirby A.J., Mujahid T.G. (1979). The Spontaneous Formation of Amides. The Mechanism of Lactam Formation from 3-(2-Aminophenyl)propionic Acid. J. Chem. Soc. Perkin Trans. 2.

[66] Abdallah J.M., Moodie R.B. (1983). Kinetics and Equilibria of Ring Closure through an Amide Linkage. Part 2. 1 -Aryl-2-pyrrolidones. J. Chem. Soc. Perkin Trans. 2.

[67] Reyes-Batlles M., Blanco M.F., López-Arencibia A., Lorenzo-Morales J., McNaughton-Smith G., Piñero J.E., Abad-Grillo T. (2020). Identification of *N*-acyl quinolin-2(1*H*)-ones as new selective agents against clinical isolates of Acanthamoeba keratitis. Bioorg. Chem..

