# Supplementary material for: Chemical Upcycling of Expired Pharmaceuticals as a Source of Value-Added Chemicals for Organic Synthesis and Medicinal Chemistry
Source: Molecules. 2024 Oct 11;29(20):4811. doi: 10.3390/molecules29204811 (PMC11510324; doi:10.3390/molecules29204811)

Supplementary Materials

Chemical Upcycling of Expired Pharmaceuticals as a Source of Value-added Chemicals for Organic Synthesis and Medicinal Chemistry

Teresa Abad-Grillo[1,*] and Grant McNaughton-Smith[2]

[1] Departamento de Química Orgánica, Universidad de La Laguna, Avenida Astrofísico Francisco Sánchez, 2, 38206 La Laguna, Tenerife, Spain; tereabad@ull.edu.es

[2] Centro Atlántico del Medicamento S.A (CEAMED S.A.), PCTT, 38200 La Laguna, Tenerife, Spain; gmcsmith@ceamedsa.com

*Correspondence: tereabad@ull.edu.es

Contents Page

1. Scheme S1. Mechanism of the reduction of **1** with $NaBH_4$ and I_2 to give **4** S2

2. Copies of the ^{1}H and ^{13}C NMR spectra of products S3

3. Copies of the High Resolution Mass Spectra of products S28

4. HPLC conditions and HPLC spectral data S42

5. Monitoring of the lactamization reaction of **1** to **5** S50
   (a) ^{1}H NMR (CD_3OD) S50
   (b) LC-MS conditions S53
   (c) LC-MS total ion count monitoring S55
   (d) Photos S57

6. Copy of the ^{1}H NMR spectrum of GABA lactamization reaction in DMF S58

According to the literature [41,42], the reducing agent (BH_3-THF complex) is generated in situ. Therefore, **4** as a byproduct would be explained through the intermediate complex indicated in scheme S1.

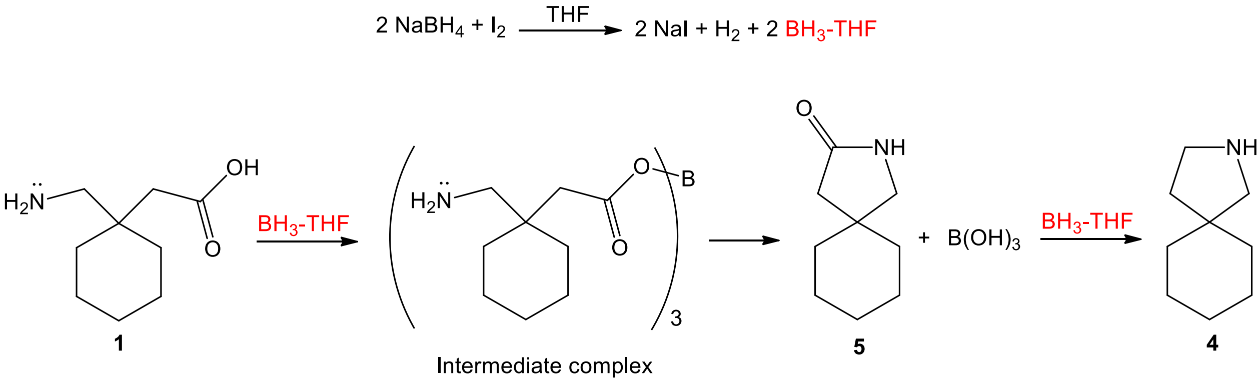

Scheme S1. Mechanism of the reduction of **1** with $NaBH_4$ and I_2 to give **4**

Copies of ^{1}H and ^{13}C NMR spectra of products.

^{1}H NMR spectrum (500 MHz, CD_3OD) of compound **1**:

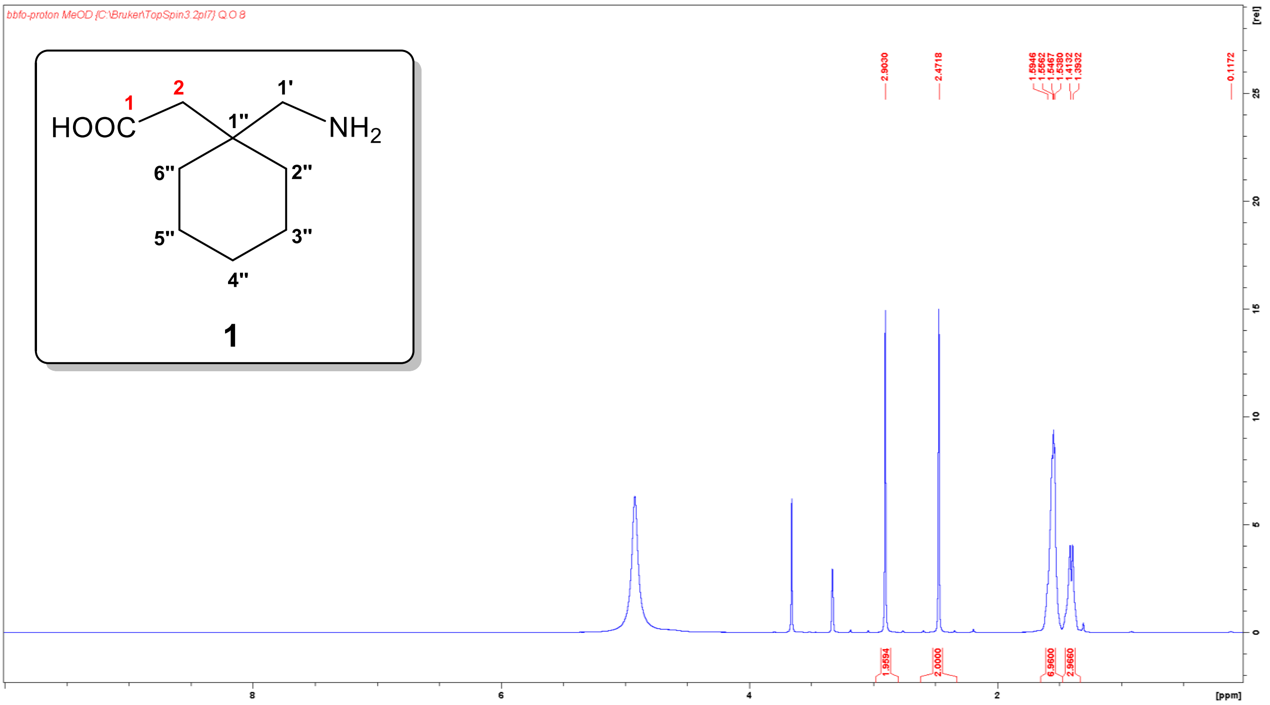

The signal at 3.65 ppm corresponds to an impurity of less than 1% of macrogol 4000 (polyethylene glycol), one of the excipients.

^{13}C NMR spectrum (125 MHz, CD_3OD) of compound **1**:

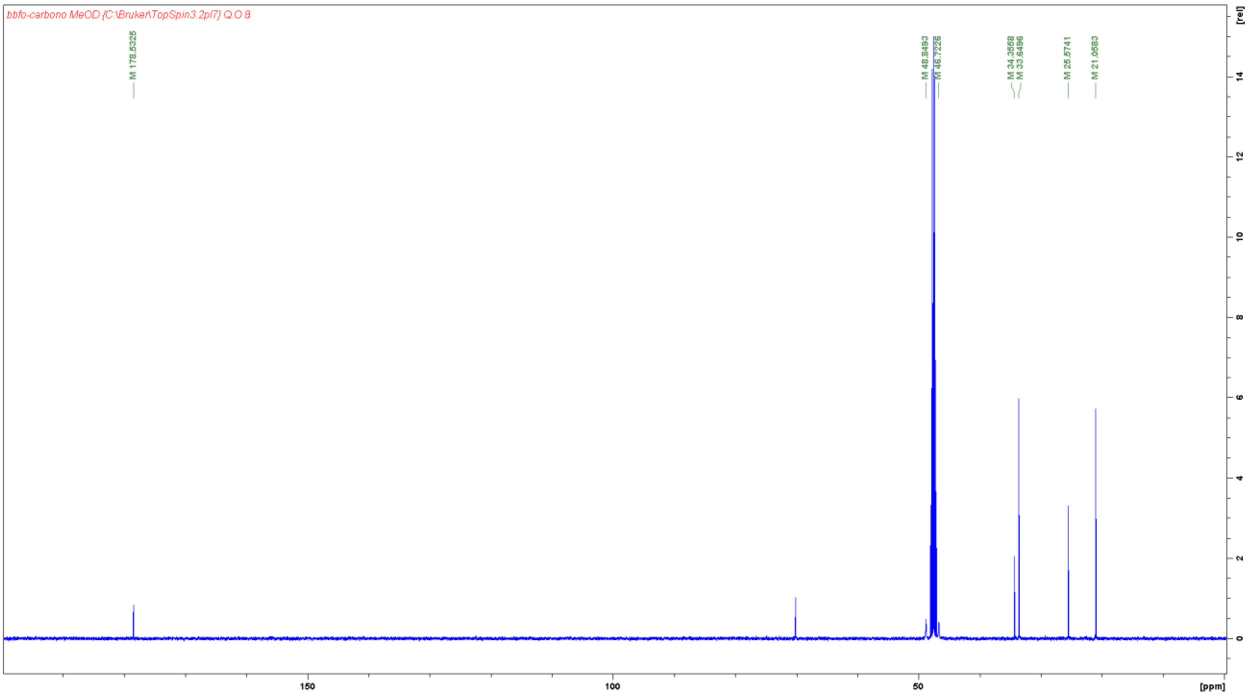

The signal at 70.17 ppm corresponds to an impurity of less than 1% of macrogol 4000 (polyethylene glycol), one of the excipients.

HSQCed spectrum (500 MHz, $CDCl_3$) of compound **1**:

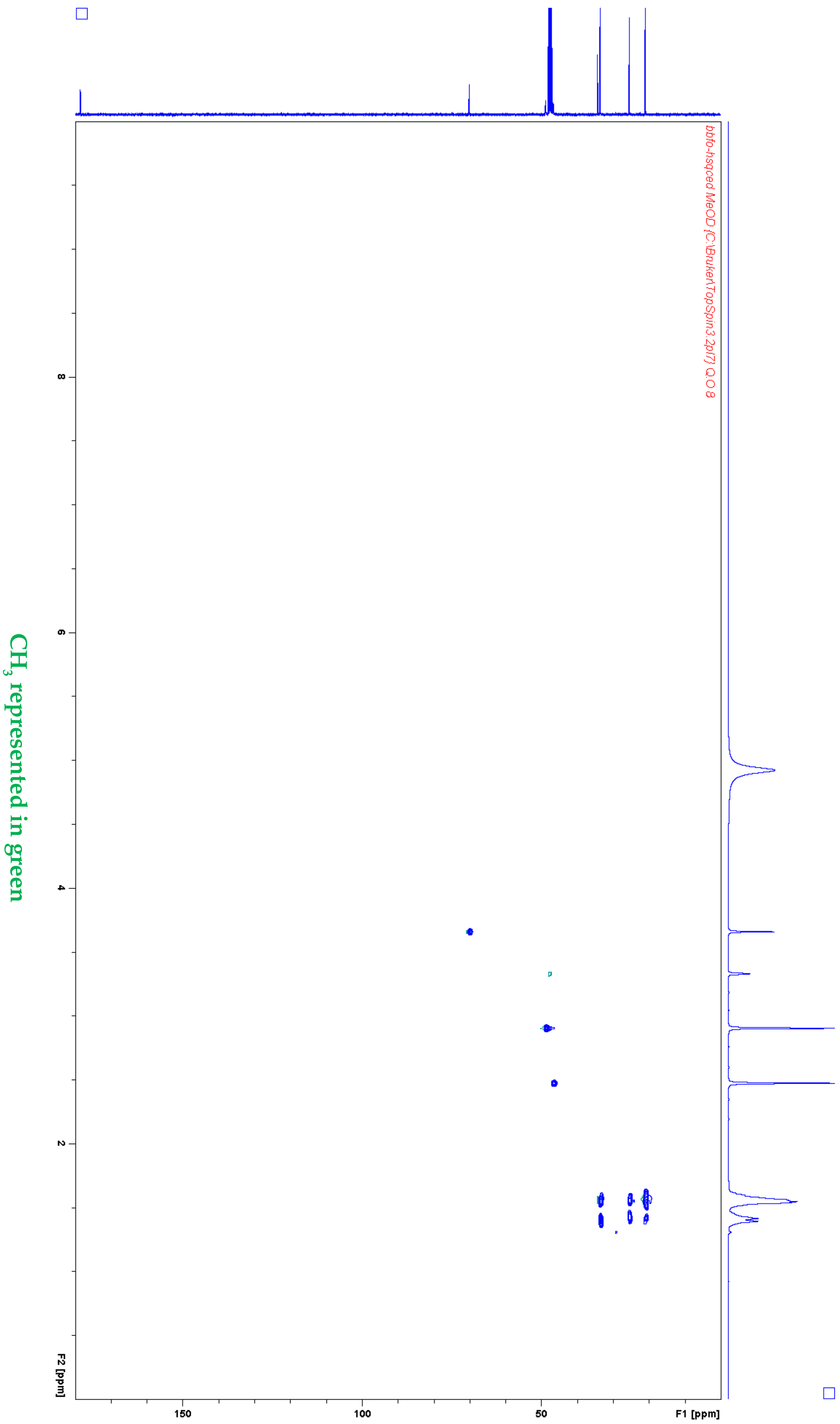

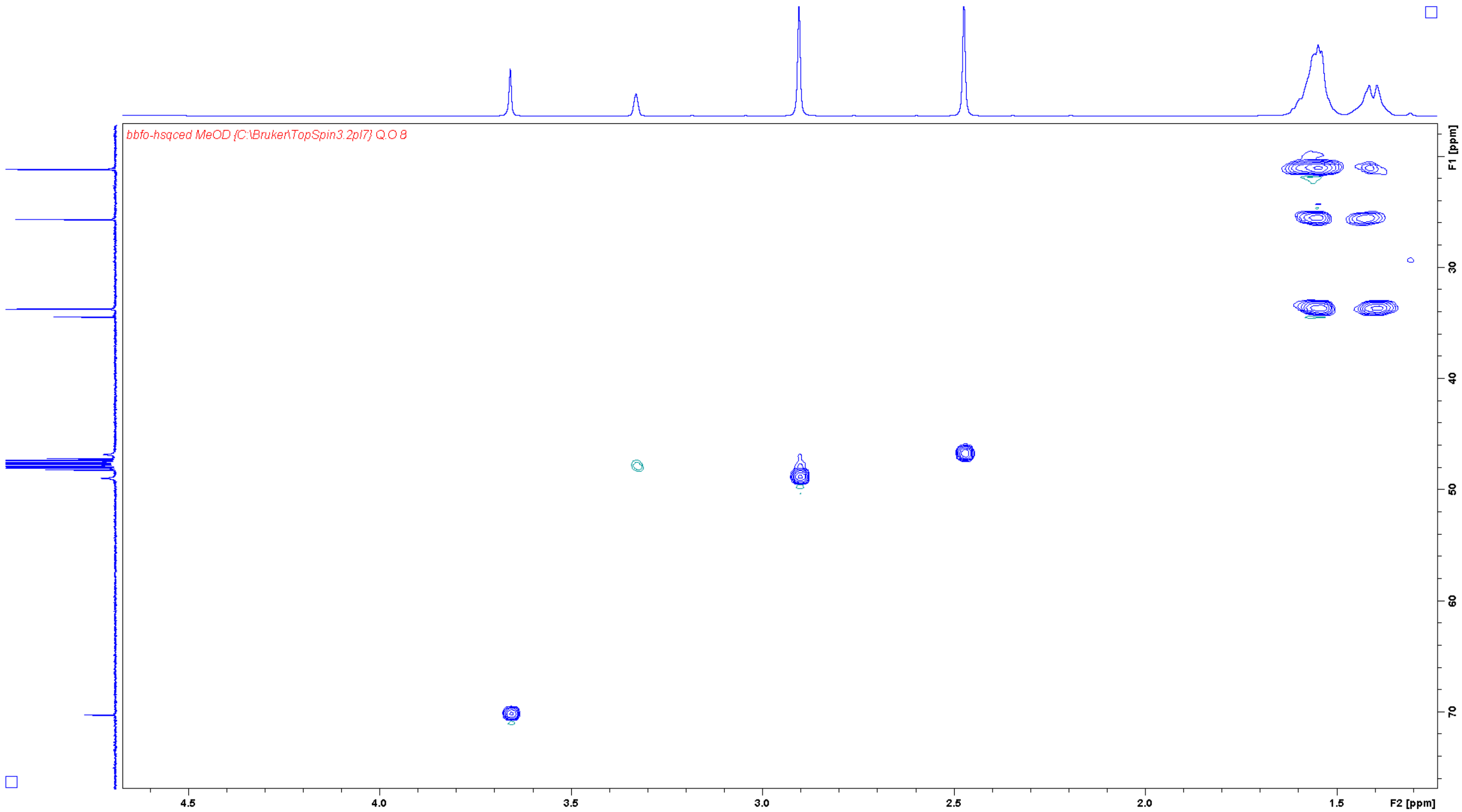
bbfo-hsqced MeOD {C:\Bruker\TopSpin3.2pl7} Q.O 8
F1 [ppm]
30
40
50
60
70
4.5
4.0
3.5
3.0
2.5
2.0
1.5
F2 [ppm]

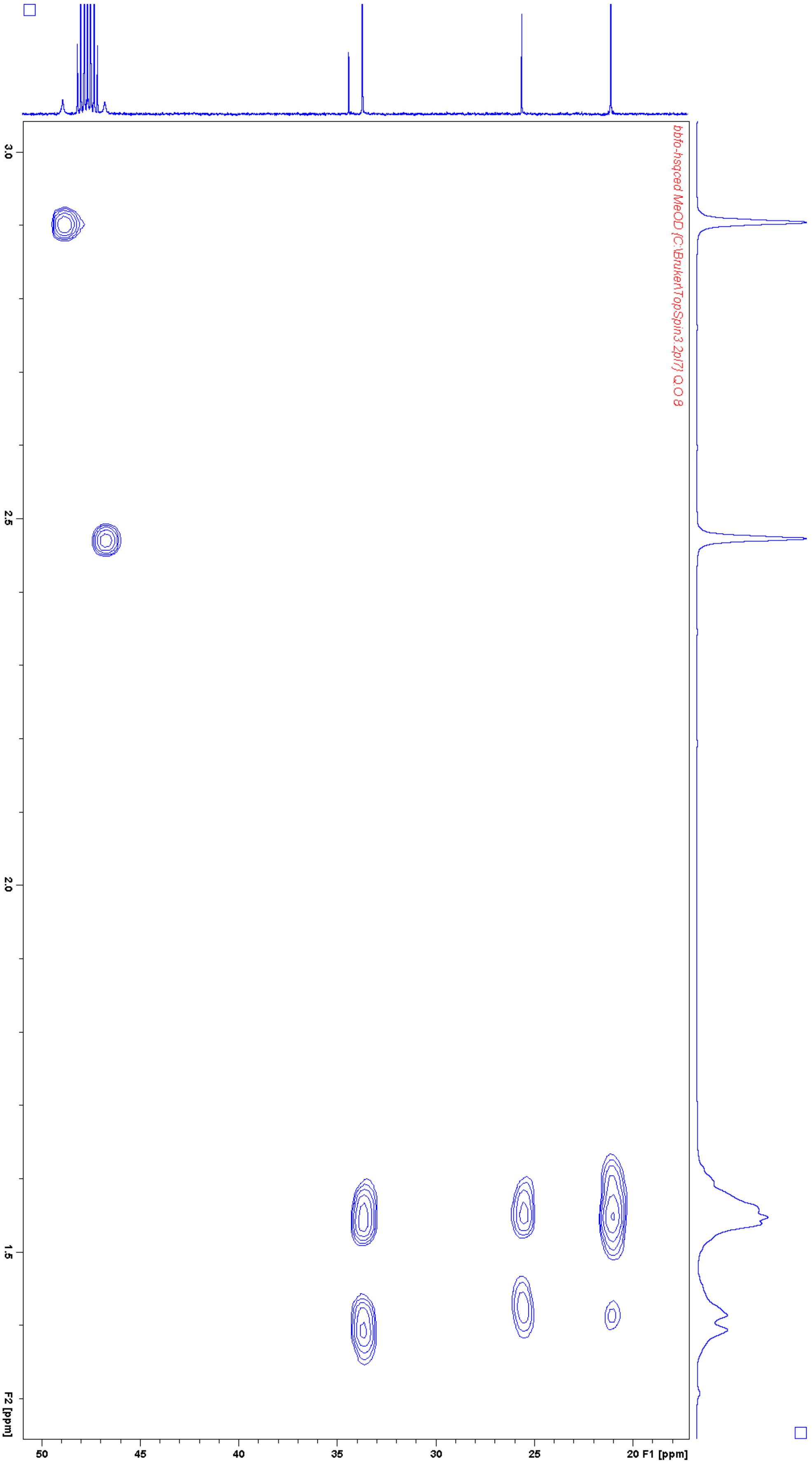
bbfo-hsqced MeOD {C:\Bruker\TopSpin3.2pl7} Q.O.8
3.0
2.5
2.0
1.5
F2 [ppm]
50
45
40
35
30
25
20 F1 [ppm]

^1H NMR spectrum (500 MHz CD_3OD) of compound **2** with impurities (mixture of excipients):

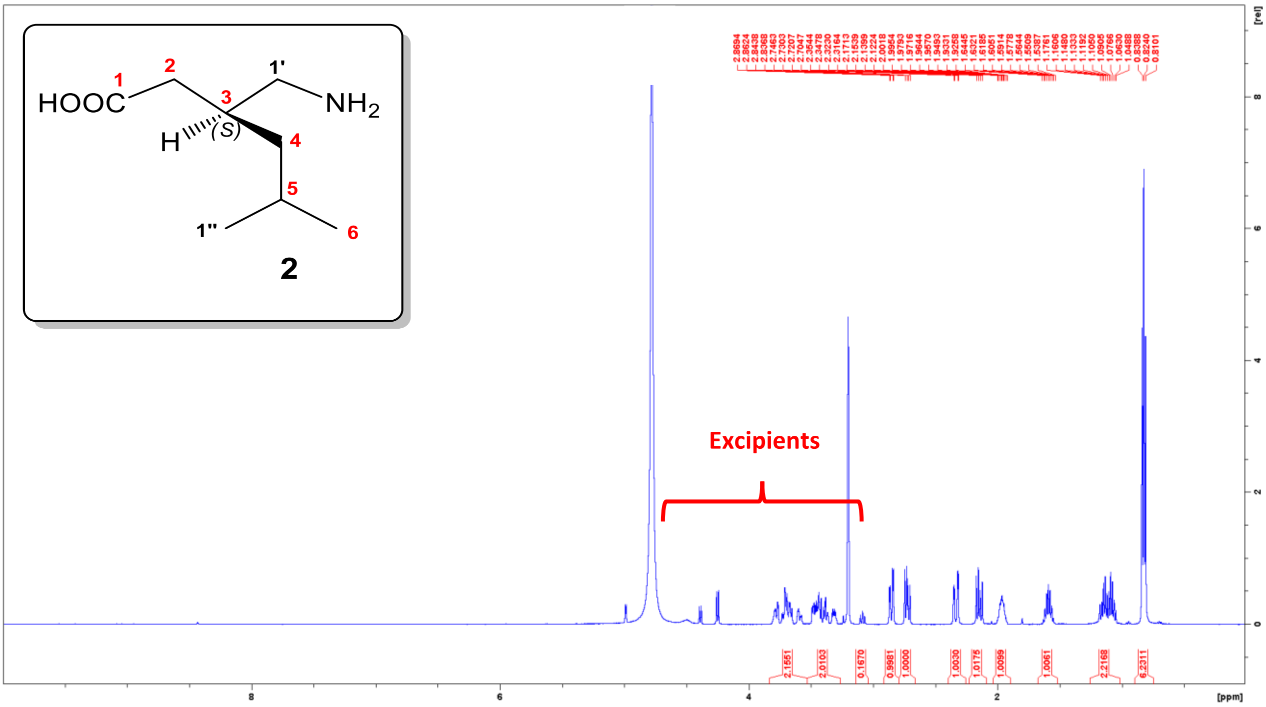

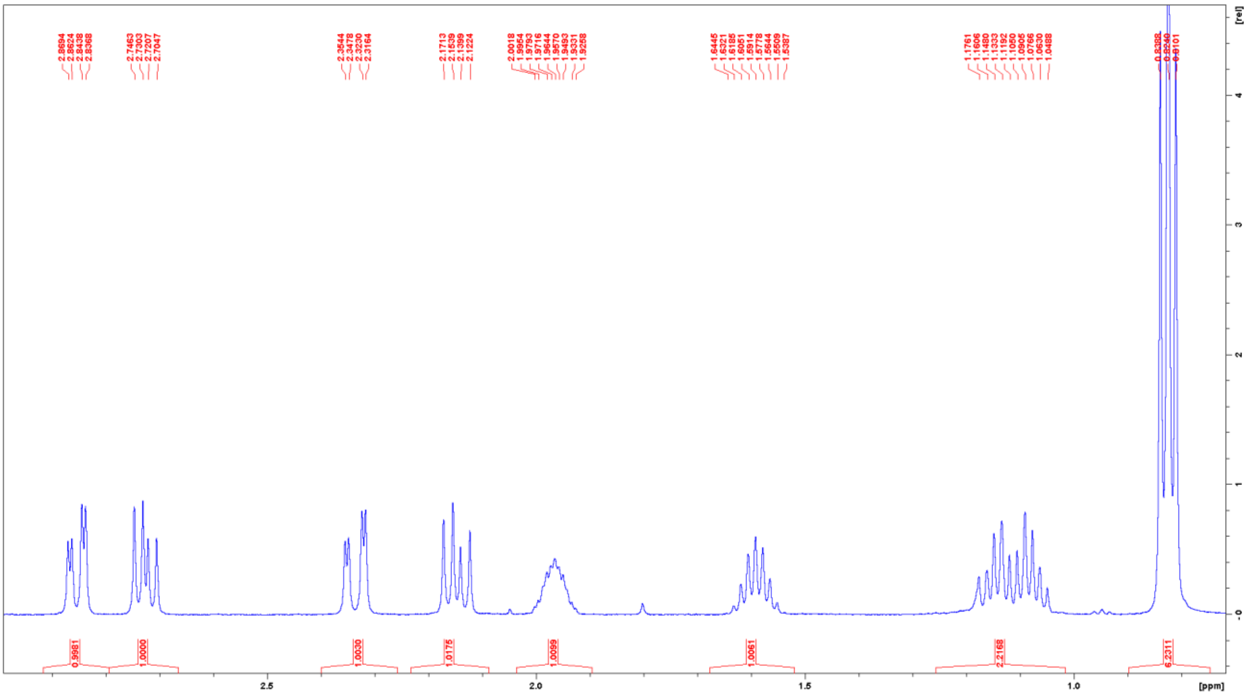

^{1}H NMR spectrum (500 MHz, $CDCl_3$) of compound **3**:

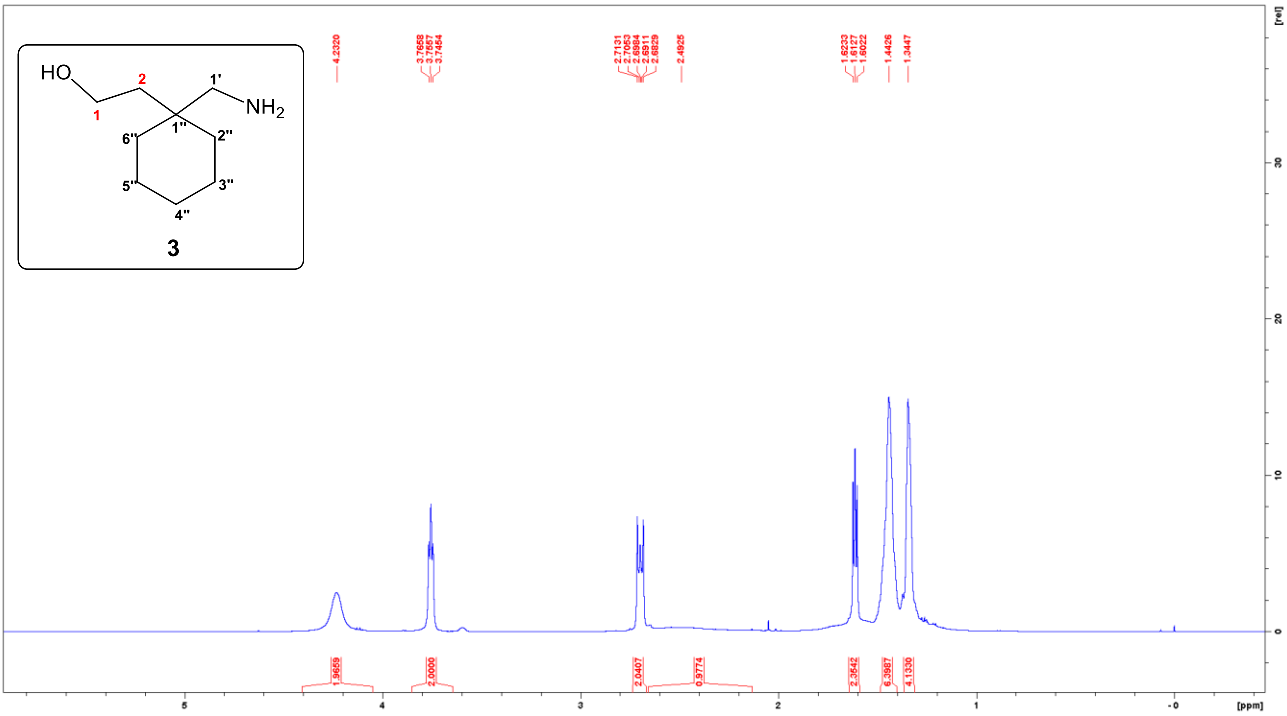

^{13}C NMR spectrum (125 MHz, $CDCl_3$) of compound **3**:

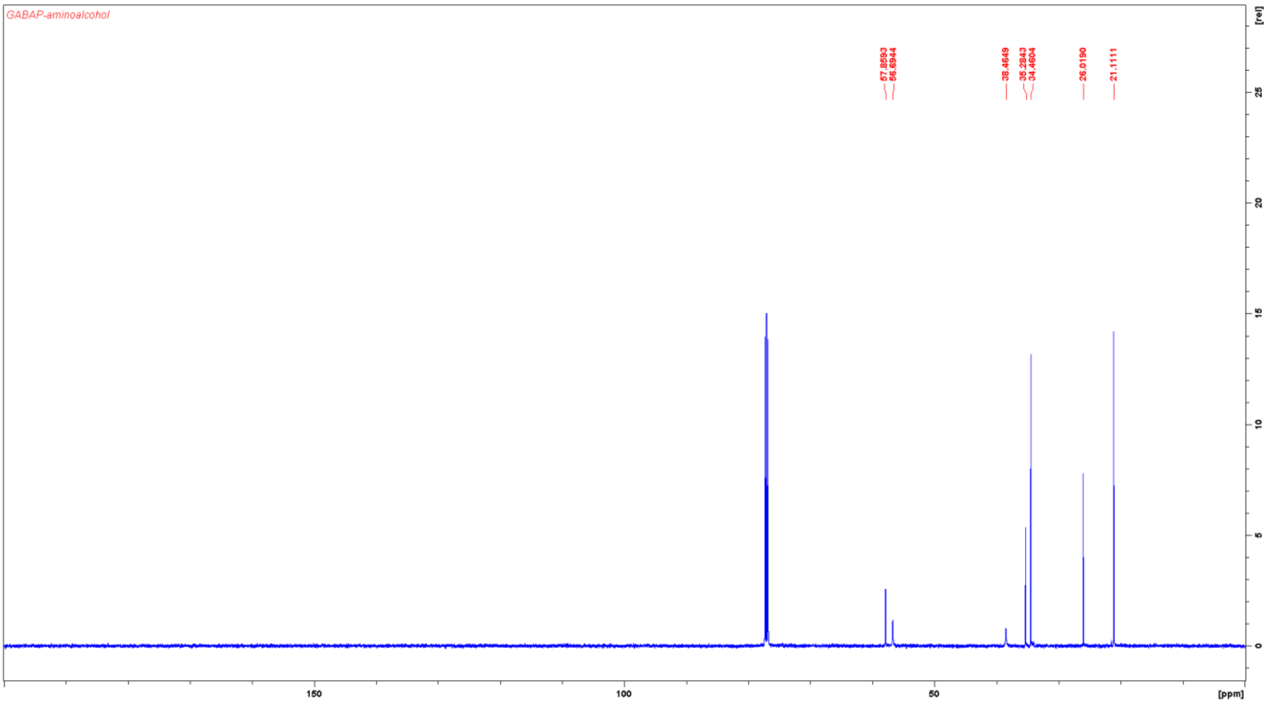

^{1}H NMR spectrum (500 MHz, $CDCl_3$) of compound **4**:

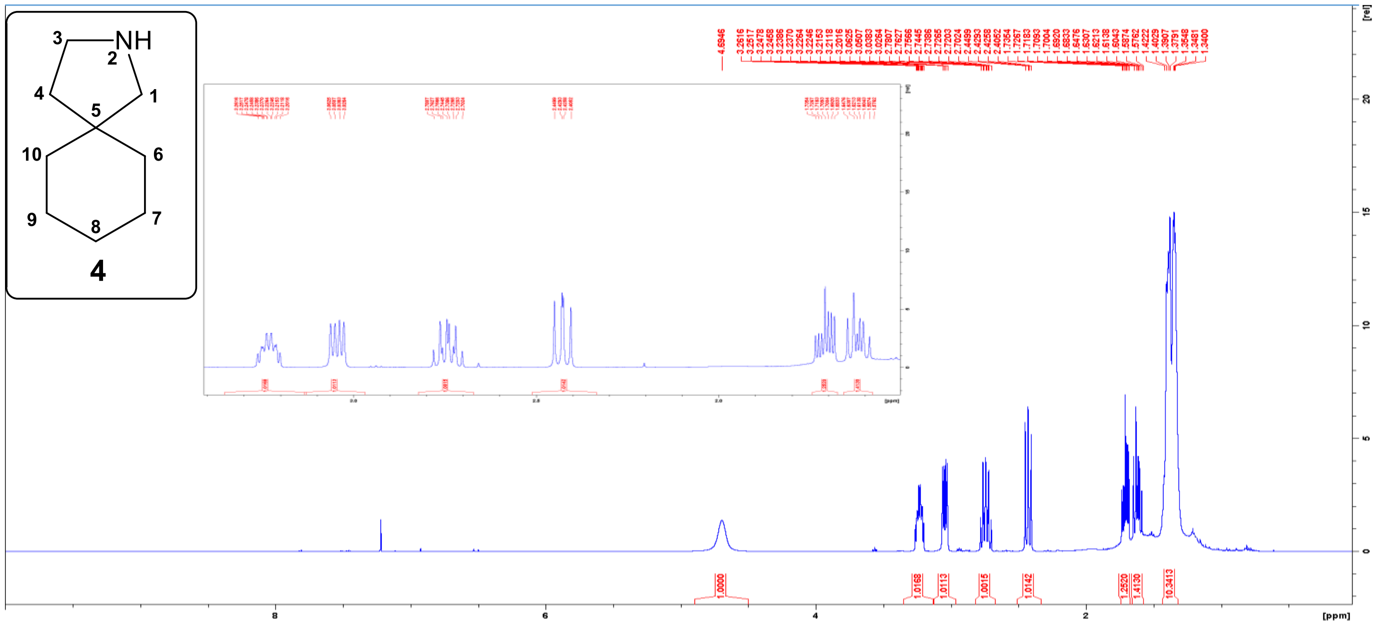

^{13}C NMR spectrum (125 MHz, $CDCl_3$) of compound **4**:

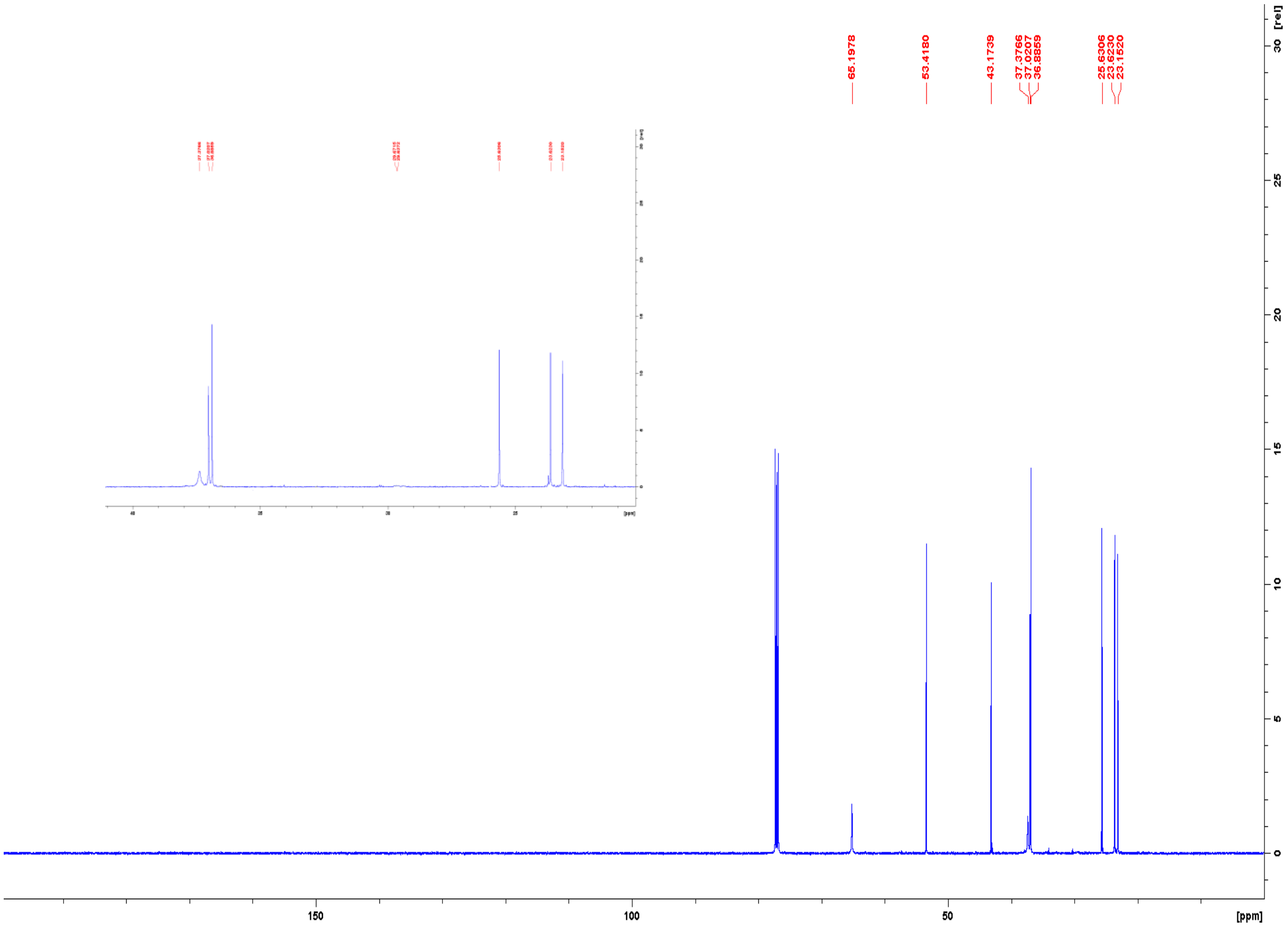

^{1}H NMR spectrum (500 MHz, $CDCl_3$) of compound **5**:

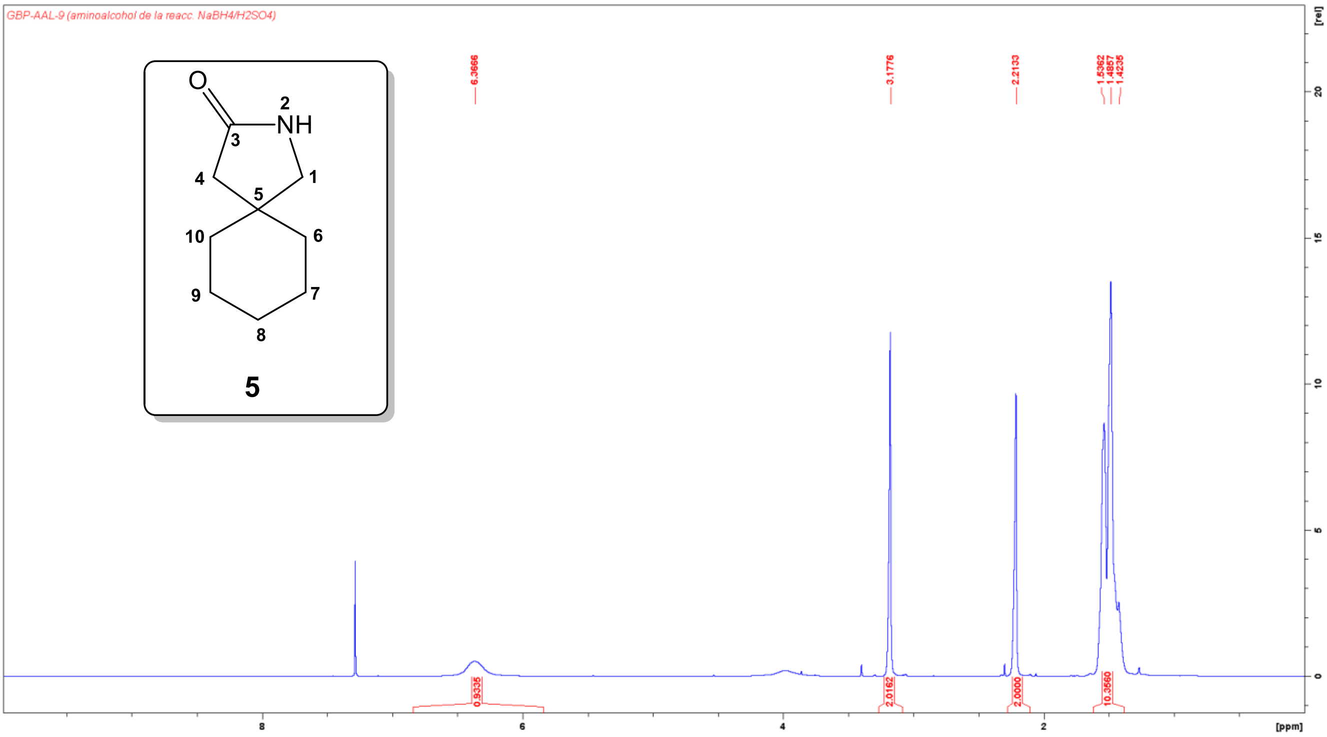

^{13}C NMR spectrum (150 MHz, $CDCl_3$) of compound **5**:

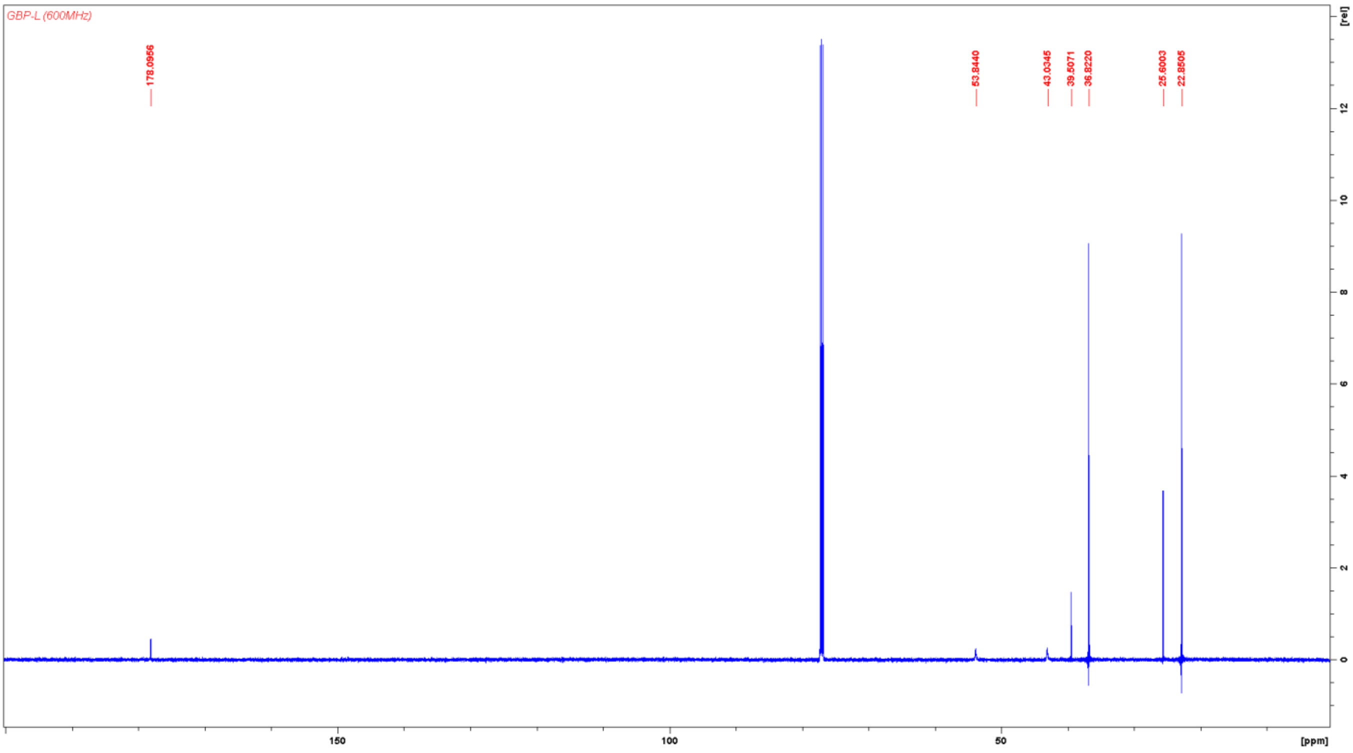

^{1}H NMR spectrum (500 MHz, CD_3OD) of compound **5**:

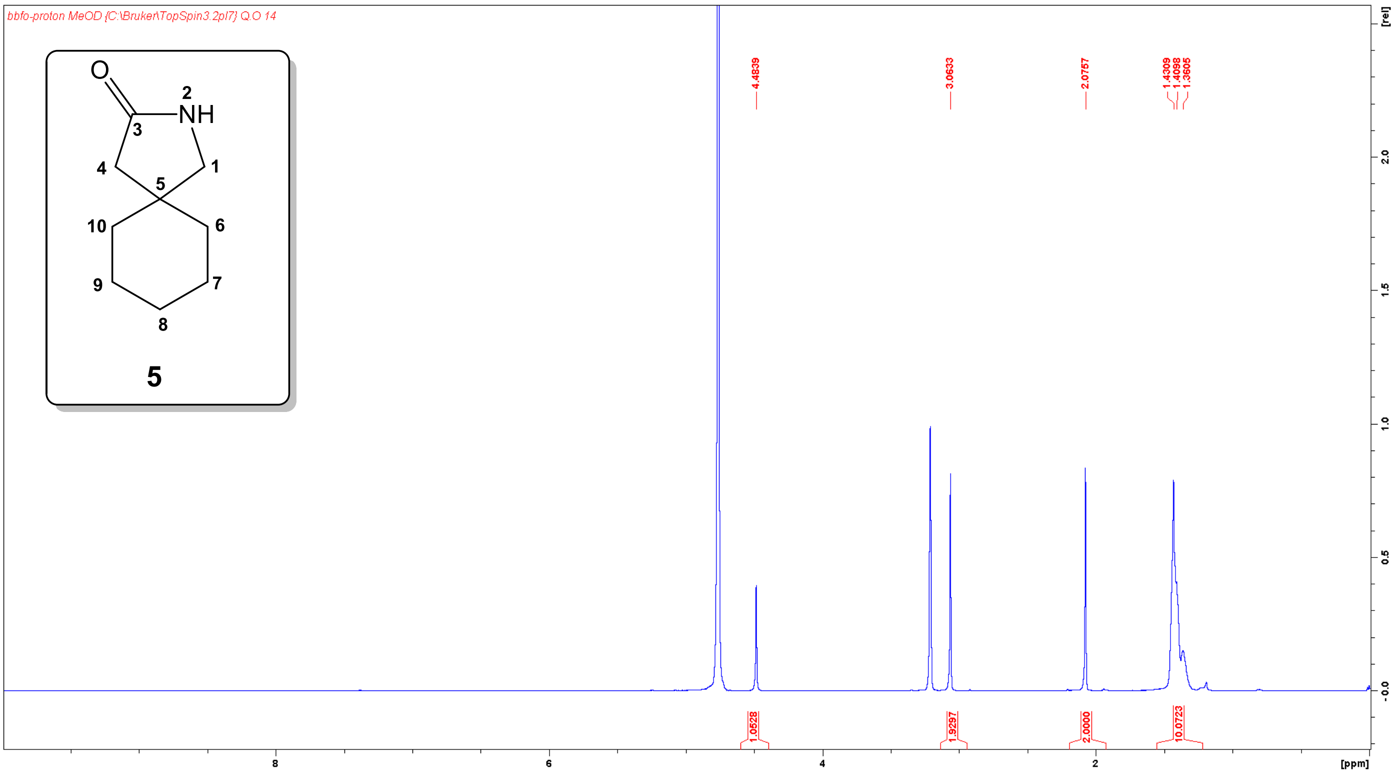

^{1}H NMR spectrum (500 MHz, $CDCl_3$) of compound **5a**:

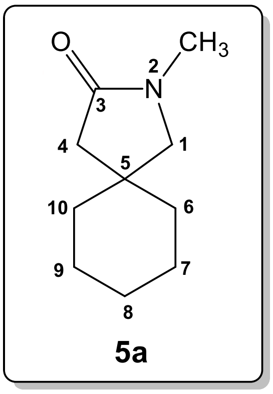

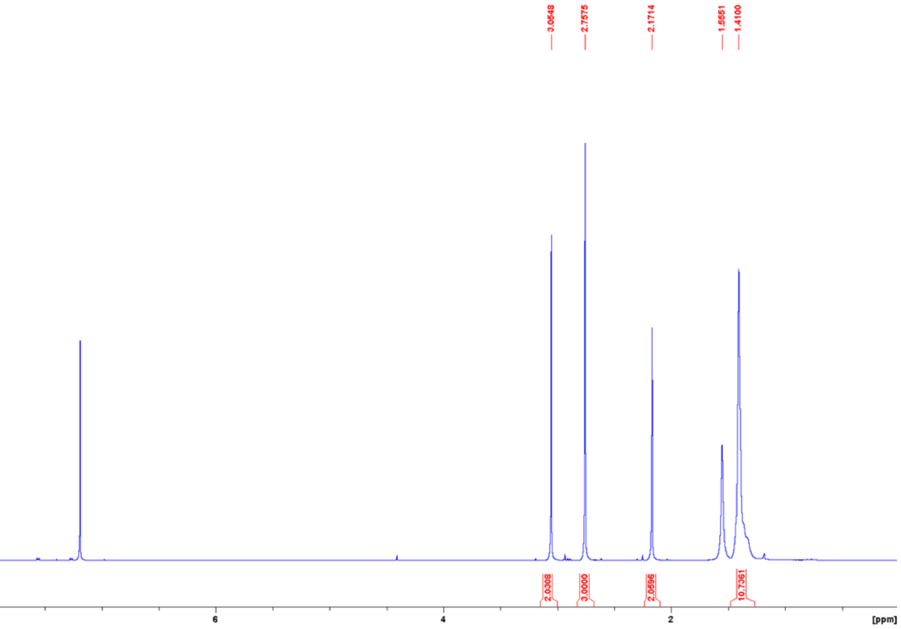

^{13}C NMR spectrum (125 MHz, $CDCl_3$) of compound **5a**:

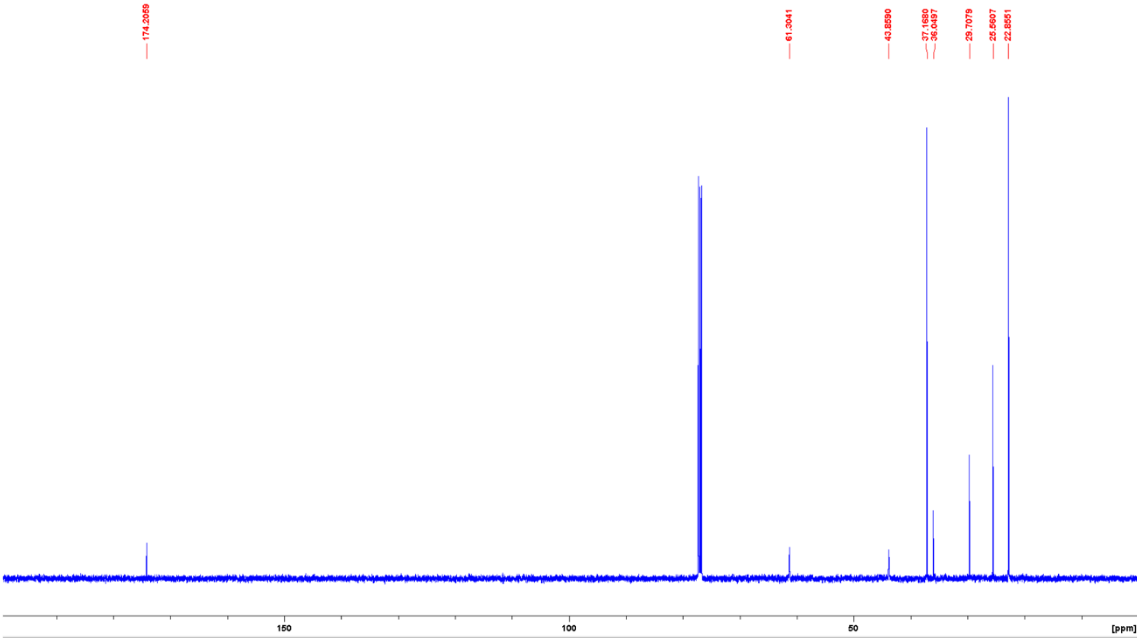

^1H NMR spectrum (500 MHz, $CDCl_3$) of compound **5b**:

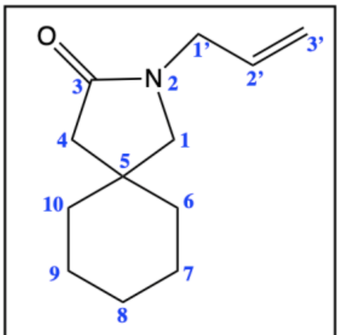

5b **1c**

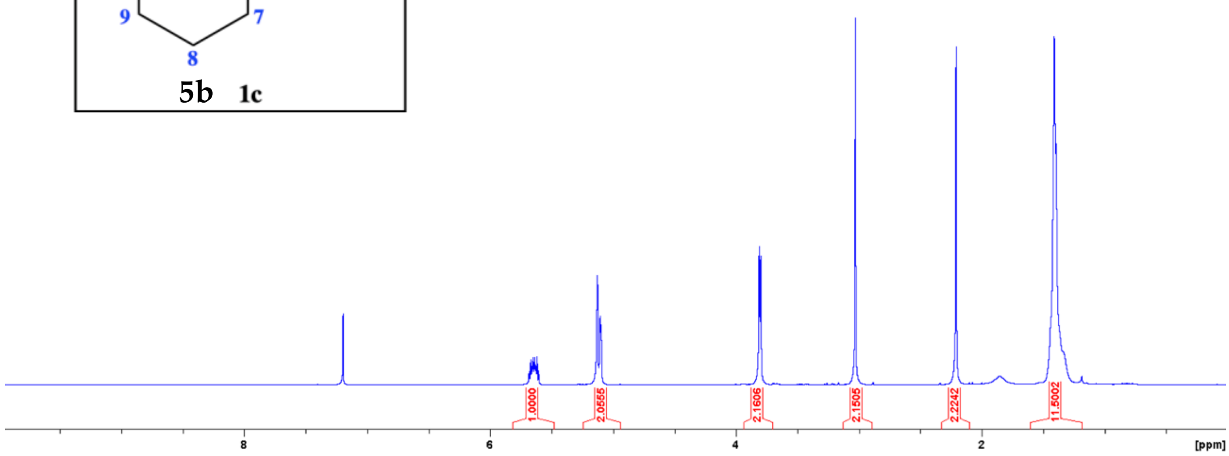

^{13}C NMR spectrum (125 MHz, $CDCl_3$) of compound **5b**:

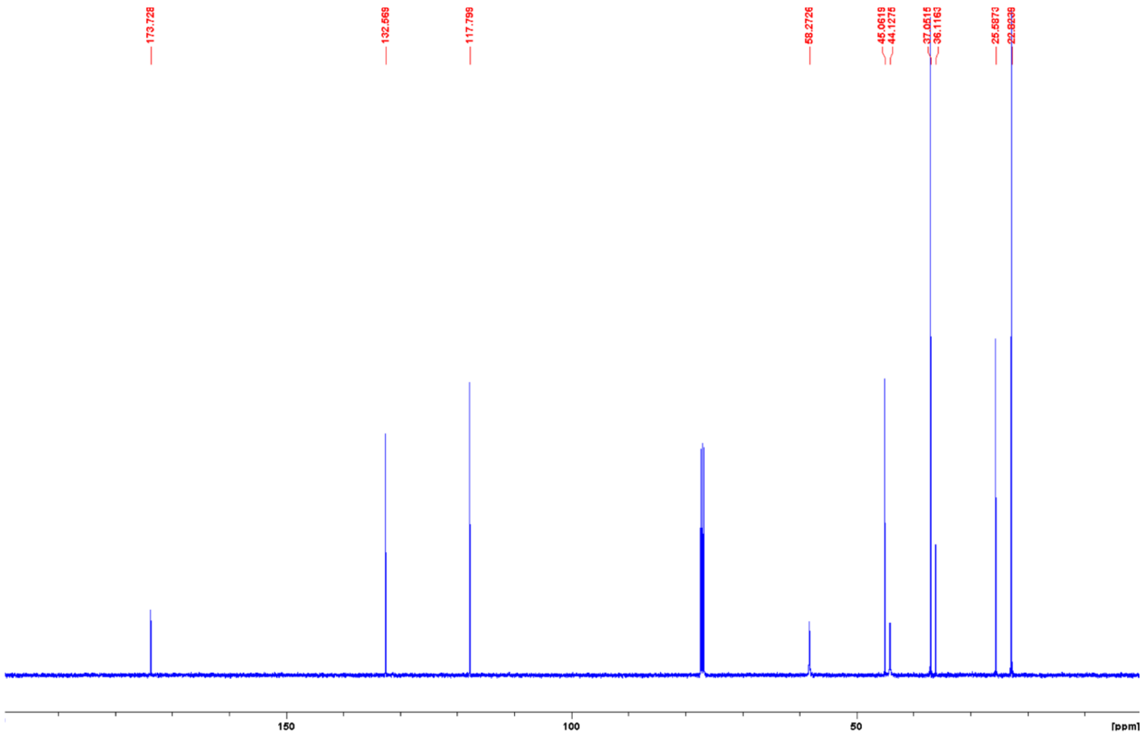

^{1}H NMR spectrum (500 MHz, $CDCl_3$) of compound **5c**:

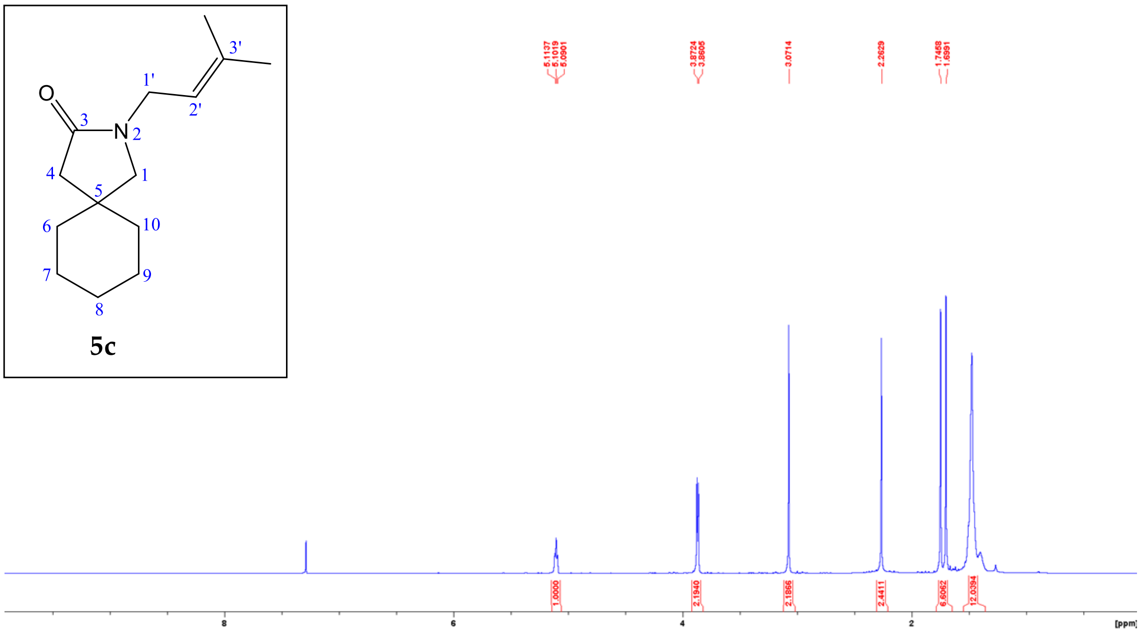

^{13}C NMR spectrum (125 MHz, $CDCl_3$) of compound **5c**:

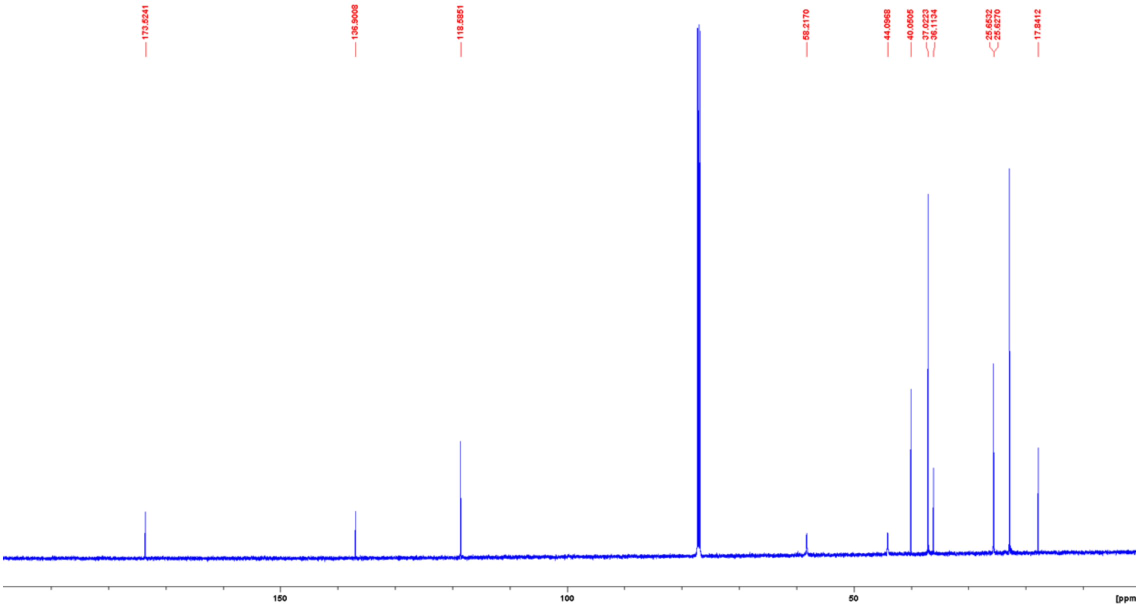

^{1}H NMR spectrum (500 MHz, $CDCl_3$) of compound **5d**:

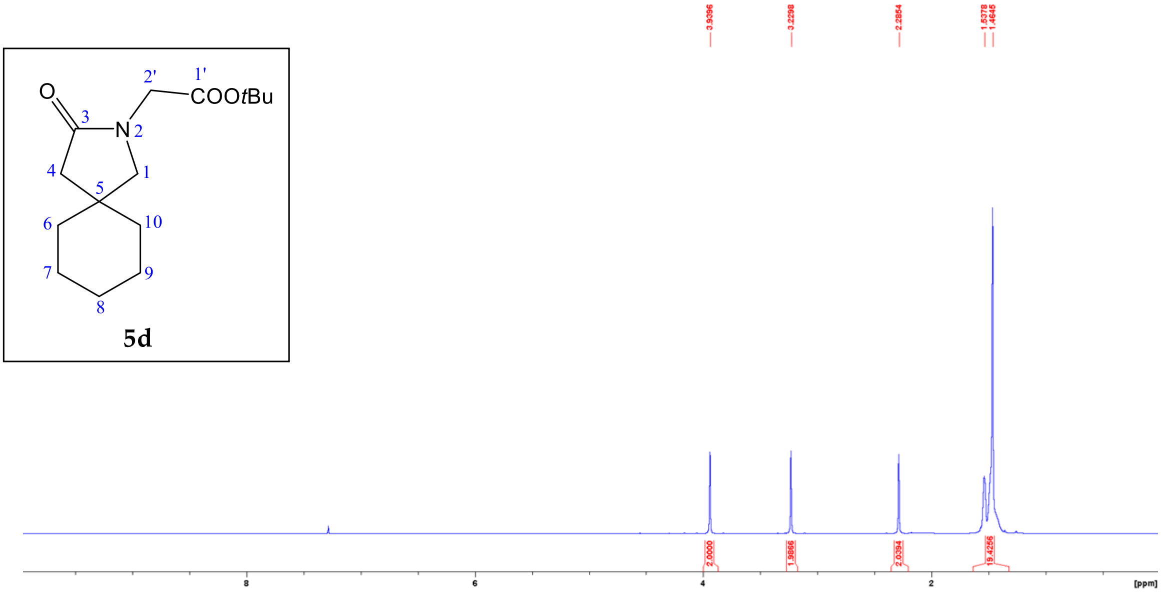

^{13}C NMR spectrum (125 MHz, $CDCl_3$) of compound **5d**:

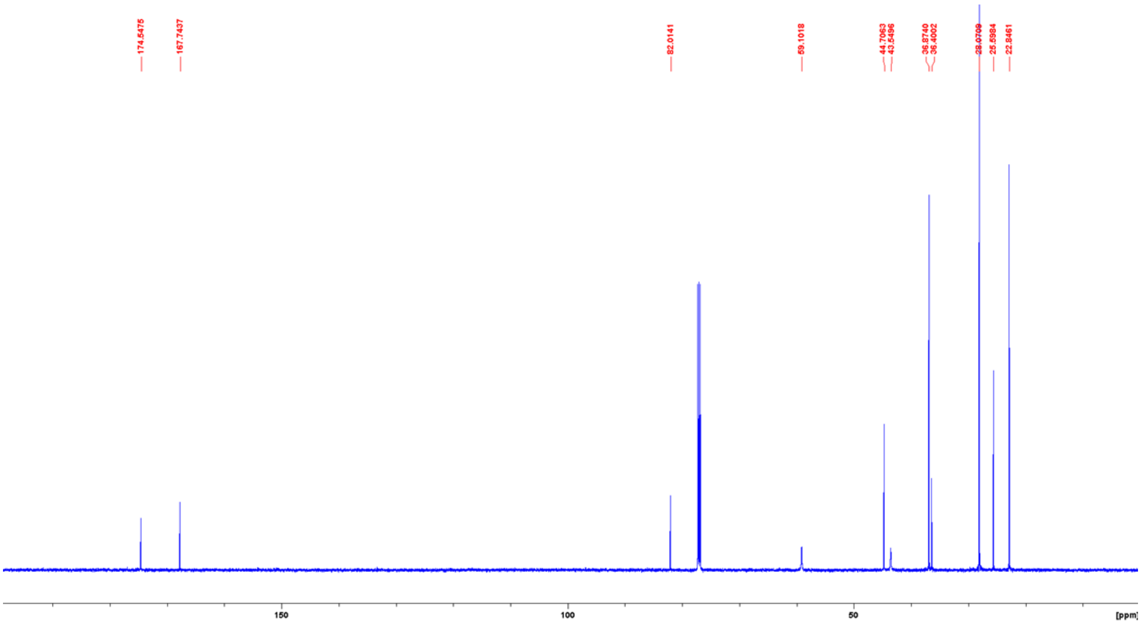

^{1}H NMR spectrum (500 MHz, $CDCl_3$) of compound **5e**:

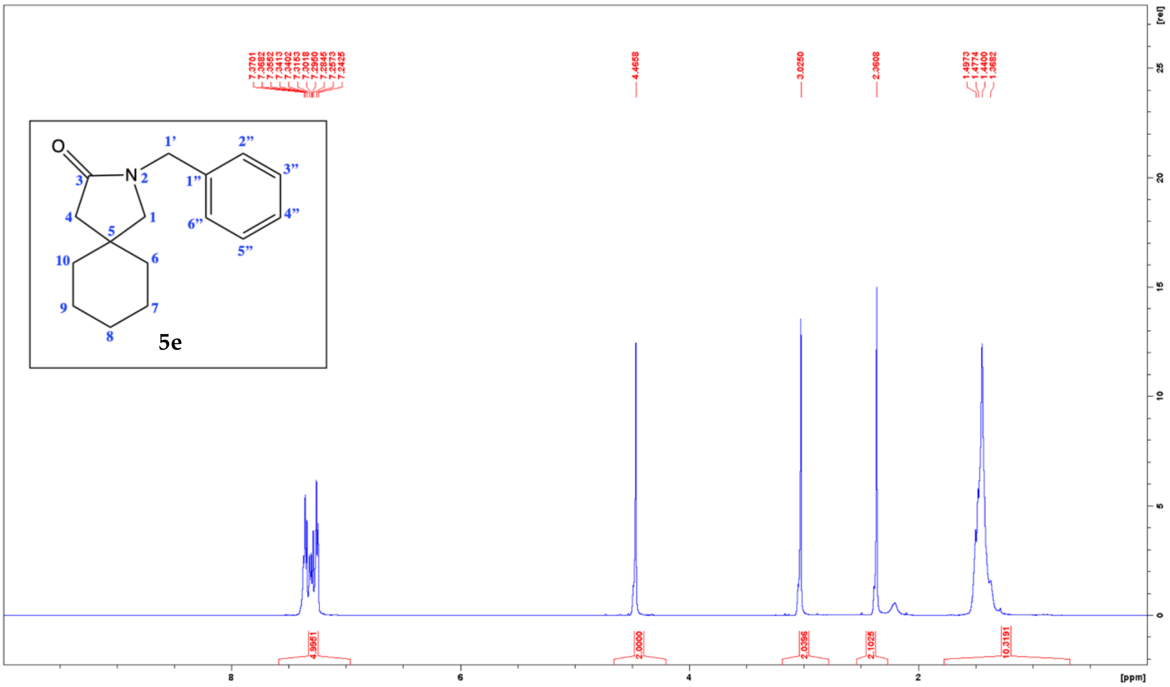

^{13}C NMR spectrum (125 MHz, $CDCl_3$) of compound **5e**:

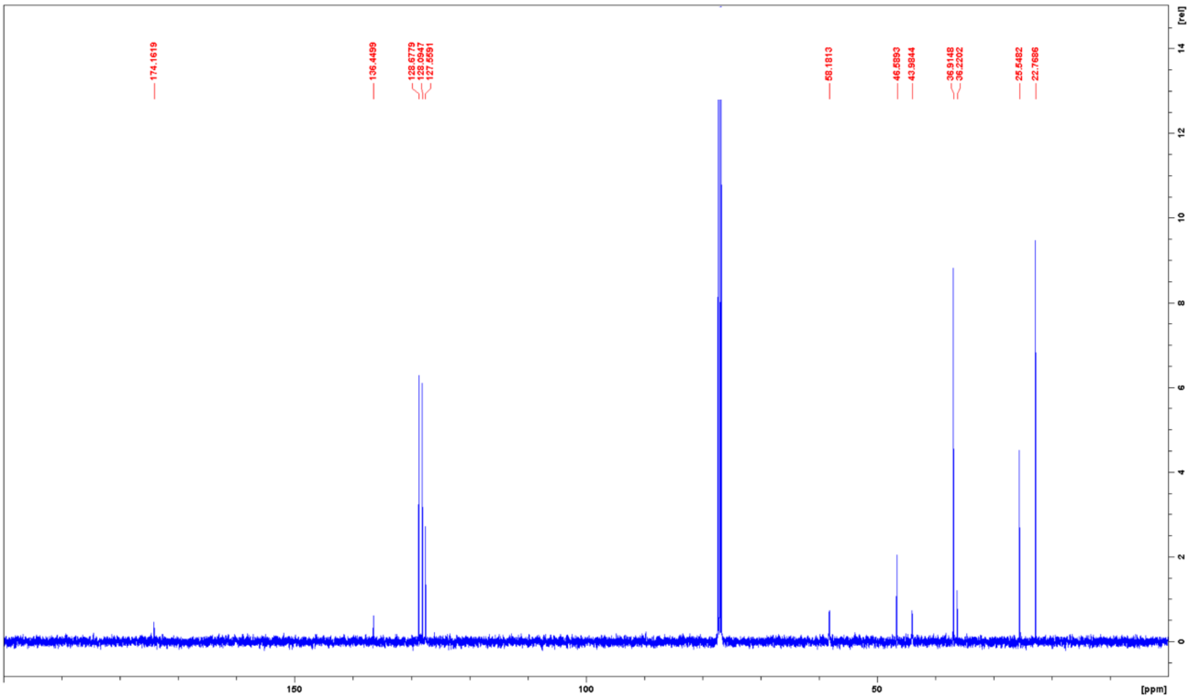

^{1}H NMR spectrum (500 MHz, $CDCl_3$) of compound **5f**:

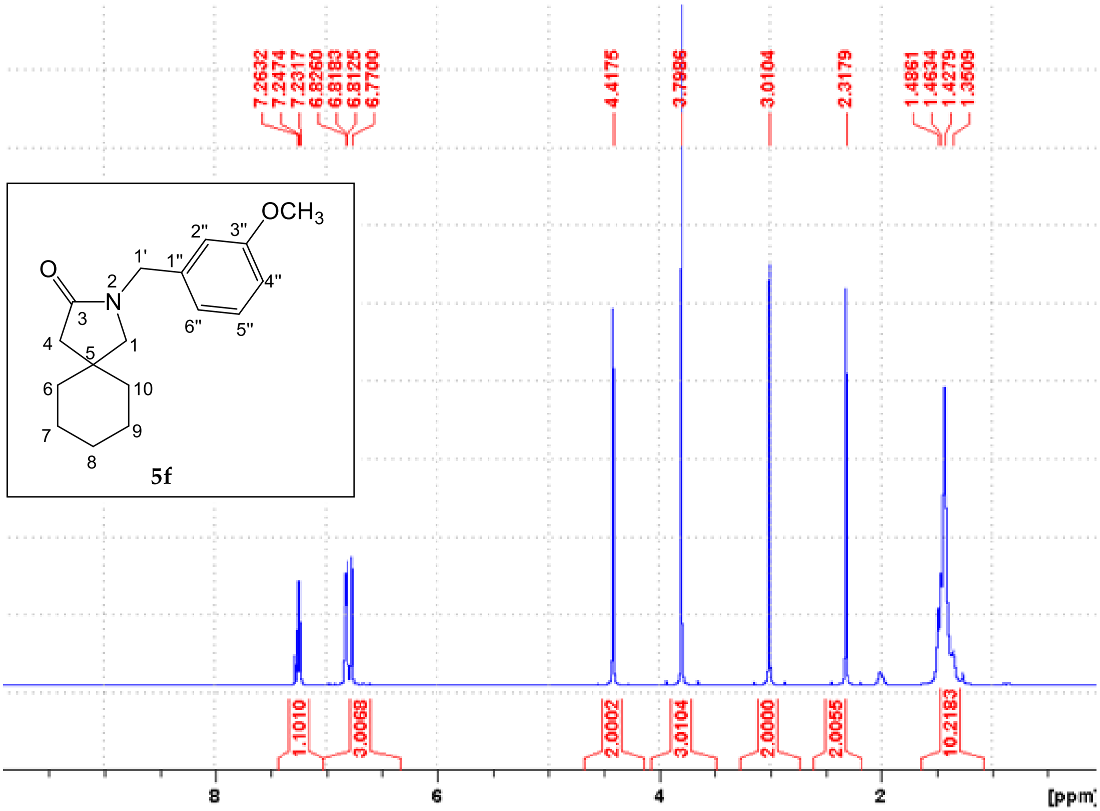

^{13}C NMR spectrum (125 MHz, $CDCl_3$) of compound **5f**:

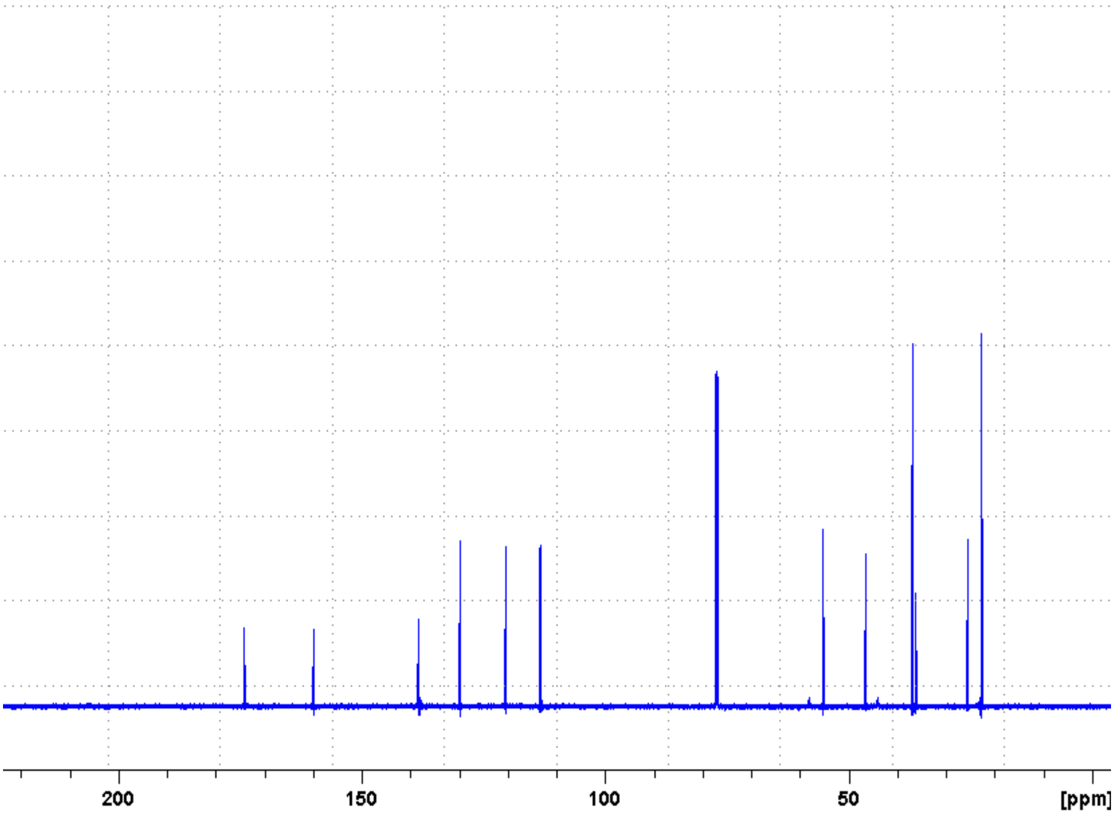

^{1}H NMR spectrum (500 MHz, $CDCl_3$) of compound **5g**:

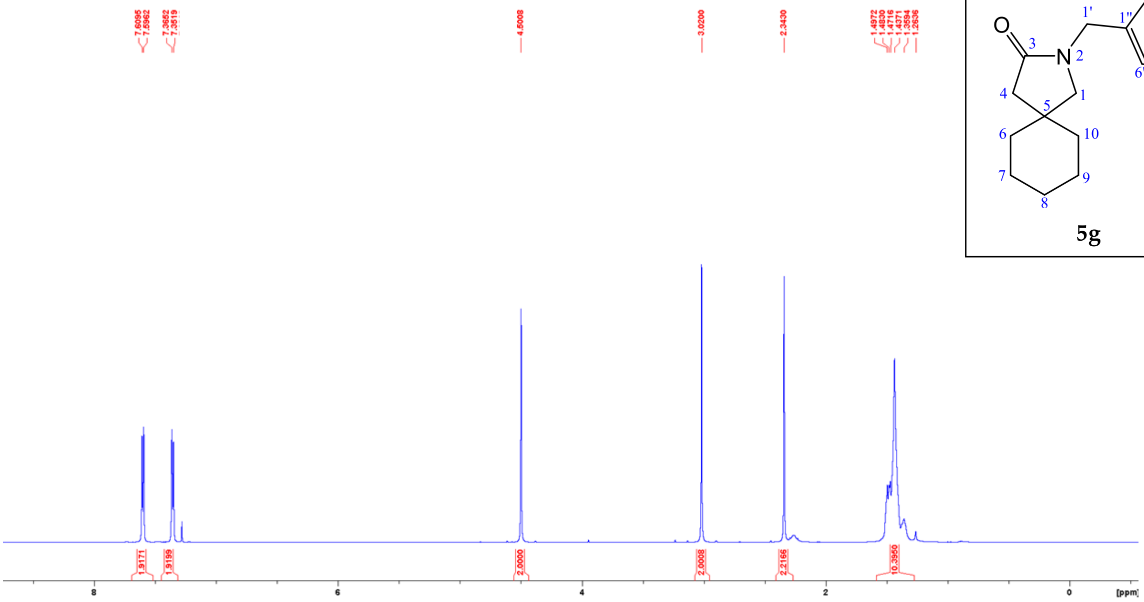

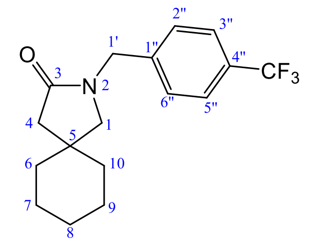

5g

^{13}C NMR spectrum (125 MHz, $CDCl_3$) of compound **5g**:

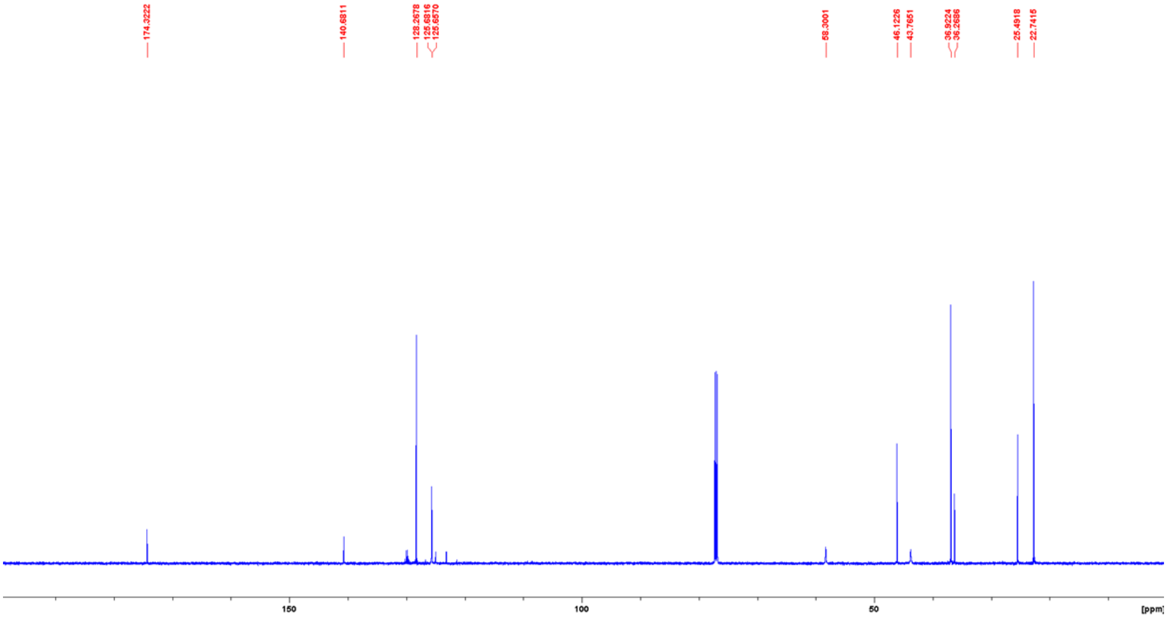

^{1}H NMR spectrum (500 MHz, $CDCl_3$) of compound **5h**:

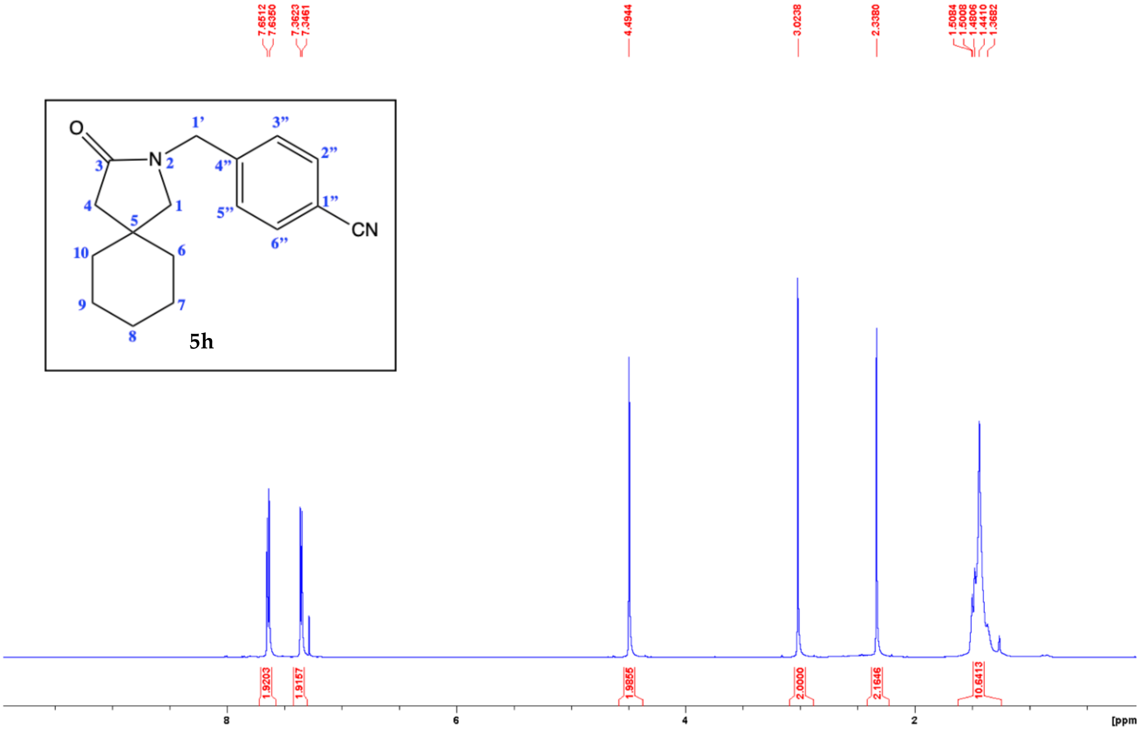

^{13}C NMR spectrum (125 MHz, $CDCl_3$) of compound **5h**:

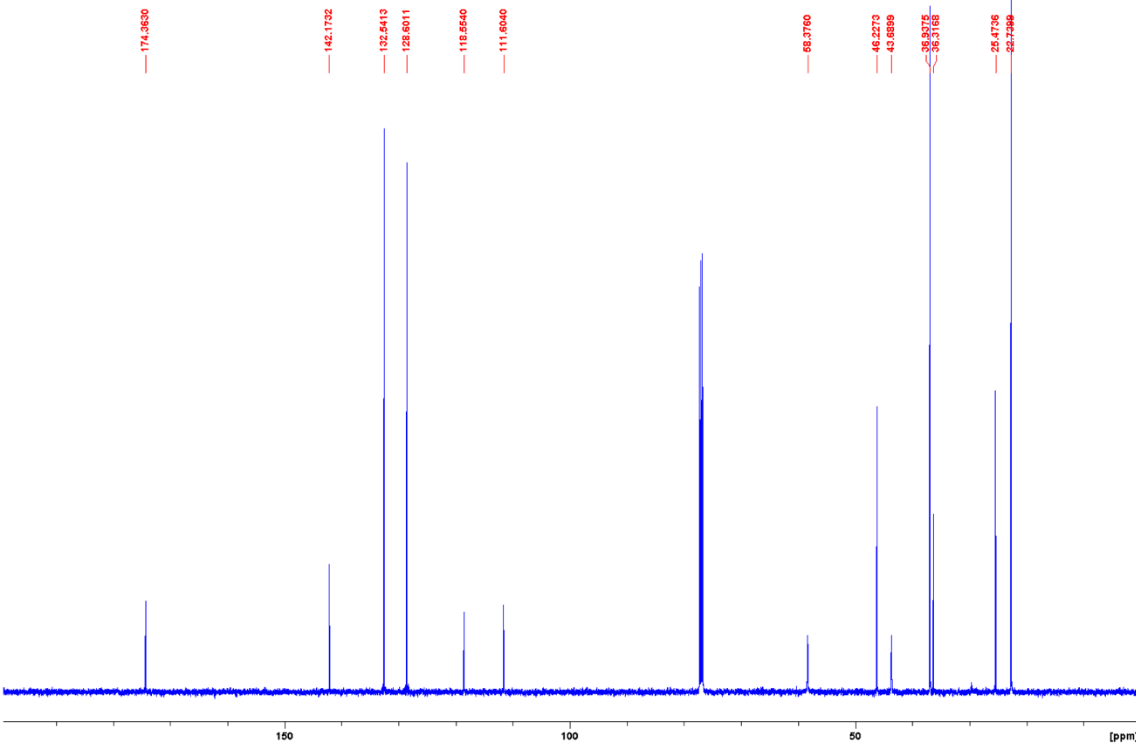

^{1}H NMR spectrum (500 MHz, $CDCl_3$) of compound **5i**:

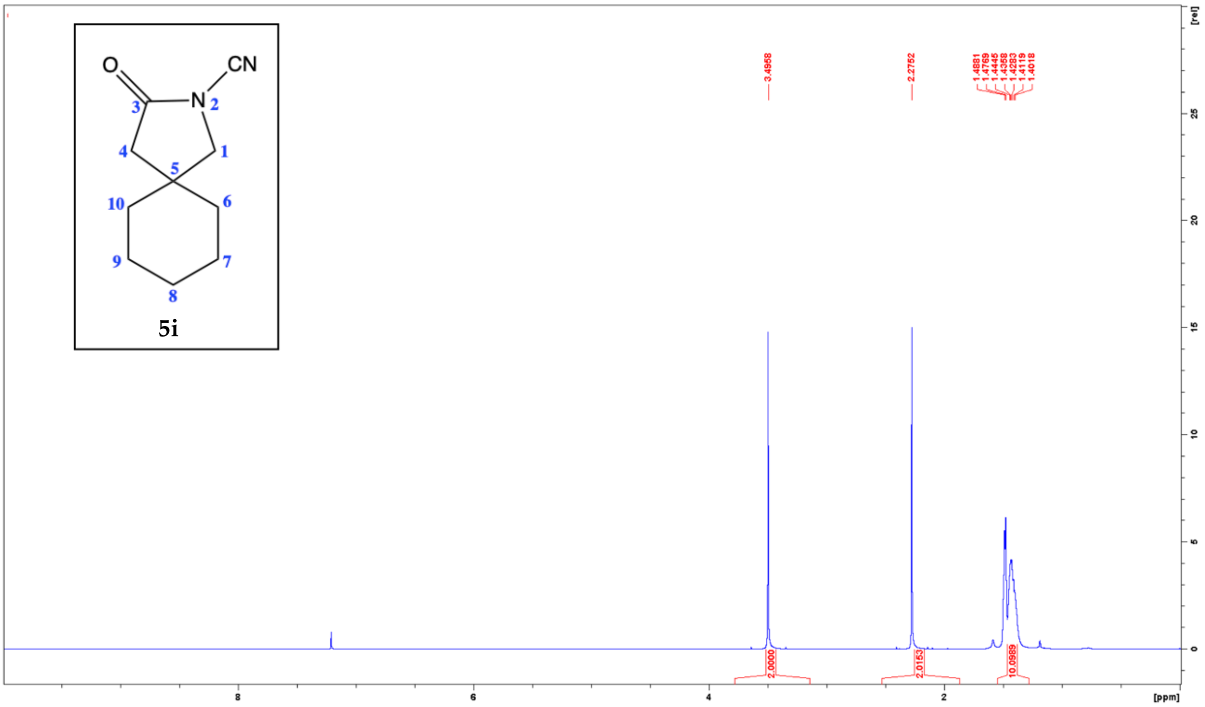

^{13}C NMR spectrum (125 MHz, $CDCl_3$) of compound **5i**:

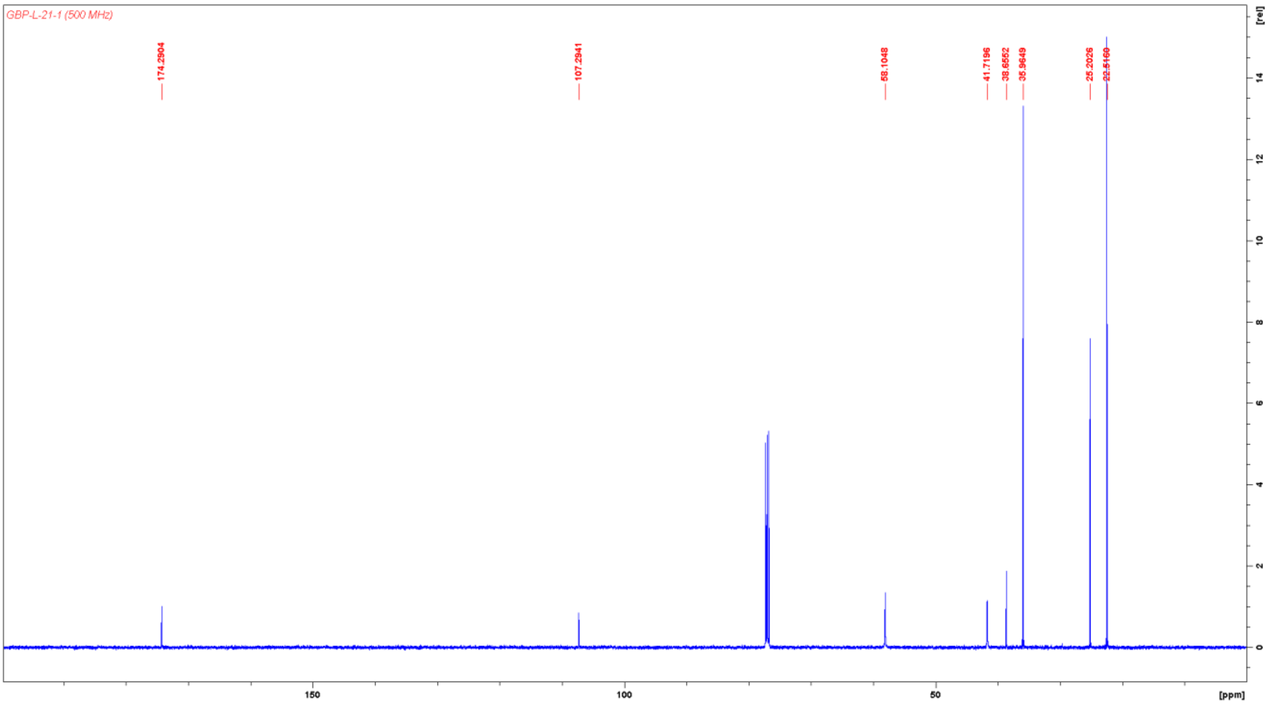

^{1}H NMR spectrum (500 MHz, $CDCl_3$) of compound **5j**:

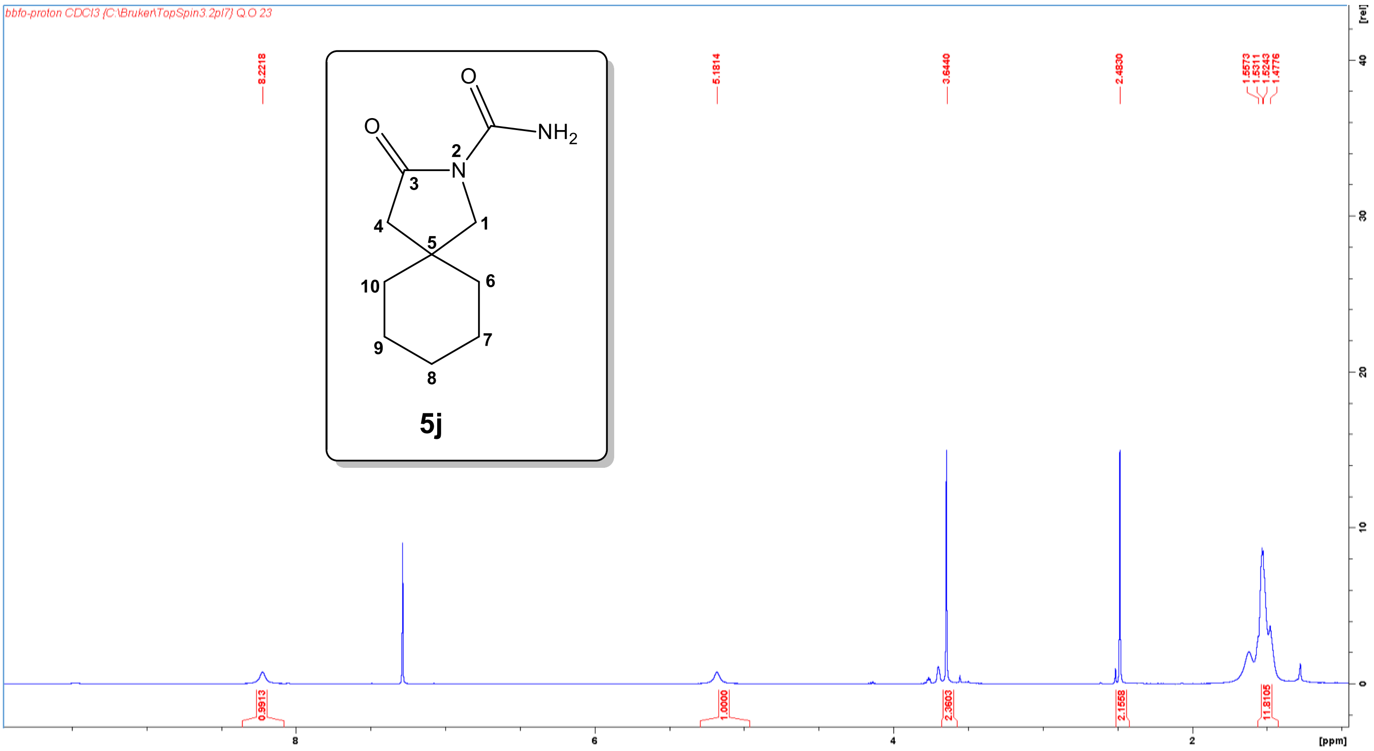

^{13}C NMR spectrum (125 MHz, $CDCl_3$) of compound **5j**:

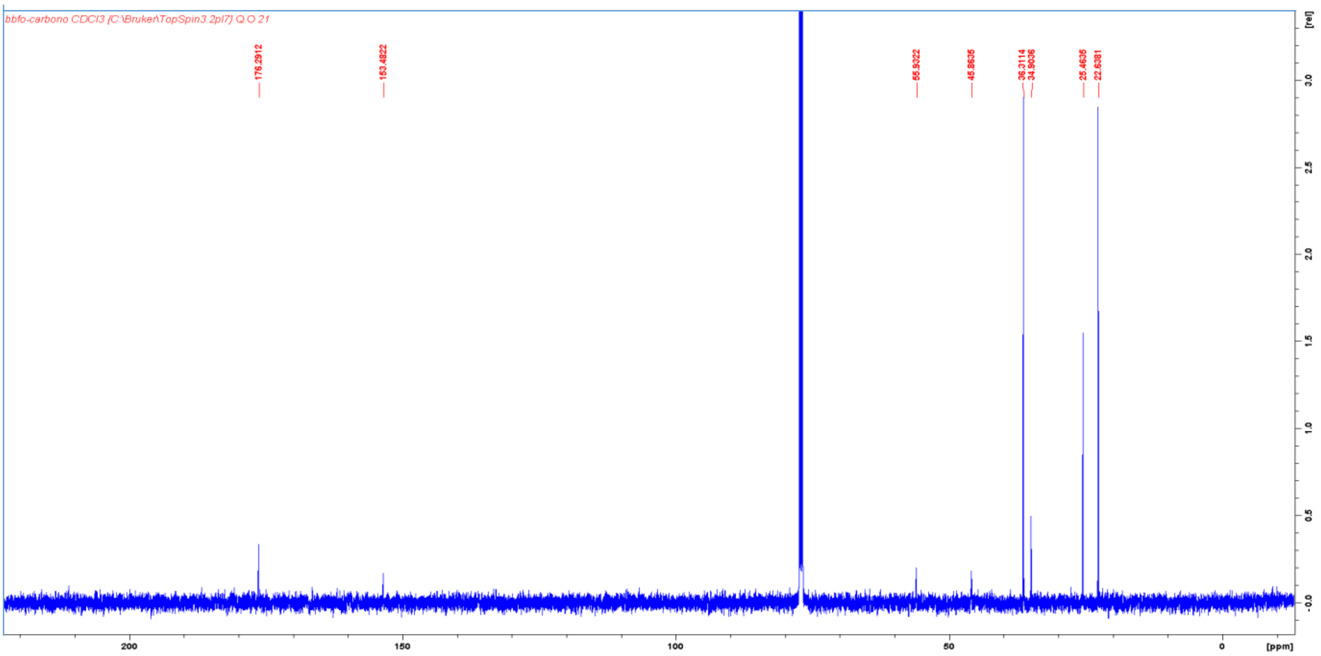

^{1}H NMR spectrum (500 MHz, $CDCl_3$) of compound **5k**:

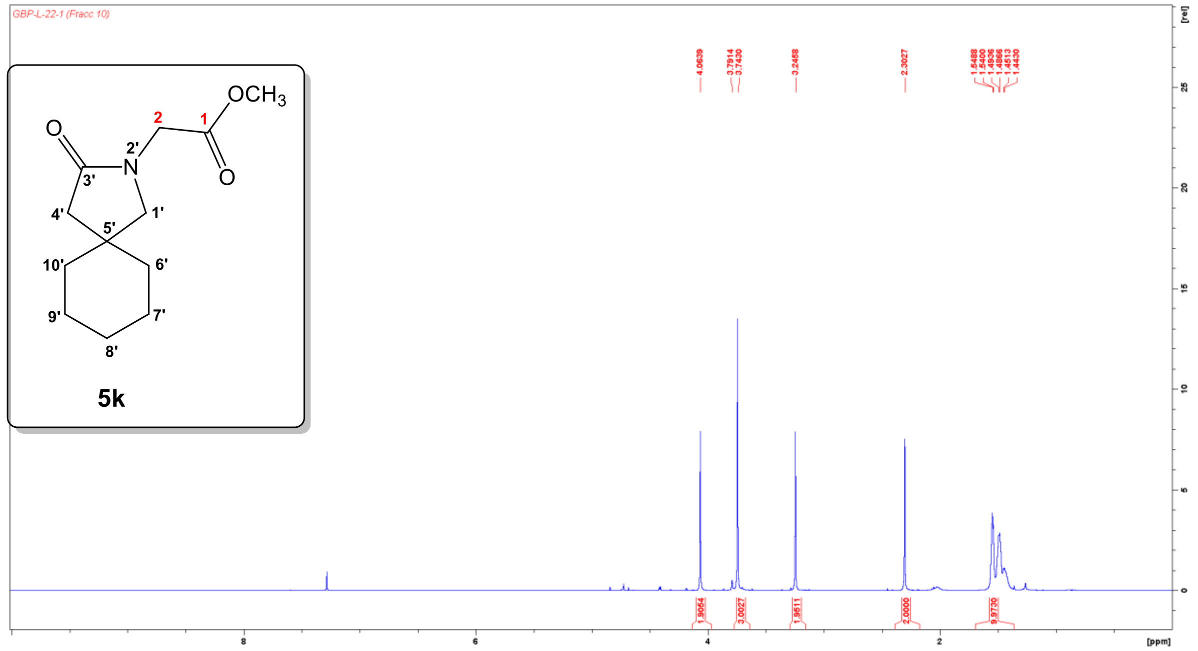

^{13}C NMR spectrum (125 MHz, $CDCl_3$) of compound **5k**:

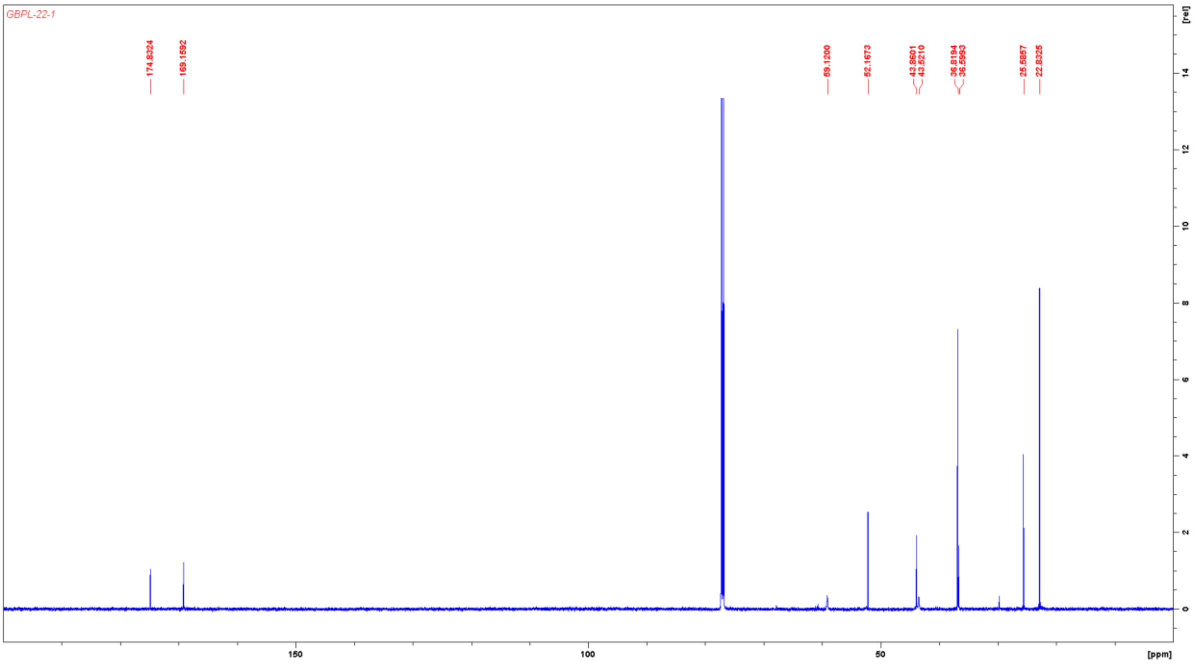

^{1}H NMR spectrum (500 MHz, $CDCl_3$) of compound **5l:**

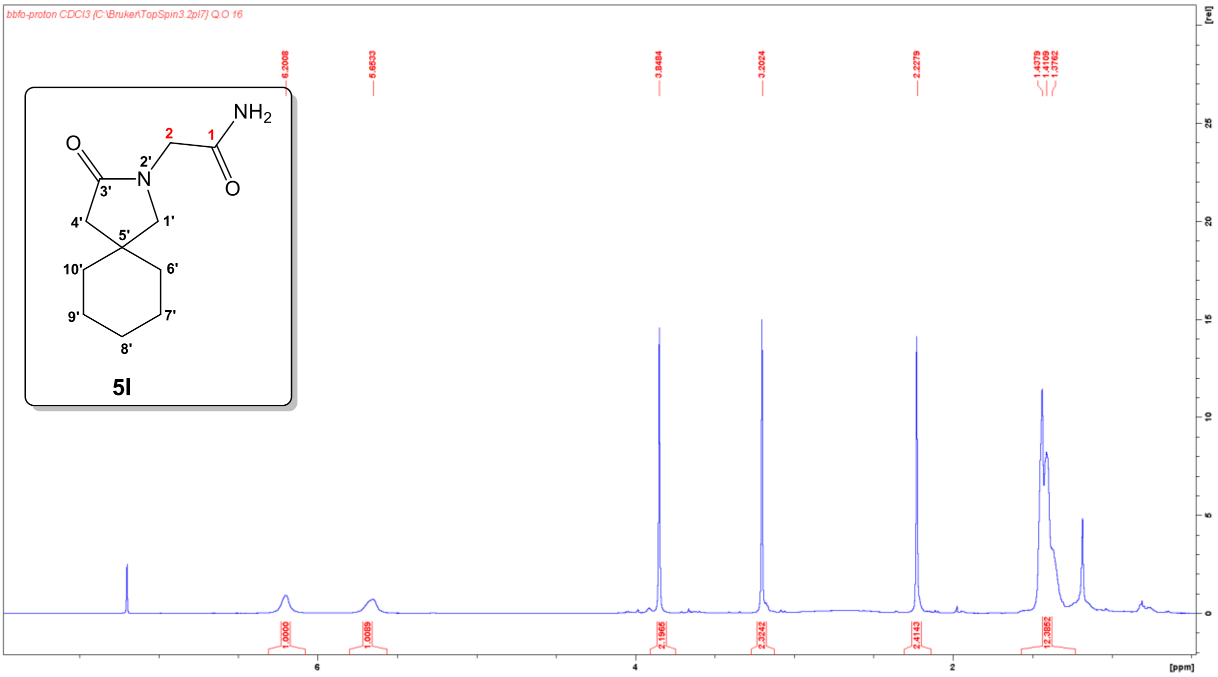

^{13}C NMR spectrum (125 MHz, $CDCl_3$) of compound **5l**:

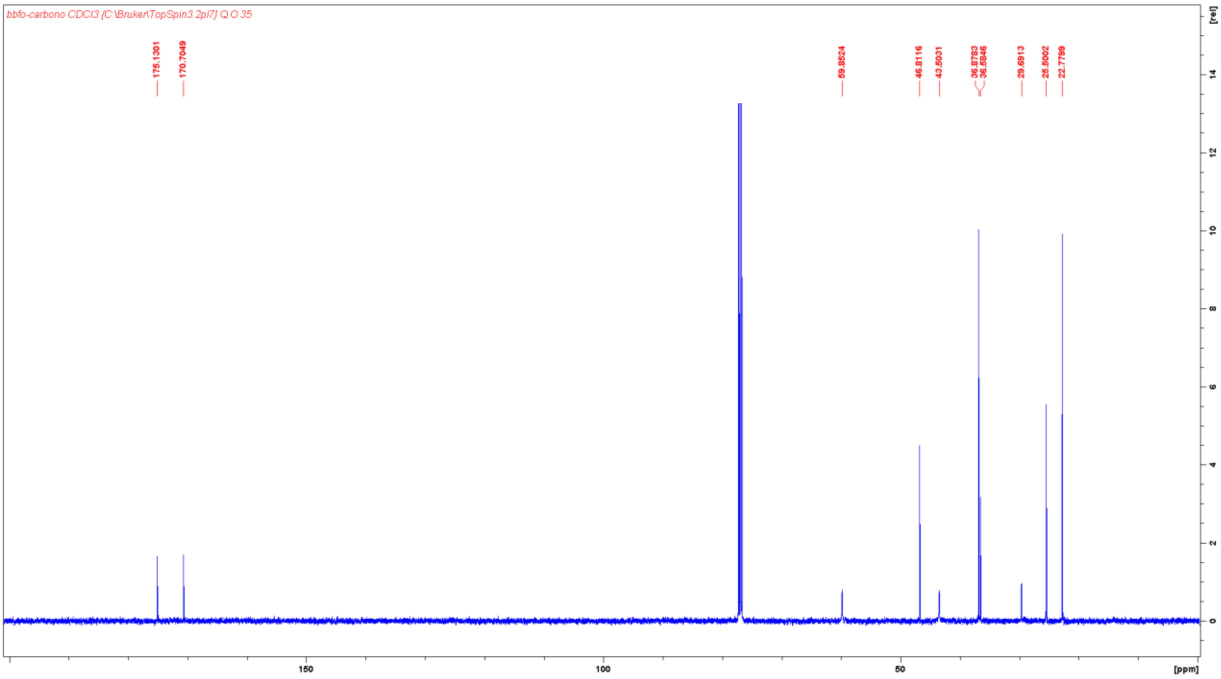

^{1}H NMR spectrum (500 MHz, $CDCl_3$) of compound **6**:

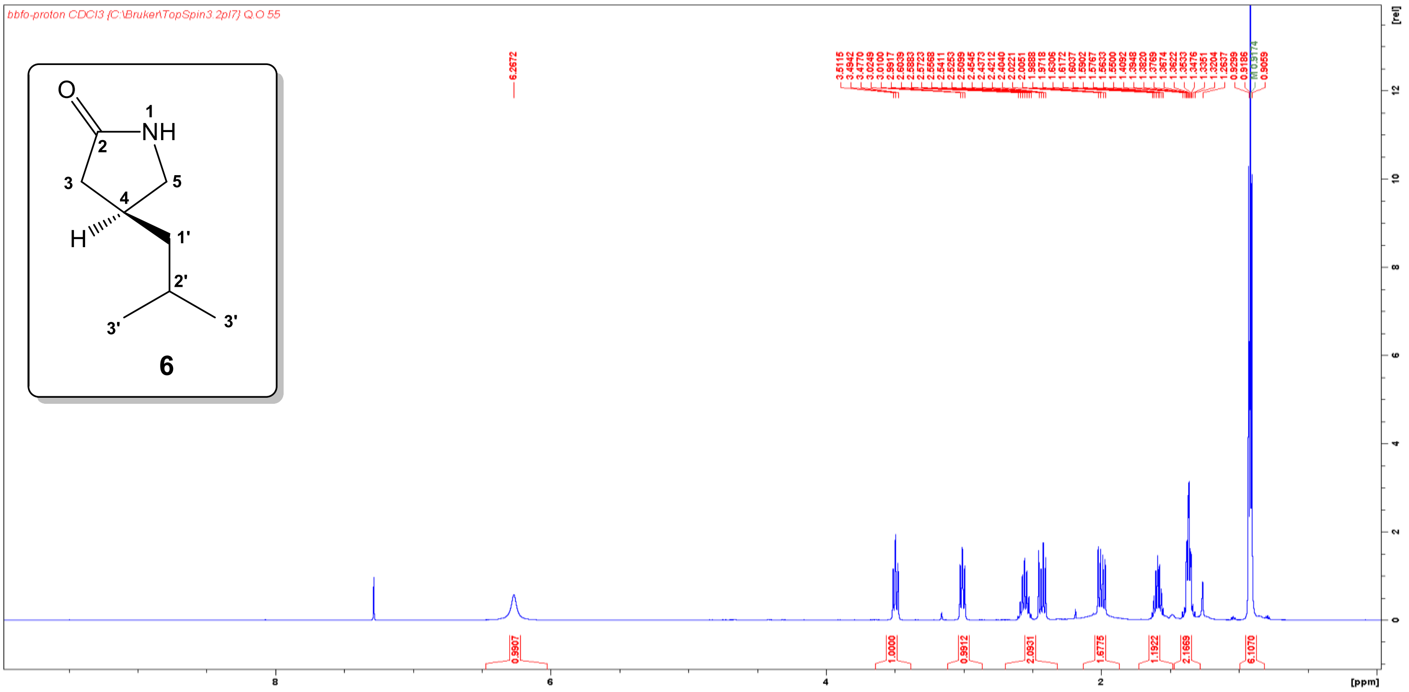

^{13}C NMR spectrum (125 MHz, $CDCl_3$) of compound **6**:

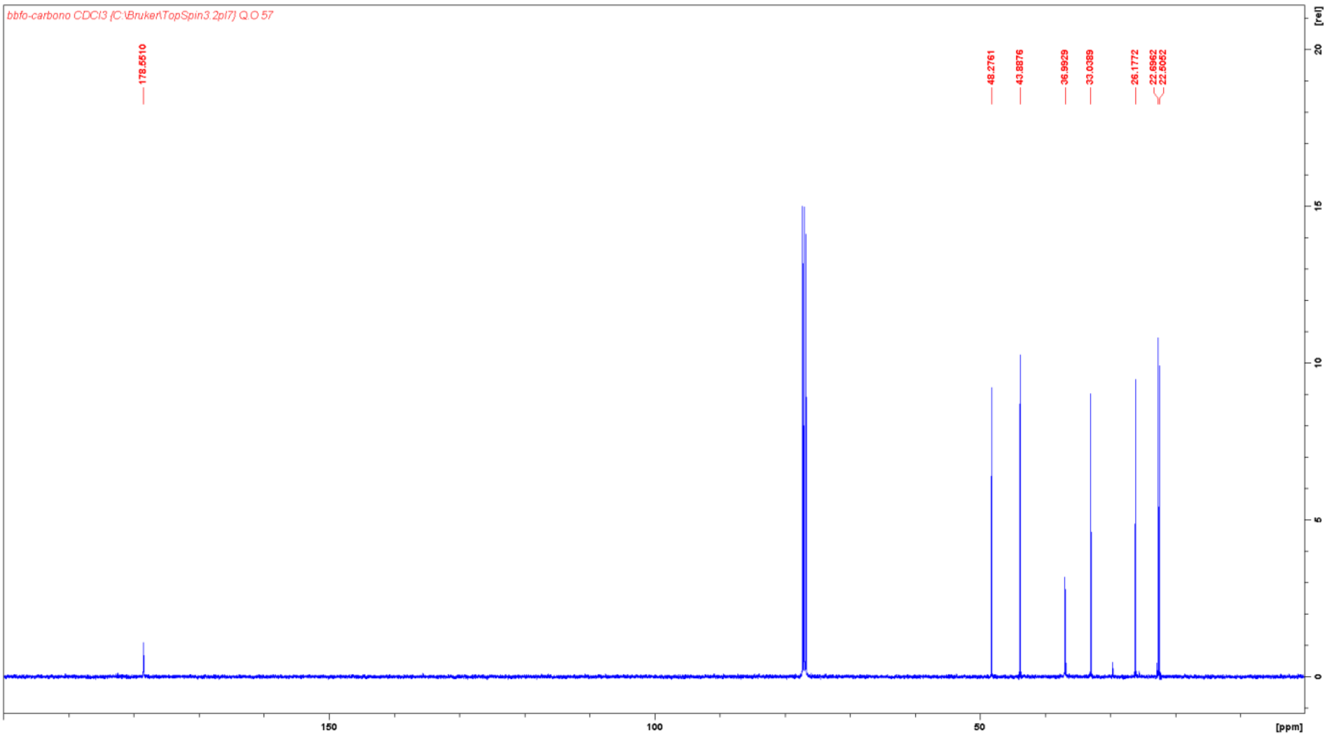

COSY spectrum (500 MHz, $CDCl_3$) of compound **6**:

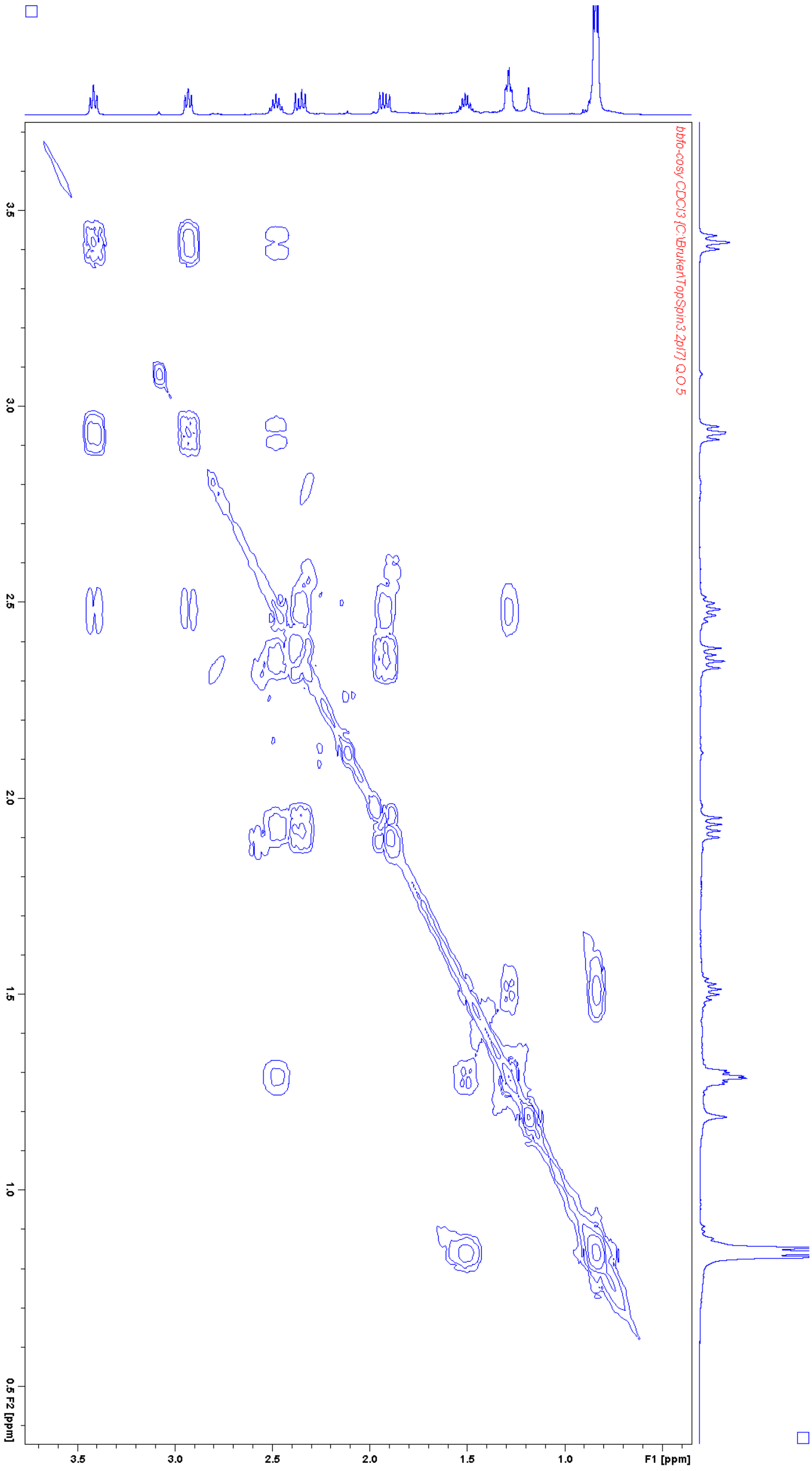

^{1}H NMR spectrum (500 MHz, $CDCl_3$) of compound **6a**:

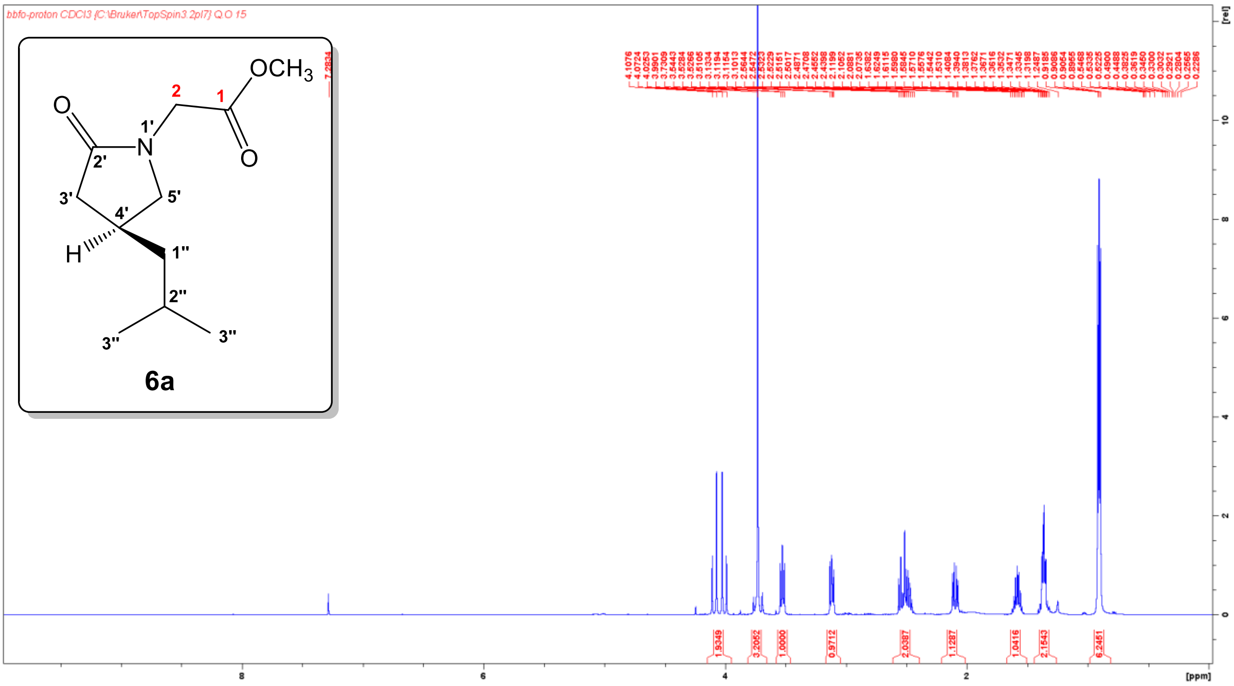

^{13}C NMR spectrum (125 MHz, $CDCl_3$) of compound **6a**:

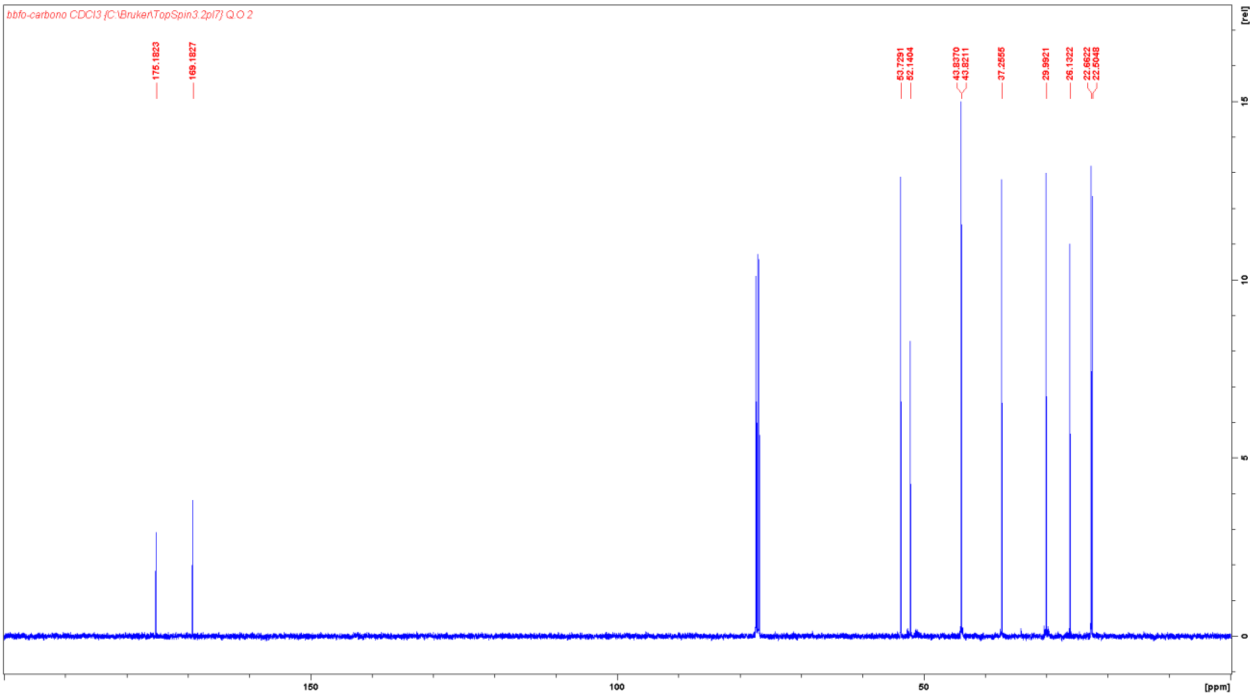

^{1}H NMR spectrum (500 MHz, $CDCl_3$) of compound **6b**:

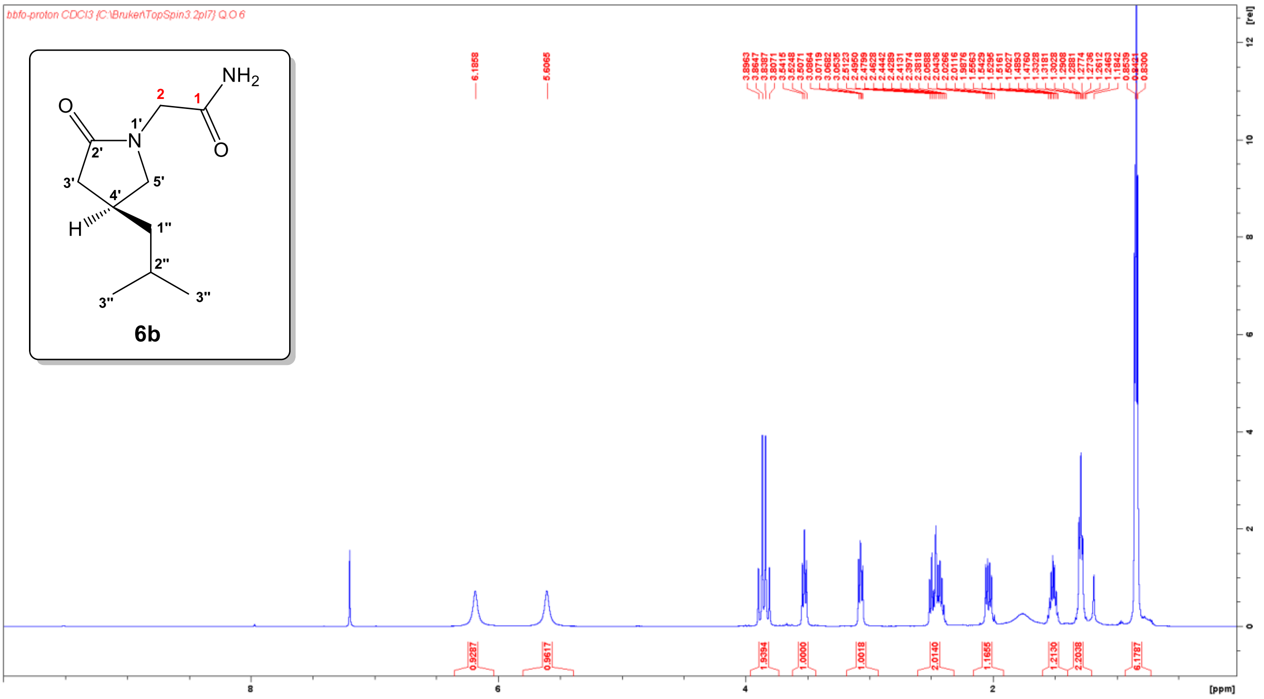

^{13}C NMR spectrum (125 MHz, $CDCl_3$) of compound **6b**:

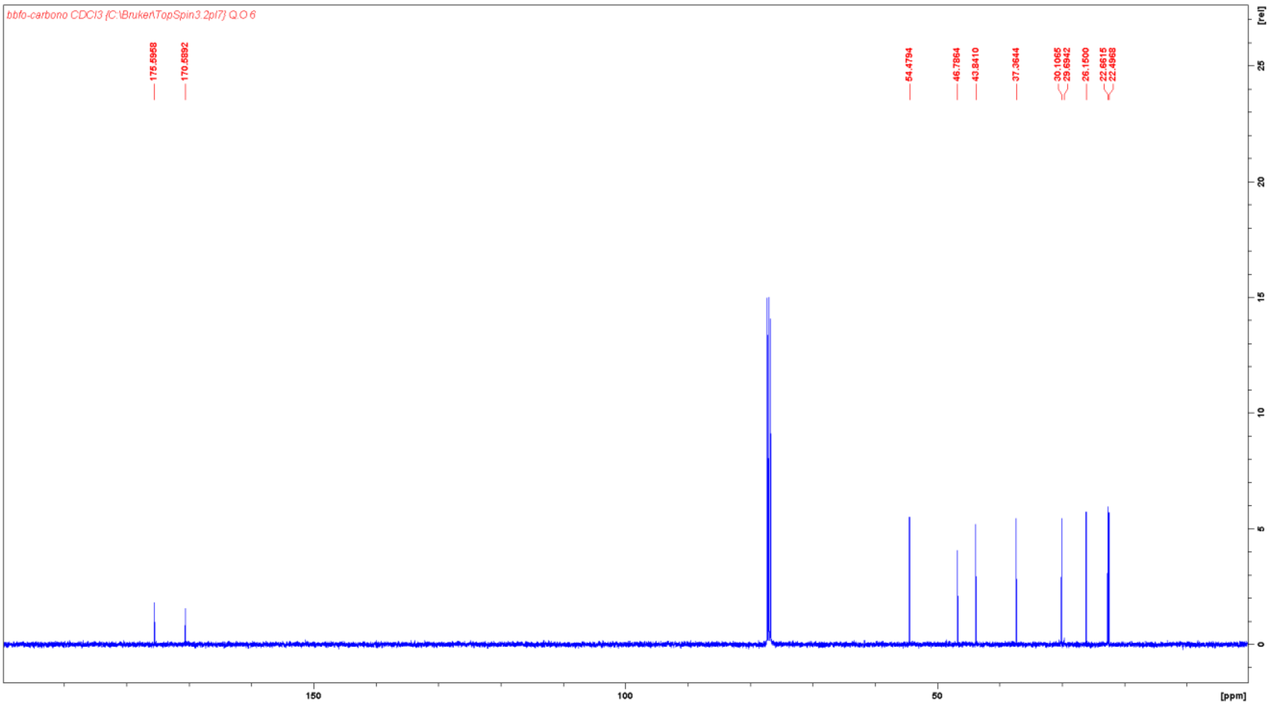

HSQCed spectrum (500 MHz, $CDCl_3$) of compound **6b**:

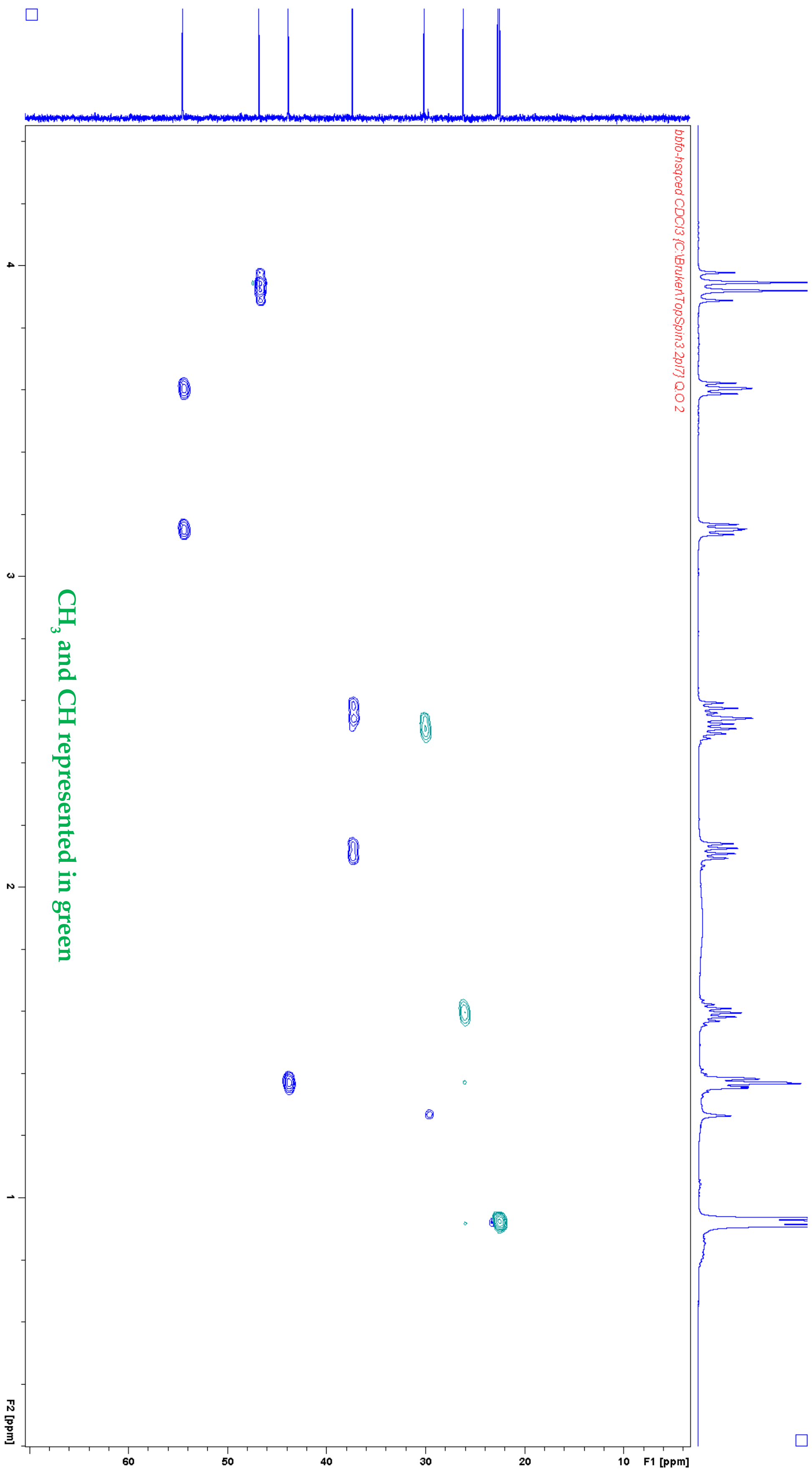

Copies of the High Resolution Mass Spectra

HRMS of compound **4**:

Page 1

Elemental Composition Report

Multiple Mass Analysis: 4368 mass(es) processed - displaying only valid results

Tolerance = 10.0 PPM / DBE: min = -1.5, max = 50.0

Selected filters: None

Monoisotopic Mass, Odd and Even Electron Ions

5881 formula(e) evaluated with 7 results within limits (all results (up to 1000) for each mass)

Elements Used:

C: 9-9 H: 5-17 N: 0-1

2101418-07Jul21_B 100 (3.961)

Magnet EI+

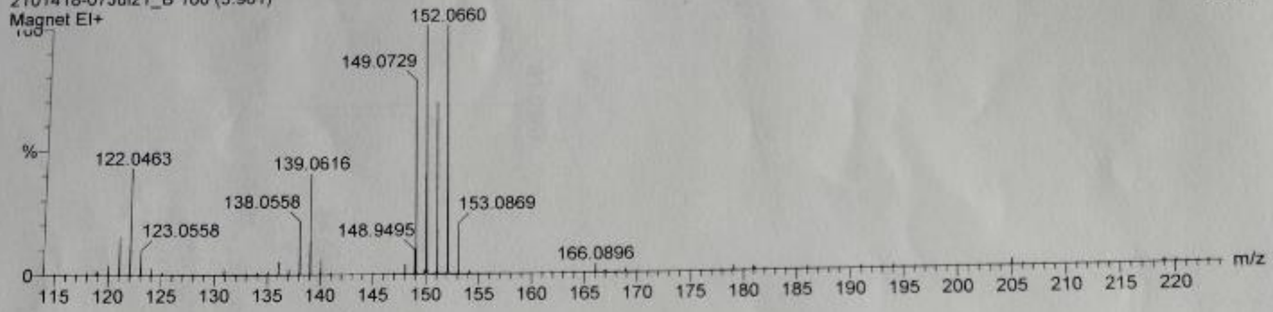

Minimum:	0.10				-1.5	
Maximum:	100.00		5.0	10.0	50.0	
Mass	RA	Calc. Mass	mDa	PPM	DBE	Formula
139.1371	0.19	139.1361	1.0	7.2	2.0	C9 H17 N
139.1358	0.18	139.1361	-0.3	-2.2	2.0	C9 H17 N
127.0432	0.14	127.0422	1.0	7.9	8.0	C9 H5 N
127.0420	0.14	127.0422	-0.2	-1.6	8.0	C9 H5 N
122.1102	0.15	122.1096	0.6	4.9	3.0	C9 H14
122.1091	0.17	122.1096	-0.5	-4.1	3.0	C9 H14
121.1029	0.12	121.1017	1.2	9.9	3.5	C9 H13

HRMS of compound **5**:

Elemental Composition Report

Page

Multiple Mass Analysis: 44 mass(es) processed - displaying only valid results
Tolerance = 10.0 PPM / DBE: min = -1.5, max = 50.0
Selected filters: None

Monoisotopic Mass, Odd and Even Electron Ions
160 formula(e) evaluated with 6 results within limits (all results (up to 1000) for each mass)
Elements Used:
C: 4-9 H: 2-15 N: 0-1 O: 0-1
Tere R14-5
21Feb19-AAFAMMA 44 (2.442) Cm (42:44-8:10)

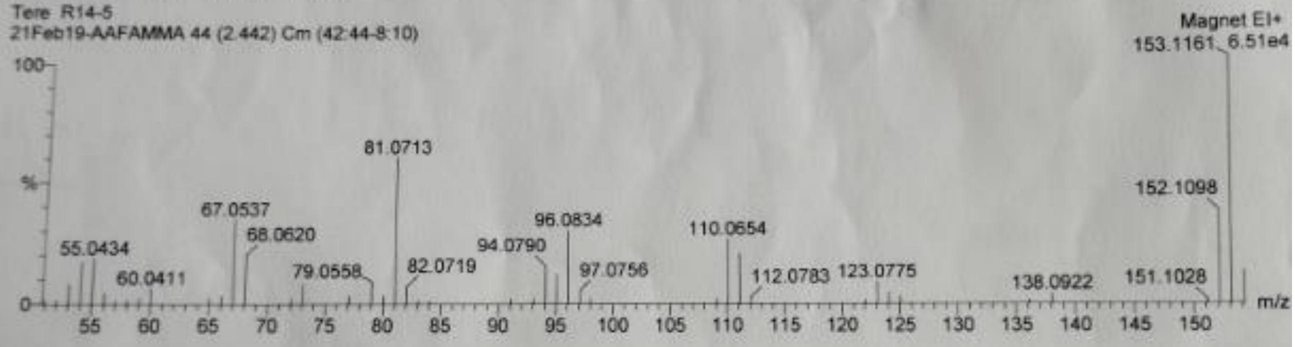

Mass	RA	Calc. Mass	mDa	PPM	DBE	i-FIT	Formula
Minimum:	0.10				-1.5		
Maximum:	100.00		5.0	10.0	50.0		
153.1161	100.00	153.1154	0.7	4.6	3.0	2800430.5	C9 H15 N O
138.0922	3.69	138.0919	0.3	2.2	3.5	5547163.0	C8 H12 N O
124.0765	4.41	124.0762	0.3	2.4	3.5	2773810.3	C7 H10 N O
94.0790	15.36	94.0783	0.7	7.4	3.0	13096.4	C7 H10
93.0703	1.79	93.0704	-0.1	-1.1	3.5	9147.5	C7 H9
68.0620	19.93	68.0626	-0.6	-8.8	2.0	5552344.5	C5 H8

HRMS of compound **5a**:

Elemental Composition Report

Multiple Mass Analysis: 4915 mass(es) processed - displaying only valid results
Tolerance = 10.0 PPM / DBE: min = -1.5, max = 50.0
Selected filters: None

Monoisotopic Mass, Odd and Even Electron Ions
14211 formula(e) evaluated with 2 results within limits (all results (up to 1000) for each mass)
Elements Used:
C: 10-10 H: 17-17 N: 0-1 O: 0-1

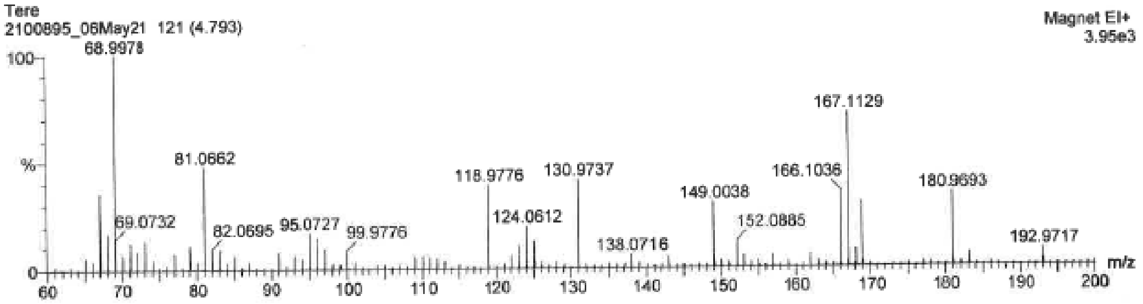

Minimum:	0.30				-1.5	
Maximum:	100.00		5.0	10.0	50.0	
Mass	RA	Calc. Mass	mDa	PPM	DBE	Formula
167.1298	0.46	167.1310	-1.2	-7.2	3.0	C10 H17 N O
151.1353	0.33	151.1361	-0.8	-5.3	3.0	C10 H17 N

HRMS of compound **5b**:

Elemental Composition Report

Multiple Mass Analysis: 1414 mass(es) processed - displaying only valid results
Tolerance = 10.0 PPM / DBE: min = -1.5, max = 50.0
Selected filters: None

Monoisotopic Mass, Odd and Even Electron Ions
5293 formula(e) evaluated with 65 results within limits (all results (up to 1000) for each mass)
Elements Used:
C: 5-12 H: 6-19 N: 0-1 O: 0-1

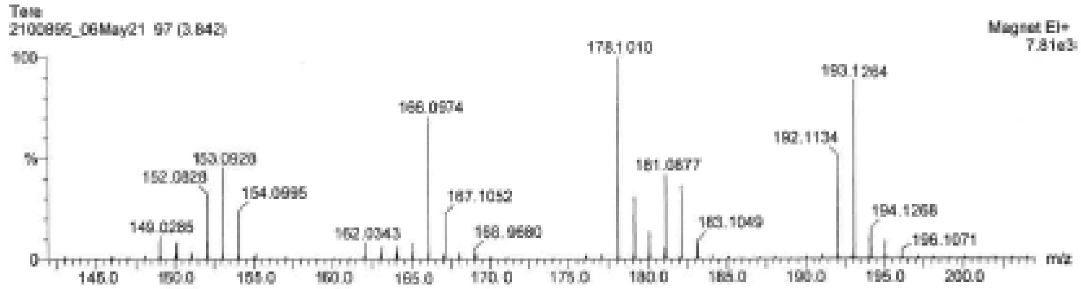

Minimum:	0.50				-1.5	
Maximum:	100.00		5.0	10.0	50.0	

Mass	RA	Calc. Mass	mDa	PPM	DBE	Formula
193.1459	0.67	193.1467	-0.8	-4.1	4.0	C12 H19 N O
180.0451	0.70	180.0449	0.2	1.1	10.5	C12 H6 N O
180.0435	0.58	180.0449	-1.4	-7.8	10.5	C12 H6 N O
178.1223	0.73	178.1232	-0.9	-5.1	4.5	C11 H16 N O
176.1211	0.67	176.1201	1.0	5.7	5.0	C12 H16 O
176.1195	0.91	176.1201	-0.6	-3.4	5.0	C12 H16 O
169.0894	2.02	169.0891	0.3	1.8	8.0	C12 H11 N
169.0878	2.59	169.0891	-1.3	-7.7	8.0	C12 H11 N
168.0820	3.12	168.0813	0.7	4.2	8.5	C12 H10 N
168.0804	3.62	168.0813	-0.9	-5.4	8.5	C12 H10 N
168.0464	0.64	168.0449	1.5	8.9	9.5	C11 H6 N O
168.0448	1.01	168.0449	-0.1	-0.6	9.5	C11 H6 N O
168.0433	1.09	168.0449	-1.6	-9.5	9.5	C11 H6 N O
167.0744	2.59	167.0735	0.9	5.4	9.0	C12 H9 N
167.0729	3.35	167.0735	-0.6	-3.6	9.0	C12 H9 N
167.0376	1.06	167.0371	0.5	3.0	10.0	C11 H5 N O
167.0360	1.18	167.0371	-1.1	-6.6	10.0	C11 H5 N O
166.0668	1.89	166.0657	1.1	6.6	9.5	C12 H8 N
166.0653	2.38	166.0657	-0.4	-2.4	9.5	C12 H8 N
165.1292	0.92	165.1279	1.3	7.9	3.5	C11 H17 O
165.1277	0.52	165.1279	-0.2	-1.2	3.5	C11 H17 O
165.0593	1.00	165.0578	1.5	9.1	10.0	C12 H7 N
165.0578	1.01	165.0578	0.0	0.0	10.0	C12 H7 N
165.0562	1.11	165.0578	-1.6	-9.7	10.0	C12 H7 N
164.1213	0.83	164.1201	1.2	7.3	4.0	C11 H16 O
164.1198	0.82	164.1201	-0.3	-1.8	4.0	C11 H16 O
164.0502	2.51	164.0500	0.2	1.2	10.5	C12 H6 N
164.0488	3.08	164.0500	-1.2	-7.3	10.5	C12 H6 N
163.0429	1.14	163.0422	0.7	4.3	11.0	C12 H5 N
163.0414	1.75	163.0422	-0.8	-4.9	11.0	C12 H5 N
160.0874	0.56	160.0888	-1.4	-8.7	6.0	C11 H12 O
157.0641	0.51	157.0653	-1.2	-7.6	7.5	C11 H9 O
154.0796	0.65	154.0783	1.3	8.4	8.0	C12 H10
154.0669	2.41	154.0657	1.2	7.8	8.5	C11 H8 N
154.0654	2.73	154.0657	-0.3	-1.9	8.5	C11 H8 N
153.0716	4.52	153.0704	1.2	7.8	8.5	C12 H9
153.0702	3.98	153.0704	-0.2	-1.3	8.5	C12 H9
153.0589	2.34	153.0578	1.1	7.2	9.0	C11 H7 N
153.0575	2.39	153.0578	-0.3	-2.0	9.0	C11 H7 N
152.1080	0.68	152.1075	0.5	3.3	3.5	C9 H14 N O
152.1066	0.90	152.1075	-0.9	-5.9	3.5	C9 H14 N O
152.0633	0.60	152.0626	0.7	4.6	9.0	C12 H8

HRMS of compound **5c**:

Elemental Composition Report

Multiple Mass Analysis: 957 mass(es) processed - displaying only valid results

Tolerance = 10.0 PPM / DBE: min = -1.5, max = 50.0

Selected filters: None

Monoisotopic Mass, Odd and Even Electron Ions

3358 formula(e) evaluated with 38 results within limits (all results (up to 1000) for each mass)

Elements Used:

C: 14-15 H: 1-25 N: 0-1 O: 0-1

Tere GBPL - 22 -

2202106_21Nov22 82 (3.248)

Magnet EI+ 1.38e4

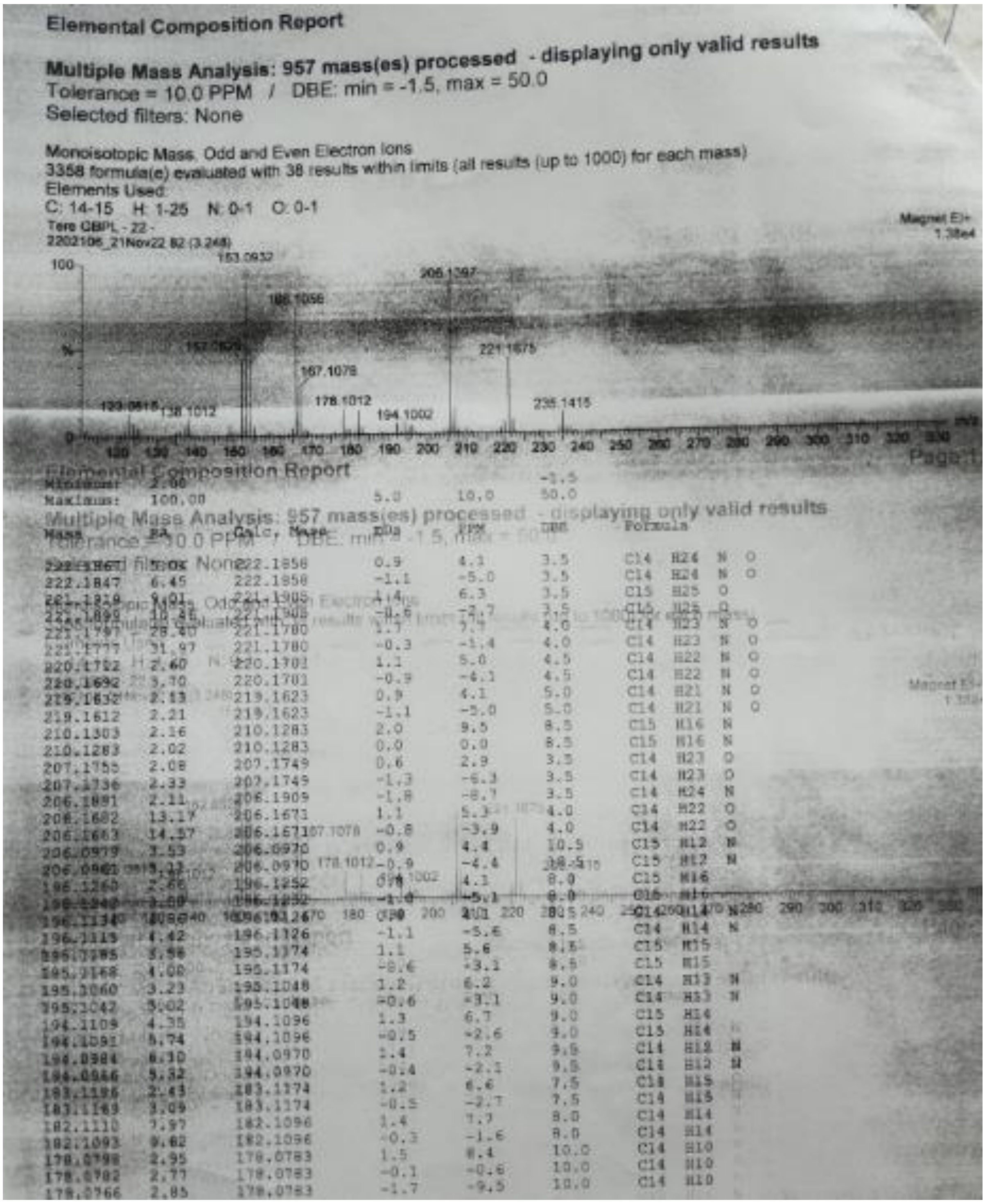

Minimum:	2.00				-1.5	
Maximum:	100.00		5.0	10.0	50.0	

Mass	RA	Calc. Mass	mDa	PPM	DBE	Formula
[illegible]	[illegible]	222.1858	0.9	4.1	3.5	C14 H24 N O
222.1847	6.45	222.1858	-1.1	-5.0	3.5	C14 H24 N O
221.1919	9.01	221.1905	1.4	6.3	3.5	C15 H25 O
221.1899	10.46	221.1905	-0.6	-2.7	3.5	C15 H25 O
221.1797	28.40	221.1780	1.7	7.7	4.0	C14 H23 N O
221.1777	31.97	221.1780	-0.3	-1.4	4.0	C14 H23 N O
220.1712	2.60	220.1701	1.1	5.0	4.5	C14 H22 N O
220.1692	3.70	220.1701	-0.9	-4.1	4.5	C14 H22 N O
219.1632	2.13	219.1623	0.9	4.1	5.0	C14 H21 N O
219.1612	2.21	219.1623	-1.1	-5.0	5.0	C14 H21 N O
210.1303	2.16	210.1283	2.0	9.5	8.5	C15 H16 N
210.1283	2.02	210.1283	0.0	0.0	8.5	C15 H16 N
207.1755	2.08	207.1749	0.6	2.9	3.5	C14 H23 O
207.1736	2.33	207.1749	-1.3	-6.3	3.5	C14 H23 O
206.1891	2.11	206.1909	-1.8	-8.7	3.5	C14 H24 N
206.1682	13.17	206.1671	1.1	5.3	4.0	C14 H22 O
206.1663	14.57	206.1671	-0.8	-3.9	4.0	C14 H22 O
206.0979	3.53	206.0970	0.9	4.4	10.5	C15 H12 N
206.0961	[illegible]	206.0970	-0.9	-4.4	10.5	C15 H12 N
196.1260	2.66	196.1252	0.8	4.1	8.0	C15 H16
196.1242	3.00	196.1252	-1.0	-5.1	8.0	C15 H16
196.1134	[illegible]	[illegible]	[illegible]	[illegible]	[illegible]	C14 H14 N
196.1115	4.42	196.1126	-1.1	-5.6	8.5	C14 H14 N
195.1185	3.98	195.1174	1.1	5.6	8.5	C15 H15
195.1168	4.00	195.1174	-0.6	-3.1	8.5	C15 H15
195.1060	3.23	195.1048	1.2	6.2	9.0	C14 H13 N
195.1042	5.02	195.1048	-0.6	-3.1	9.0	C14 H13 N
194.1109	4.35	194.1096	1.3	6.7	9.0	C15 H14
194.1091	5.74	194.1096	-0.5	-2.6	9.0	C15 H14
194.0984	6.10	194.0970	1.4	7.2	9.5	C14 H12 N
194.0966	5.32	194.0970	-0.4	-2.1	9.5	C14 H12 N
183.1186	2.43	183.1174	1.2	6.6	7.5	C14 H15
183.1169	3.09	183.1174	-0.5	-2.7	7.5	C14 H15
182.1110	7.97	182.1096	1.4	7.7	8.0	C14 H14
182.1093	9.62	182.1096	-0.3	-1.6	8.0	C14 H14
178.0798	2.95	178.0783	1.5	8.4	10.0	C14 H10
178.0782	2.77	178.0783	-0.1	-0.6	10.0	C14 H10
178.0766	2.85	178.0783	-1.7	-9.5	10.0	C14 H10

HRMS of compound **5d**:

Elemental Composition Report

Multiple Mass Analysis: 373 mass(es) processed - displaying only valid results
Tolerance = 10.0 PPM / DBE: min = -1.5, max = 50.0
Selected filters: None

Monoisotopic Mass, Odd and Even Electron Ions
4828 formula(e) evaluated with 110 results within limits (all results (up to 1000) for each mass)
Elements Used:
C: 5-15 H: 5-25 N: 0-1 O: 0-3
Tere GBPL - 22 -
2202108_21Nov22_B 55 (2.178)

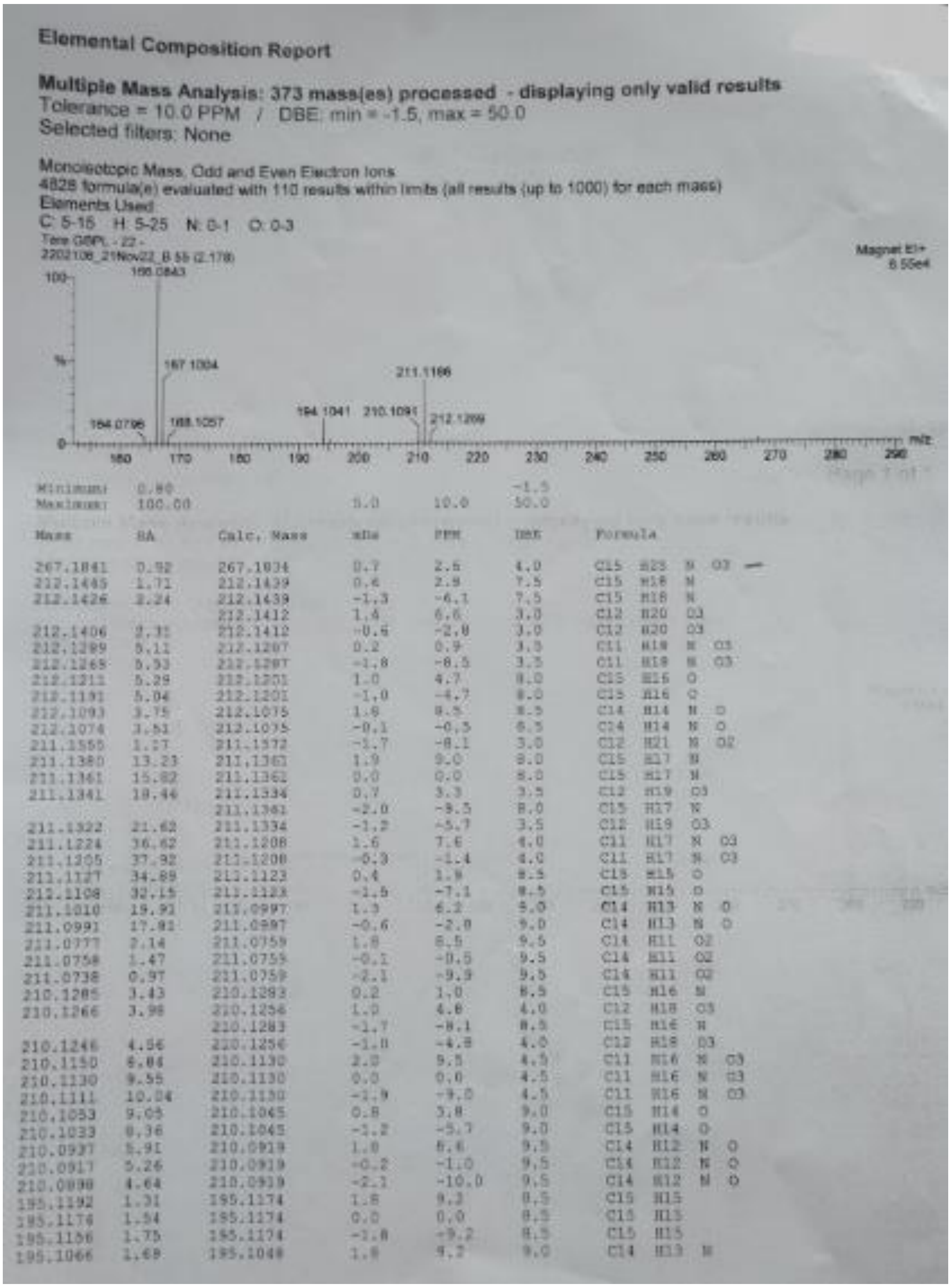

Page 1 of 1

Minimum:	0.80				-1.5	
Maximum:	100.00		5.0	10.0	50.0	

Mass	RA	Calc. Mass	mDa	PPM	DBE	Formula
267.1841	0.92	267.1834	0.7	2.6	4.0	C15 H25 N O3 —
212.1445	1.71	212.1439	0.6	2.8	7.5	C15 H18 N
212.1426	2.24	212.1439	-1.3	-6.1	7.5	C15 H18 N
		212.1412	1.4	6.6	3.0	C12 H20 O3
212.1406	2.31	212.1412	-0.6	-2.8	3.0	C12 H20 O3
212.1289	5.11	212.1287	0.2	0.9	3.5	C11 H18 N O3
212.1269	5.93	212.1287	-1.8	-8.5	3.5	C11 H18 N O3
212.1211	5.29	212.1201	1.0	4.7	8.0	C15 H16 O
212.1191	5.04	212.1201	-1.0	-4.7	8.0	C15 H16 O
212.1093	3.75	212.1075	1.8	8.5	8.5	C14 H14 N O
212.1074	3.51	212.1075	-0.1	-0.5	8.5	C14 H14 N O
211.1555	1.27	211.1572	-1.7	-8.1	3.0	C12 H21 N O2
211.1380	13.23	211.1361	1.9	9.0	8.0	C15 H17 N
211.1361	15.82	211.1361	0.0	0.0	8.0	C15 H17 N
211.1341	18.46	211.1334	0.7	3.3	3.5	C12 H19 O3
		211.1361	-2.0	-9.5	8.0	C15 H17 N
211.1322	21.62	211.1334	-1.2	-5.7	3.5	C12 H19 O3
211.1224	36.62	211.1208	1.6	7.6	4.0	C11 H17 N O3
211.1205	37.92	211.1208	-0.3	-1.4	4.0	C11 H17 N O3
211.1127	34.89	211.1123	0.4	1.9	8.5	C15 H15 O
211.1108	32.15	211.1123	-1.5	-7.1	8.5	C15 H15 O
211.1010	19.91	211.0997	1.3	6.2	9.0	C14 H13 N O
211.0991	17.81	211.0997	-0.6	-2.8	9.0	C14 H13 N O
211.0777	2.14	211.0759	1.8	8.5	9.5	C14 H11 O2
211.0758	1.47	211.0759	-0.1	-0.5	9.5	C14 H11 O2
211.0738	0.97	211.0759	-2.1	-9.9	9.5	C14 H11 O2
210.1285	3.43	210.1283	0.2	1.0	8.5	C15 H16 N
210.1266	3.98	210.1256	1.0	4.8	4.0	C12 H18 O3
		210.1283	-1.7	-8.1	8.5	C15 H16 N
210.1246	4.56	210.1256	-1.0	-4.8	4.0	C12 H18 O3
210.1150	6.04	210.1130	2.0	9.5	4.5	C11 H16 N O3
210.1130	9.55	210.1130	0.0	0.0	4.5	C11 H16 N O3
210.1111	10.04	210.1130	-1.9	-9.0	4.5	C11 H16 N O3
210.1053	9.05	210.1045	0.8	3.8	9.0	C15 H14 O
210.1033	8.36	210.1045	-1.2	-5.7	9.0	C15 H14 O
210.0937	5.91	210.0919	1.8	8.6	9.5	C14 H12 N O
210.0917	5.26	210.0919	-0.2	-1.0	9.5	C14 H12 N O
210.0898	4.64	210.0919	-2.1	-10.0	9.5	C14 H12 N O
195.1192	1.31	195.1174	1.8	9.2	8.5	C15 H15
195.1174	1.54	195.1174	0.0	0.0	8.5	C15 H15
195.1156	1.75	195.1174	-1.8	-9.2	8.5	C15 H15
195.1066	1.69	195.1048	1.8	9.2	9.0	C14 H13 N

HRMS of compound **5e**:

Elemental Composition Report

Multiple Mass Analysis: 85 mass(es) processed - displaying only valid results
Tolerance = 10.0 PPM / DBE: min = -1.5, max = 50.0
Selected filters: None

Monoisotopic Mass, Odd and Even Electron Ions
420 formula(e) evaluated with 4 results within limits (all results (up to 1000) for each mass)
Elements Used:
C: 5-16 H: 5-21 N: 0-1 O: 0-1
Tere
2100895_06May21 AFAMM 55 (2.178)

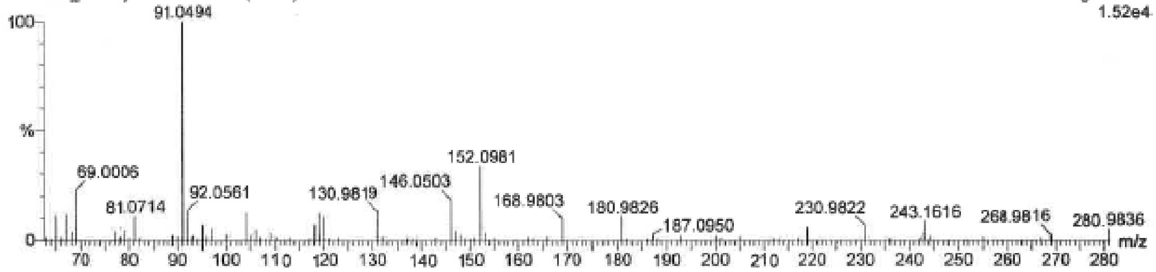

Mass	RA	Calc. Mass	mDa	PPM	DBE	i-FIT	Formula
Minimum:	0.10				-1.5		
Maximum:	100.00		5.0	10.0	50.0		
243.1616	8.79	243.1623	-0.7	-2.9	7.0	2773210.5	C16 H21 N O
200.1073	2.03	200.1075	-0.2	-1.0	7.5	2773045.3	C13 H14 N O
137.0841	0.54	137.0841	0.0	0.0	4.0	5546038.5	C8 H11 N O
123.0692	1.20	123.0684	0.8	6.5	4.0	5546070.0	C7 H9 N O

HRMS of compound **5f**:

Elemental Composition Report

Multiple Mass Analysis: 61 mass(es) processed - displaying only valid results
Tolerance = 10.0 PPM / DBE: min = -1.5, max = 50.0
Selected filters: None

Monoisotopic Mass, Odd and Even Electron Ions
498 formula(e) evaluated with 7 results within limits (all results (up to 1000) for each mass)
Elements Used:
C: 5-17 H: 5-23 N: 0-1 O: 0-2
Tere
2100895_06May21_A 122 (4.832)

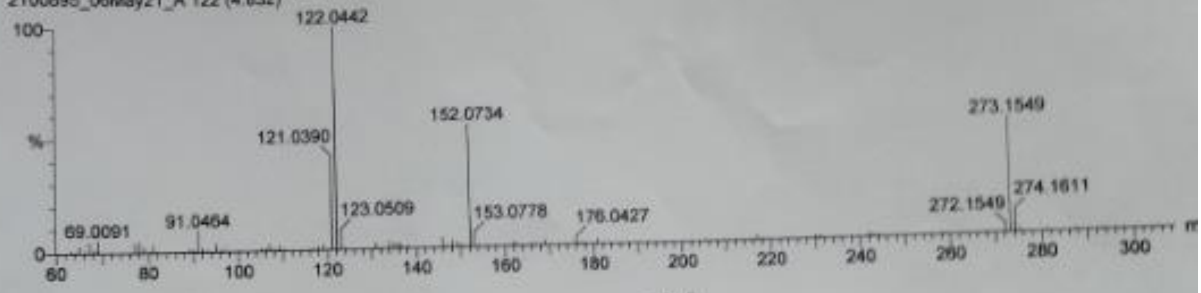

Mass	RA	Calc. Mass	mDa	PPM	DBE	Formula
Minimum:	15.00				-1.5	
Maximum:	100.00		5.0	10.0	50.0	
273.1725	20.53	273.1729	-0.4	-1.5	7.0	C17 H23 N O2
152.0846	22.52	152.0837	0.9	5.9	4.0	C9 H12 O2
152.0832	29.36	152.0837	-0.5	-3.3	4.0	C9 H12 O2
152.0720	52.99	152.0712	0.8	5.3	4.5	C8 H10 N O2
152.0706	49.16	152.0712	-0.6	-3.9	4.5	C8 H10 N O2
122.0374	33.17	122.0368	0.6	4.9	5.0	C7 H6 O2
122.0363	22.09	122.0368	-0.5	-4.1	5.0	C7 H6 O2

HRMS of compound **5g**:

Elemental Composition Report

Multiple Mass Analysis: 32 mass(es) processed - displaying only valid results
Tolerance = 10.0 PPM / DBE: min = -1.5, max = 50.0
Selected filters: None

Monoisotopic Mass, Odd and Even Electron Ions
533 formula(e) evaluated with 10 results within limits (all results (up to 1000) for each mass)
Elements Used:
C: 5-17 H: 5-20 N: 0-1 O: 0-1 F: 0-3

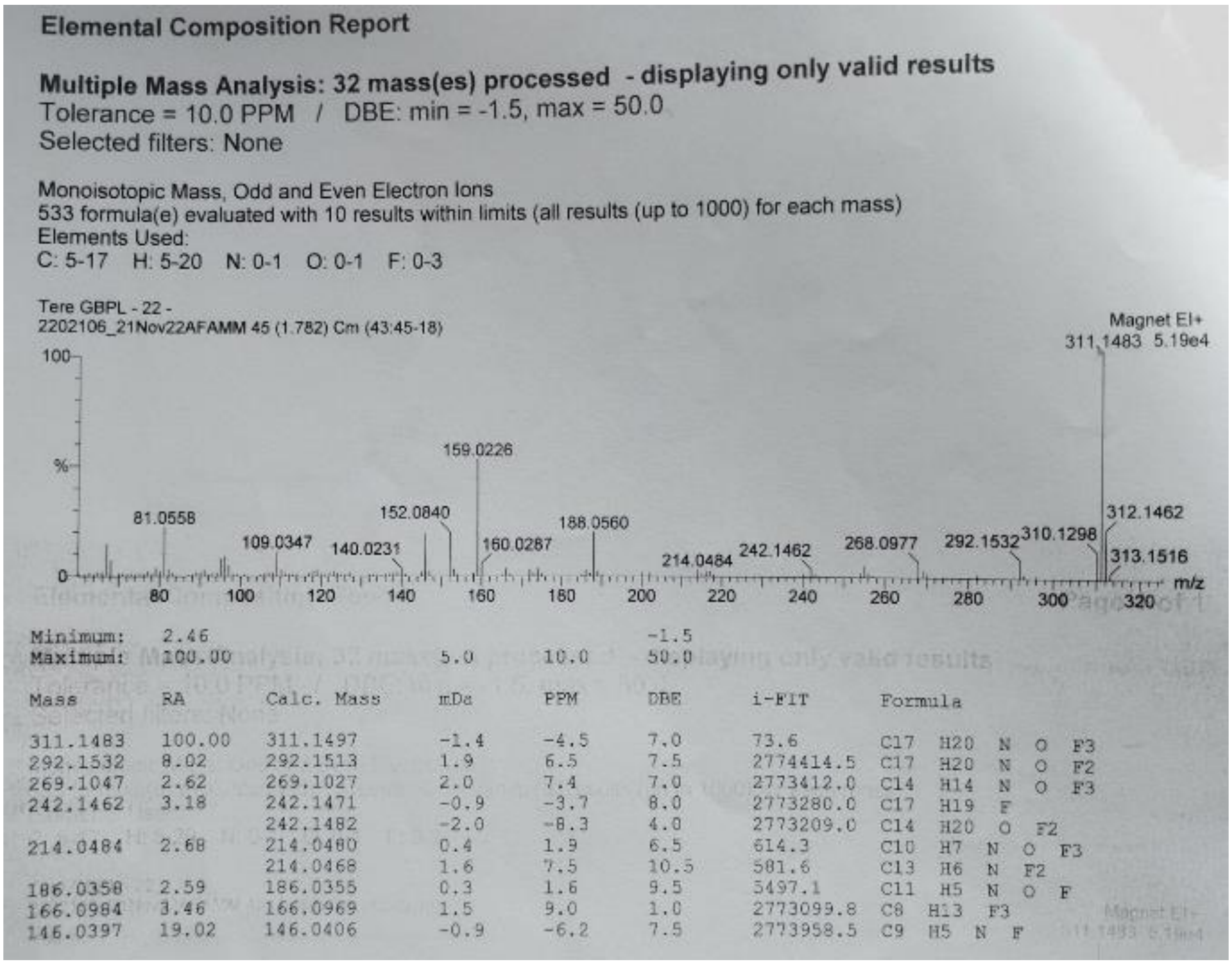

Minimum:		2.46				-1.5		
Maximum:		100.00		5.0	10.0	50.0		

Mass	RA	Calc. Mass	mDa	PPM	DBE	i-FIT	Formula
311.1483	100.00	311.1497	-1.4	-4.5	7.0	73.6	C17 H20 N O F3
292.1532	8.02	292.1513	1.9	6.5	7.5	2774414.5	C17 H20 N O F2
269.1047	2.62	269.1027	2.0	7.4	7.0	2773412.0	C14 H14 N O F3
242.1462	3.18	242.1471	-0.9	-3.7	8.0	2773280.0	C17 H19 F
		242.1482	-2.0	-8.3	4.0	2773209.0	C14 H20 O F2
214.0484	2.68	214.0480	0.4	1.9	6.5	614.3	C10 H7 N O F3
		214.0468	1.6	7.5	10.5	581.6	C13 H6 N F2
186.0358	2.59	186.0355	0.3	1.6	9.5	5497.1	C11 H5 N O F
166.0984	3.46	166.0969	1.5	9.0	1.0	2773099.8	C8 H13 F3
146.0397	19.02	146.0406	-0.9	-6.2	7.5	2773958.5	C9 H5 N F

HRMS of compound **5h**:

Elemental Composition Report

Multiple Mass Analysis: 170 mass(es) processed - displaying only valid results
Tolerance = 10.0 PPM / DBE: min = -1.5, max = 50.0
Selected filters: None

Monoisotopic Mass, Odd and Even Electron Ions
1108 formula(e) evaluated with 15 results within limits (all results (up to 1000) for each mass)
Elements Used:
C: 5-17 H: 5-20 N: 0-2 O: 0-1

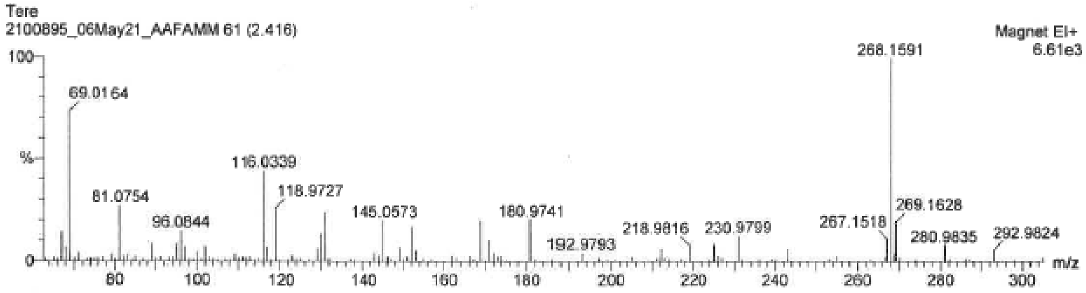

Minimum:	0.10				-1.5		
Maximum:	100.00		5.0	10.0	50.0		

Mass	RA	Calc. Mass	mDa	PPM	DBE	i-FIT	Formula
268.1591	100.00	268.1576	1.5	5.6	9.0	3.4	C17 H20 N2 O
267.1518	10.93	267.1497	2.1	7.9	9.5	3774.7	C17 H19 N2 O
253.1350	1.05	253.1341	0.9	3.6	9.5	5546046.0	C16 H17 N2 O
239.1175	0.67	239.1184	-0.9	-3.8	9.5	2773026.5	C15 H15 N2 O
226.1085	2.81	226.1106	-2.1	-9.3	9.0	2773015.5	C14 H14 N2 O
225.1011	8.12	225.1028	-1.7	-7.6	9.5	37.2	C14 H13 N2 O
214.0643	0.70	214.0657	-1.4	-6.5	13.5	5546036.0	C16 H8 N
197.0984	1.21	197.0966	1.8	9.1	8.5	2773056.5	C14 H13 O
146.0610	2.14	146.0606	0.4	2.7	6.5	5546062.5	C9 H8 N O
132.0452	0.71	132.0449	0.3	2.3	6.5	5546029.5	C8 H6 N O
110.0487	1.21	110.0480	0.7	6.4	4.0	2773027.5	C5 H6 N2 O
109.0884	3.35	109.0891	-0.7	-6.4	3.0	98.6	C7 H11 N
107.0729	0.60	107.0735	-0.6	-5.6	4.0	135.9	C7 H9 N
105.0570	0.61	105.0578	-0.8	-7.6	5.0	2773035.0	C7 H7 N
84.0945	0.96	84.0939	0.6	7.1	1.0	2773097.8	C6 H12

HRMS of compound **5i**:

Elemental Composition Report

Multiple Mass Analysis: 1855 mass(es) processed - displaying only valid results
Tolerance = 10.0 PPM / DBE: min = -1.5, max = 50.0
Selected filters: None

Monoisotopic Mass, Odd and Even Electron Ions
5847 formula(e) evaluated with 3 results within limits (all results (up to 1000) for each mass)
Elements Used:
C: 10-10 H: 14-14 N: 0-2 O: 0-1

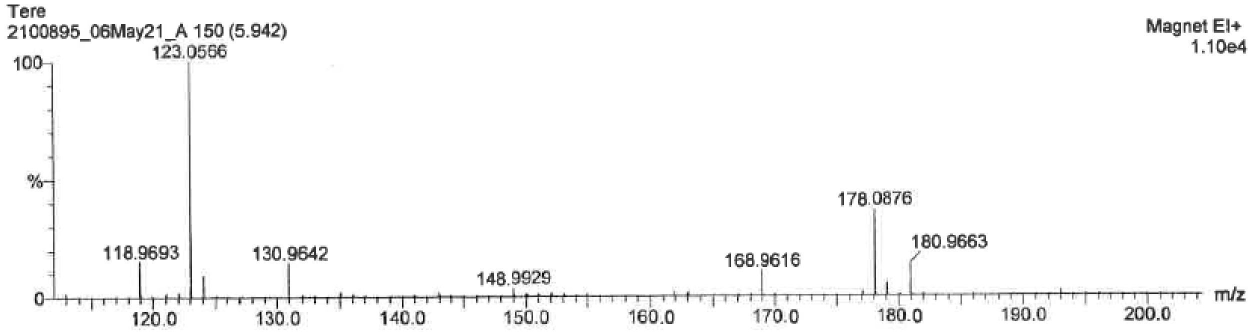

Minimum:	0.10				-1.5	
Maximum:	100.00		5.0	10.0	50.0	

Mass	RA	Calc. Mass	mDa	PPM	DBE	Formula
178.1123	0.11	178.1106	1.7	9.5	5.0	C10 H14 N2 O
178.1106	0.16	178.1106	0.0	0.0	5.0	C10 H14 N2 O
178.1090	0.39	178.1106	-1.6	-9.0	5.0	C10 H14 N2 O

HRMS of compound **5j**:

Elemental Composition Report

Multiple Mass Analysis: 1420 mass(es) processed - displaying only valid results
Tolerance = 10.0 PPM / DBE: min = -1.5, max = 50.0
Selected filters: None

Monoisotopic Mass, Odd and Even Electron Ions
14724 formula(e) evaluated with 189 results within limits (all results (up to 1000) for each mass)
Elements Used:
C: 5-10 H: 5-17 N: 0-3 O: 0-2

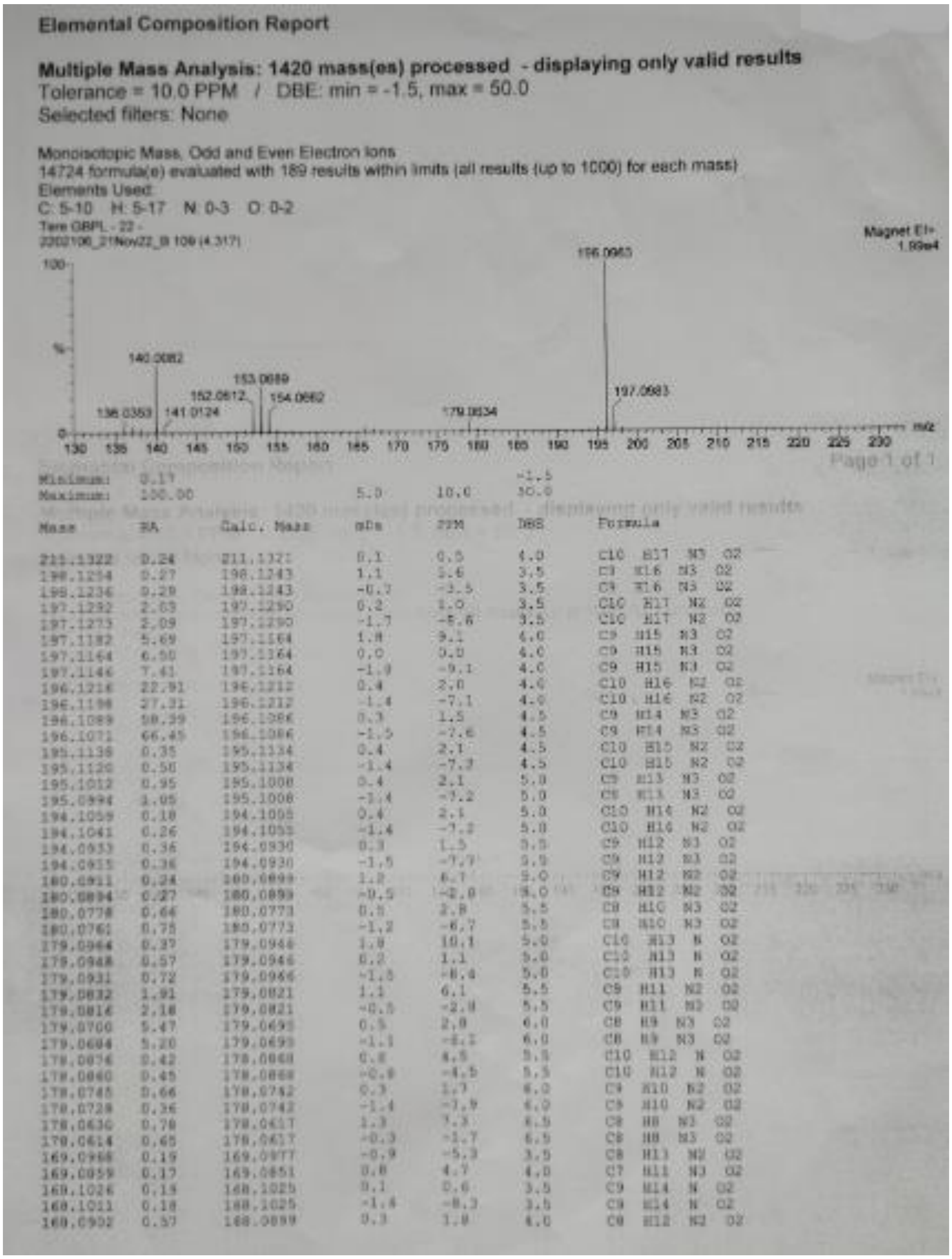

Page 1 of 1

Mass	RA	Calc. Mass	mDa	PPM	DBE	Formula
Minimum:	0.17				-1.5	
Maximum:	100.00		5.0	10.0	50.0	
211.1322	0.24	211.1321	0.1	0.5	4.0	C10 H17 N3 O2
198.1254	0.27	198.1243	1.1	5.6	3.5	C9 H16 N3 O2
198.1236	0.29	198.1243	-0.7	-3.5	3.5	C9 H16 N3 O2
197.1292	2.03	197.1290	0.2	1.0	3.5	C10 H17 N2 O2
197.1273	2.09	197.1290	-1.7	-8.6	3.5	C10 H17 N2 O2
197.1182	5.69	197.1164	1.8	9.1	4.0	C9 H15 N3 O2
197.1164	6.50	197.1164	0.0	0.0	4.0	C9 H15 N3 O2
197.1146	7.41	197.1164	-1.8	-9.1	4.0	C9 H15 N3 O2
196.1216	22.91	196.1212	0.4	2.0	4.0	C10 H16 N2 O2
196.1198	27.31	196.1212	-1.4	-7.1	4.0	C10 H16 N2 O2
196.1089	58.39	196.1086	0.3	1.5	4.5	C9 H14 N3 O2
196.1071	66.45	196.1086	-1.5	-7.6	4.5	C9 H14 N3 O2
195.1138	0.35	195.1134	0.4	2.1	4.5	C10 H15 N2 O2
195.1120	0.50	195.1134	-1.4	-7.2	4.5	C10 H15 N2 O2
195.1012	0.95	195.1008	0.4	2.1	5.0	C9 H13 N3 O2
195.0994	1.05	195.1008	-1.4	-7.2	5.0	C9 H13 N3 O2
194.1059	0.18	194.1055	0.4	2.1	5.0	C10 H14 N2 O2
194.1041	0.26	194.1055	-1.4	-7.2	5.0	C10 H14 N2 O2
194.0933	0.36	194.0930	0.3	1.5	5.5	C9 H12 N3 O2
194.0915	0.36	194.0930	-1.5	-7.7	5.5	C9 H12 N3 O2
180.0911	0.24	180.0899	1.2	6.7	5.0	C9 H12 N2 O2
180.0894	0.27	180.0899	-0.5	-2.8	5.0	C9 H12 N2 O2
180.0778	0.64	180.0773	0.5	2.8	5.5	C8 H10 N3 O2
180.0761	0.75	180.0773	-1.2	-6.7	5.5	C8 H10 N3 O2
179.0964	0.37	179.0946	1.8	10.1	5.0	C10 H13 N O2
179.0948	0.57	179.0946	0.2	1.1	5.0	C10 H13 N O2
179.0931	0.72	179.0946	-1.5	-8.4	5.0	C10 H13 N O2
179.0832	1.91	179.0821	1.1	6.1	5.5	C9 H11 N2 O2
179.0816	2.18	179.0821	-0.5	-2.8	5.5	C9 H11 N2 O2
179.0700	5.47	179.0695	0.5	2.8	6.0	C8 H9 N3 O2
179.0684	5.20	179.0695	-1.1	-6.1	6.0	C8 H9 N3 O2
178.0876	0.42	178.0868	0.8	4.5	5.5	C10 H12 N O2
178.0860	0.45	178.0868	-0.8	-4.5	5.5	C10 H12 N O2
178.0745	0.66	178.0742	0.3	1.7	6.0	C9 H10 N2 O2
178.0728	0.36	178.0742	-1.4	-7.9	6.0	C9 H10 N2 O2
178.0630	0.78	178.0617	1.3	7.3	6.5	C8 H8 N3 O2
178.0614	0.65	178.0617	-0.3	-1.7	6.5	C8 H8 N3 O2
169.0968	0.19	169.0977	-0.9	-5.3	3.5	C8 H13 N2 O2
169.0859	0.17	169.0851	0.8	4.7	4.0	C7 H11 N3 O2
168.1026	0.19	168.1025	0.1	0.6	3.5	C9 H14 N O2
168.1011	0.18	168.1025	-1.4	-8.3	3.5	C9 H14 N O2
168.0902	0.57	168.0899	0.3	1.8	4.0	C8 H12 N2 O2

HRMS of compound **5k**:

Elemental Composition Report

Multiple Mass Analysis: 387 mass(es) processed - displaying only valid results
Tolerance = 10.0 PPM / DBE: min = -1.5, max = 50.0
Selected filters: None

Monoisotopic Mass, Odd and Even Electron Ions
3304 formula(e) evaluated with 72 results within limits (all results (up to 1000) for each mass)
Elements Used:
C: 5-12 H: 5-19 N: 0-1 O: 0-3
Tele GBPL - 22 -
2202106_21Nov22 125 (4.951)

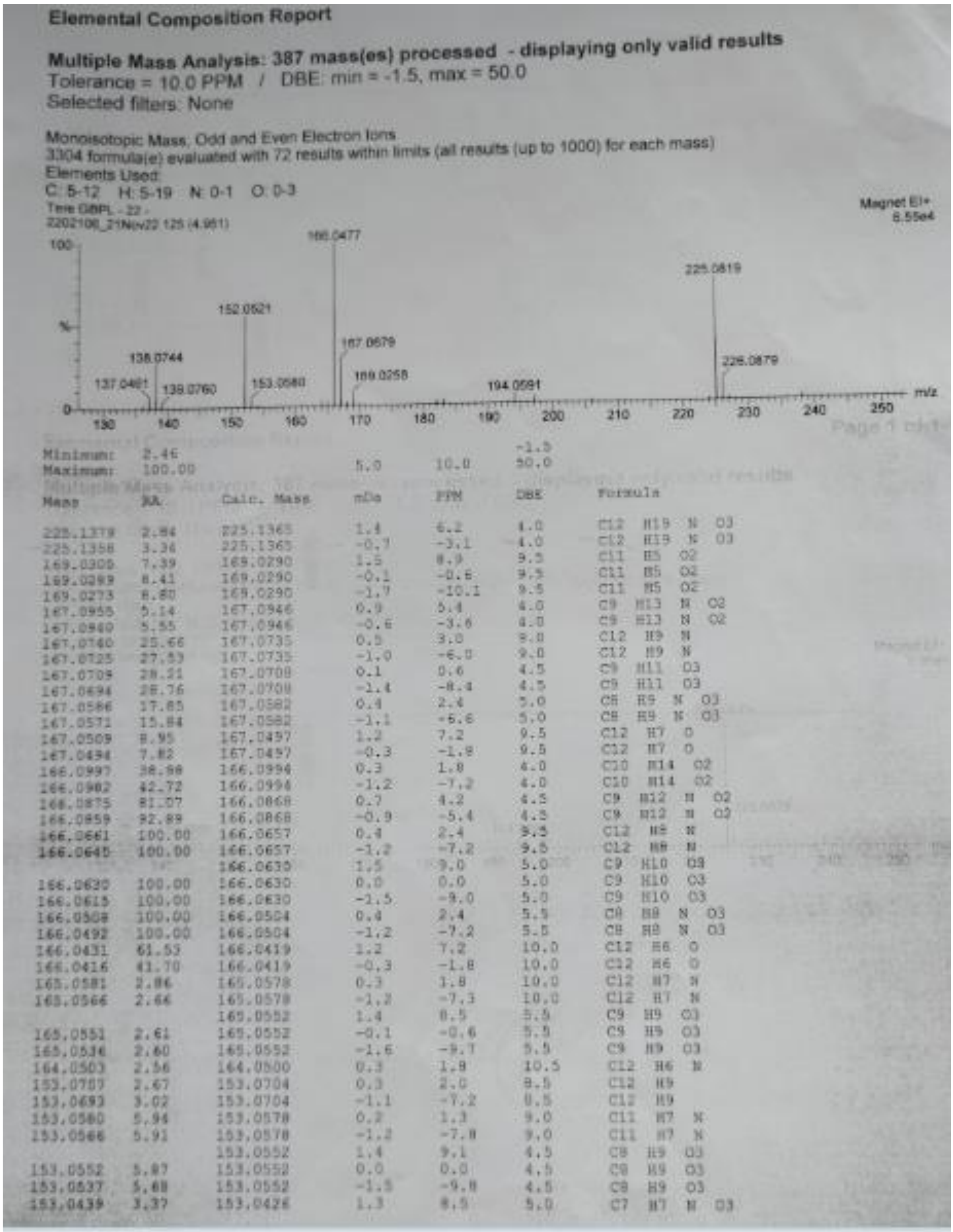

					-1.5	
Minimum:	2.46					
Maximum:	100.00		5.0	10.0	50.0	
Mass	RA	Calc. Mass	mDa	PPM	DBE	Formula
225.1379	2.84	225.1365	1.4	6.2	4.0	C12 H19 N O3
225.1358	3.36	225.1365	-0.7	-3.1	4.0	C12 H19 N O3
169.0305	7.39	169.0290	1.5	8.9	9.5	C11 H5 O2
169.0289	8.41	169.0290	-0.1	-0.6	9.5	C11 H5 O2
169.0273	8.80	169.0290	-1.7	-10.1	9.5	C11 H5 O2
167.0955	5.14	167.0946	0.9	5.4	4.0	C9 H13 N O2
167.0940	5.55	167.0946	-0.6	-3.6	4.0	C9 H13 N O2
167.0740	25.66	167.0735	0.5	3.0	9.0	C12 H9 N
167.0725	27.53	167.0735	-1.0	-6.0	9.0	C12 H9 N
167.0709	28.21	167.0708	0.1	0.6	4.5	C9 H11 O3
167.0694	28.76	167.0708	-1.4	-8.4	4.5	C9 H11 O3
167.0586	17.85	167.0582	0.4	2.4	5.0	C8 H9 N O3
167.0571	15.84	167.0582	-1.1	-6.6	5.0	C8 H9 N O3
167.0509	8.95	167.0497	1.2	7.2	9.5	C12 H7 O
167.0494	7.82	167.0497	-0.3	-1.8	9.5	C12 H7 O
166.0997	38.98	166.0994	0.3	1.8	4.0	C10 H14 O2
166.0982	42.72	166.0994	-1.2	-7.2	4.0	C10 H14 O2
166.0875	81.07	166.0868	0.7	4.2	4.5	C9 H12 N O2
166.0859	92.89	166.0868	-0.9	-5.4	4.5	C9 H12 N O2
166.0661	100.00	166.0657	0.4	2.4	9.5	C12 H8 N
166.0645	100.00	166.0657	-1.2	-7.2	9.5	C12 H8 N
		166.0630	1.5	9.0	5.0	C9 H10 O3
166.0630	100.00	166.0630	0.0	0.0	5.0	C9 H10 O3
166.0615	100.00	166.0630	-1.5	-9.0	5.0	C9 H10 O3
166.0508	100.00	166.0504	0.4	2.4	5.5	C8 H8 N O3
166.0492	100.00	166.0504	-1.2	-7.2	5.5	C8 H8 N O3
166.0431	61.53	166.0419	1.2	7.2	10.0	C12 H6 O
166.0416	41.70	166.0419	-0.3	-1.8	10.0	C12 H6 O
165.0581	2.86	165.0578	0.3	1.8	10.0	C12 H7 N
165.0566	2.64	165.0578	-1.2	-7.3	10.0	C12 H7 N
		165.0552	1.4	8.5	5.5	C9 H9 O3
165.0551	2.61	165.0552	-0.1	-0.6	5.5	C9 H9 O3
165.0536	2.60	165.0552	-1.6	-9.7	5.5	C9 H9 O3
164.0503	2.56	164.0500	0.3	1.8	10.5	C12 H6 N
153.0707	2.67	153.0704	0.3	2.0	8.5	C12 H9
153.0693	3.02	153.0704	-1.1	-7.2	8.5	C12 H9
153.0580	5.94	153.0578	0.2	1.3	9.0	C11 H7 N
153.0566	5.91	153.0578	-1.2	-7.8	9.0	C11 H7 N
		153.0552	1.4	9.1	4.5	C8 H9 O3
153.0552	5.87	153.0552	0.0	0.0	4.5	C8 H9 O3
153.0537	5.68	153.0552	-1.5	-9.8	4.5	C8 H9 O3
153.0439	3.37	153.0426	1.3	8.5	5.0	C7 H7 N O3

HRMS of compound **6**:

Elemental Composition Report

Multiple Mass Analysis: 3391 mass(es) processed - displaying only valid results
Tolerance = 10.0 PPM / DBE: min = -1.5, max = 50.0
Selected filters: None

Monoisotopic Mass, Odd and Even Electron Ions
12148 formula(e) evaluated with 154 results within limits (all results (up to 1000) for each mass)
Elements Used:
C: 3-8 H: 5-15 N: 0-1 O: 0-1
2101418 07Jul21_B 60 (2.376)
Magnet EI+

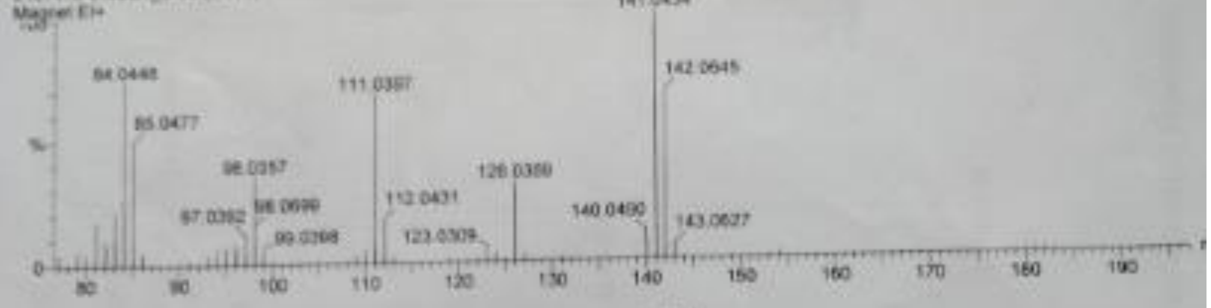

Minimum:	0.10				-1.5	
Maximum:	300.00		5.0	10.0	50.0	
Mass	RA	Calc. Mass	mDa	PPM	DBE	Formula
141.1161	0.62	141.1154	0.7	5.0	2.0	C8 H15 N O
141.1148	0.70	141.1154	-0.6	-4.3	2.0	C8 H15 N O
126.1050	0.10	126.1045	0.5	4.0	2.0	C8 H14 O
126.0911	0.16	126.0919	-0.8	-6.3	2.5	C7 H12 N O
124.0772	0.10	124.0762	1.0	8.1	3.5	C7 H10 N O
123.0694	0.12	123.0684	1.0	8.1	4.0	C7 H9 N O
123.0683	0.13	123.0684	-0.1	-0.8	4.0	C7 H9 N O
121.0539	0.10	121.0528	1.1	9.1	5.0	C7 H7 N O
121.0528	0.12	121.0528	0.0	0.0	5.0	C7 H7 N O
121.0517	0.24	121.0528	-1.1	-9.1	5.0	C7 H7 N O
119.0378	0.21	119.0371	0.7	5.9	6.0	C7 H5 N O
119.0367	0.13	119.0371	-0.4	-3.4	6.0	C7 H5 N O
113.0961	0.10	113.0966	-0.5	-4.4	1.5	C7 H13 O
113.0847	0.87	113.0841	0.6	5.3	2.0	C6 H11 N O
113.0836	0.39	113.0841	-0.5	-4.4	2.0	C6 H11 N O
112.0894	0.13	112.0888	0.6	5.4	2.0	C7 H12 O
112.0884	0.11	112.0888	-0.4	-3.6	2.0	C7 H12 O
112.0770	1.04	112.0762	0.8	7.1	2.5	C6 H10 N O
112.0760	1.08	112.0762	-0.2	-1.8	2.5	C6 H10 N O
111.1173	0.11	111.1174	-0.1	-0.9	1.5	C8 H15
111.1050	0.20	111.1048	0.2	1.8	2.0	C7 H13 N
111.1040	0.20	111.1048	-0.8	-7.2	2.0	C7 H13 N
111.0815	0.32	111.0810	0.5	4.5	2.5	C7 H11 O
111.0805	0.35	111.0810	-0.5	-4.5	2.5	C7 H11 O
111.0693	0.50	111.0684	0.9	8.1	3.0	C6 H9 N O
111.0683	0.62	111.0684	-0.1	-0.9	3.0	C6 H9 N O
110.0615	0.25	110.0606	0.9	8.2	3.5	C6 H8 N O
110.0605	0.23	110.0606	-0.1	-0.9	3.5	C6 H8 N O
110.0595	0.25	110.0606	-1.1	-10.0	3.5	C6 H8 N O
109.0650	0.15	109.0653	-0.3	-2.8	3.5	C7 H9 O
109.0530	0.14	109.0528	0.2	1.8	4.0	C6 H7 N O
109.0520	0.15	109.0528	-0.8	-7.3	4.0	C6 H7 N O
108.0567	0.12	108.0575	-0.8	-7.4	4.0	C7 H8 O
108.0458	0.51	108.0449	0.9	8.3	4.5	C6 H6 N O
108.0448	0.66	108.0449	-0.1	-0.9	4.5	C6 H6 N O
107.0499	0.13	107.0497	0.2	1.9	4.5	C7 H7 O
107.0489	0.12	107.0497	-0.8	-7.5	4.5	C7 H7 O
107.0381	0.14	107.0371	1.0	9.3	5.0	C6 H5 N O
107.0371	0.17	107.0371	0.0	0.0	5.0	C6 H5 N O
107.0361	0.20	107.0371	-1.0	-9.3	5.0	C6 H5 N O
106.0429	0.20	106.0419	1.0	9.4	5.0	C7 H6 O
106.0419	0.17	106.0419	0.0	0.0	5.0	C7 H6 O

HRMS of compound **6a**:

Elemental Composition Report

Tolerance = 5.0 PPM / DBE: min = -1.5, max = 200.0
Element prediction: Off
Number of isotope peaks used for i-FIT = 3

Monoisotopic Mass, Even Electron Ions
415 formula(e) evaluated with 2 results within limits (all results (up to 1000) for each mass)
Elements Used:
C: 0-32 H: 0-40 N: 0-6 O: 0-7 Na: 0-2
20240625_ P6BL 24-1 73 (3.167)
1: TOF MS ES+

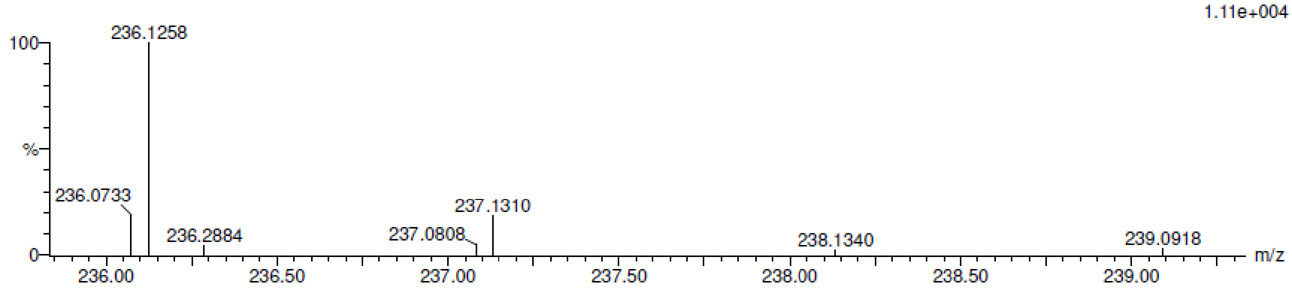

Minimum:	36.00				-1.5			
Maximum:	100.00		5.0	5.0	200.0			
Mass	RA	Calc. Mass	mDa	PPM	DBE	i-FIT	i-FIT (Norm)	Formula
236.1258	100.00	236.1263	-0.5	-2.1	2.5	42.6	0.0	C11 H19 N O3 Na
		236.1246	1.2	5.1	1.5	45.7	3.1	C8 H18 N3 O5

HRMS of compound **6b**:

Elemental Composition Report

Tolerance = 5.0 PPM / DBE: min = -1.5, max = 200.0
Element prediction: Off
Number of isotope peaks used for i-FIT = 3

Monoisotopic Mass, Even Electron Ions
388 formula(e) evaluated with 1 results within limits (all results (up to 1000) for each mass)
Elements Used:
C: 0-32 H: 0-40 N: 0-6 O: 0-7 Na: 0-2
20240625_ P6BL 24-2 39 (1.305)
2: TOF MS ES+

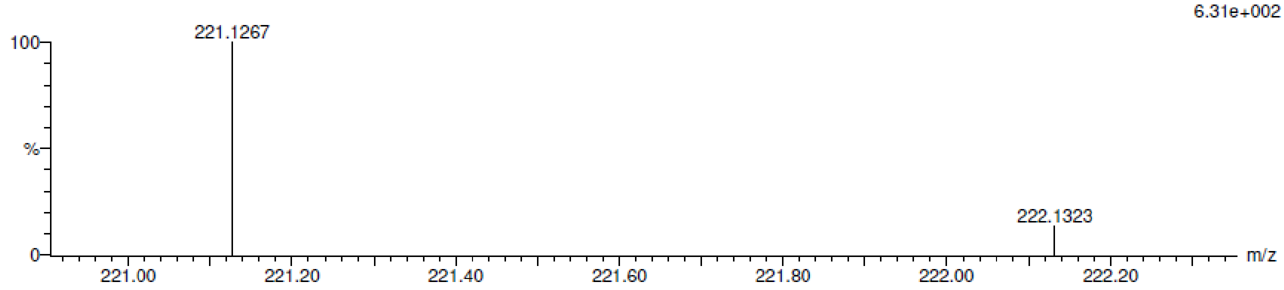

Minimum:	36.00				-1.5			
Maximum:	100.00		5.0	5.0	200.0			
Mass	RA	Calc. Mass	mDa	PPM	DBE	i-FIT	i-FIT (Norm)	Formula
221.1267	100.00	221.1266	0.1	0.5	2.5	13.4	0.0	C10 H18 N2 O2 Na

HPLC conditions and HPLC spectral data:

General conditions

Column: Ascentis® C18 10cm x 4.6 mm, 3 μM (Supelco)

Mobile phase: Water / acetonitrile (ACN) mixtures + 0.05% formic acid.

Flow rate: Isocratic 1 ml / min (except for compound **6b**, which was 1.5 ml/min)

All solvents were HPLC grade, and the mixtures were degassed by sonication before use.

Detection wavelength was 214 nm

Injection volume 20 μL

Column temperature: Ambient

Gabapentin **1** (water:ACN; 96:4)

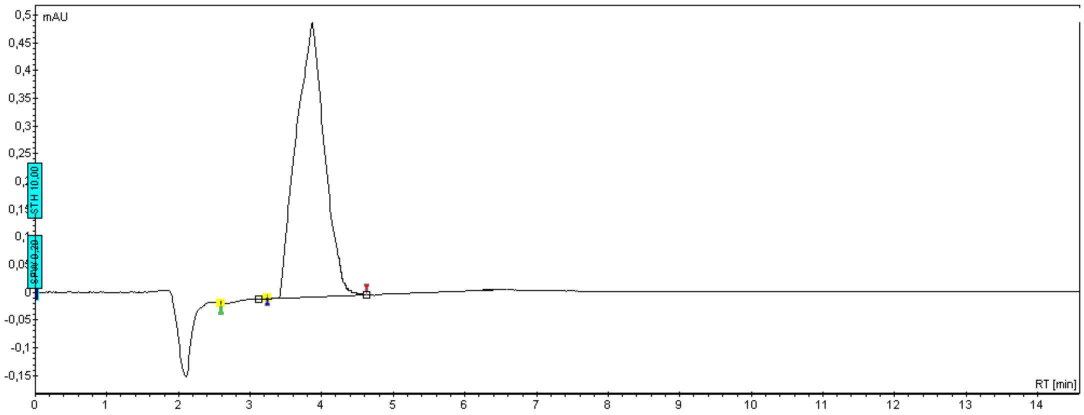

#	Start (min)	Peak (min)	End (min)	% Area
1	2.587	2.667	3.240	0.54
2	3.240	3.867	4.668	**99.46**

5 (water: ACN 70:30)

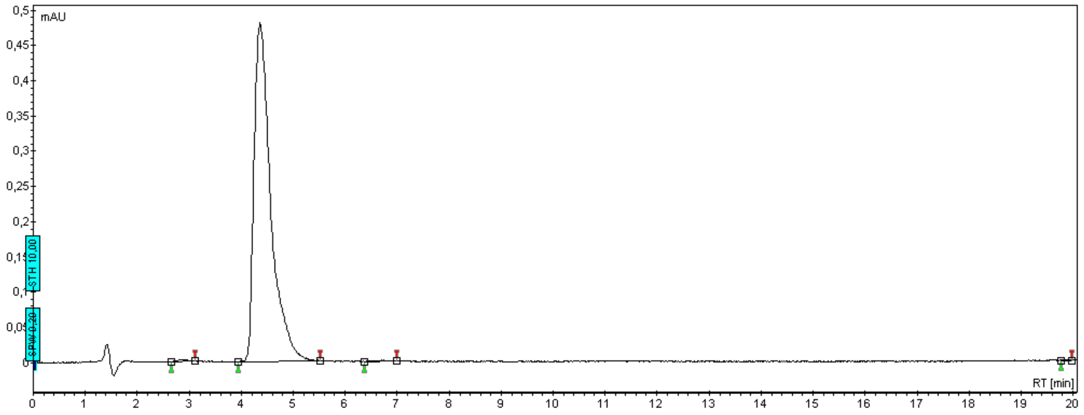

#	Start (min)	Peak (min)	End (min)	% Area
1	2.653	2.880	3.106	0.30
2	3.933	4.360	5.520	**99.45**
3	6.373	6.666	6.999	0.21
4	19.759	19.865	19.972	0.04

5b (water:ACN; 60:40)

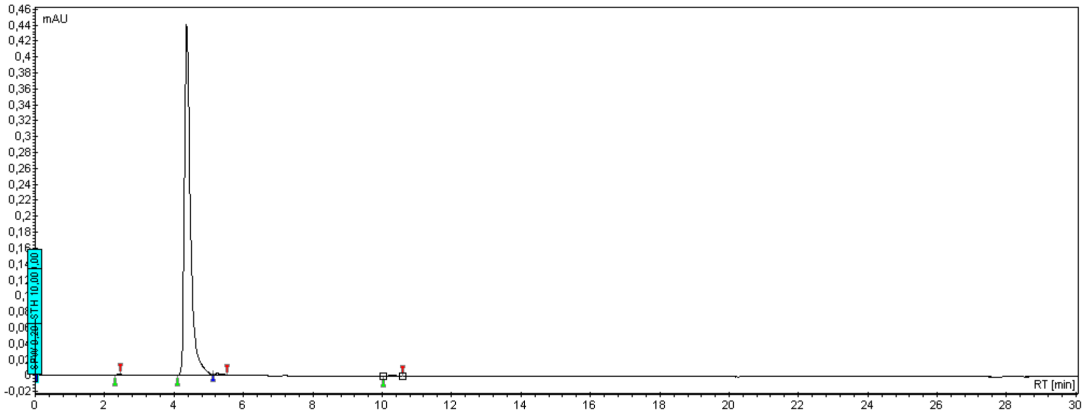

#	Start (min)	Peak (min)	End (min)	% Area
1	2.306	2.413	2.466	0.22
2	4.093	4.360	5.120	**98.54**
3	5.120	5.253	5.533	0.89
4	10.026	10.293	10.586	0.35

5c (water:ACN; 60:40)

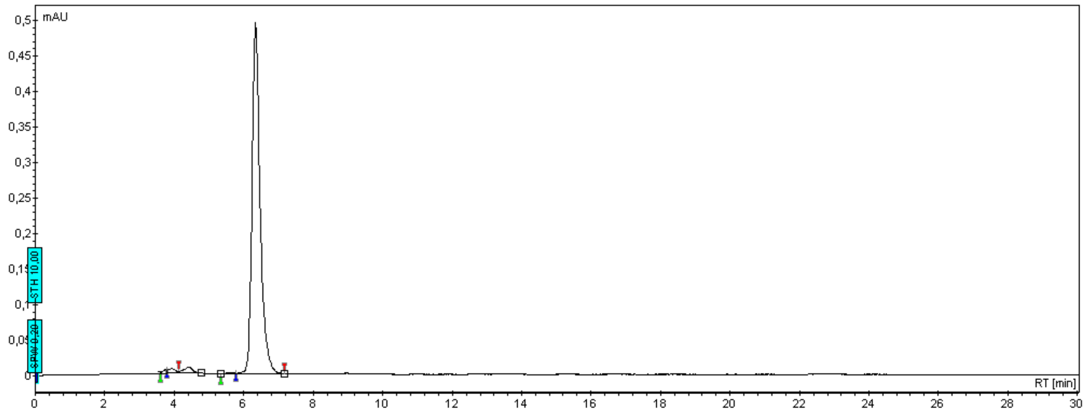

#	Start (min)	Peak (min)	End (min)	% Area
1	3.600	3.746	3.800	0.63
2	3.800	3.920	4.133	1.22
3	5.346	5.600	5.773	0.28
4	5.773	6.333	7.173	**97.87**

5d (water:ACN; 60:40)

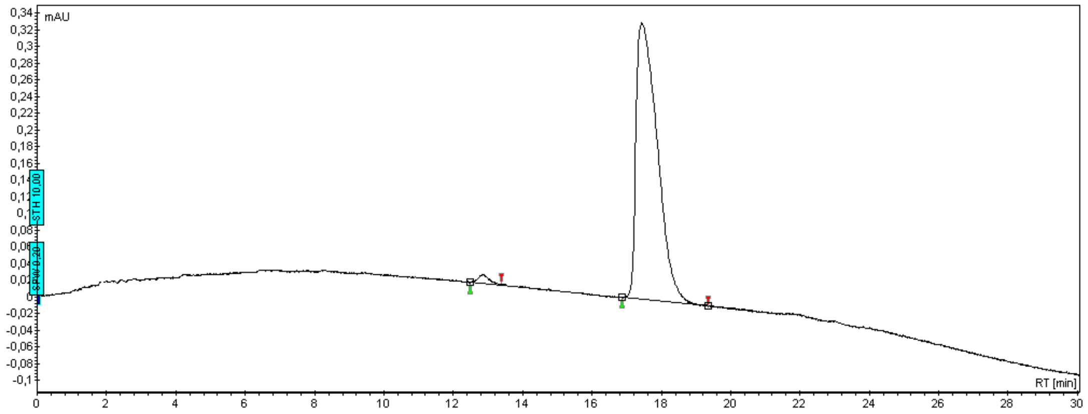

#	Start (min)	Peak (min)	End (min)	% Area
1	12.492	12.866	13.386	1.66
2	16.879	17.452	19.359	**98.34**

5f (water:ACN; 60:40)

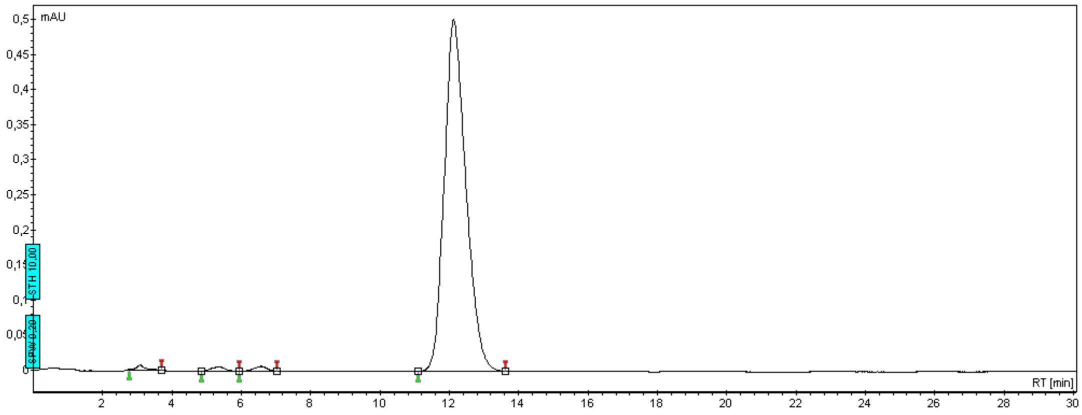

#	Start (min)	Peak (min)	End (min)	% Area
1	2.760	3.080	3.706	0.76
2	4.840	5.360	5.946	0.72
3	5.946	6.573	7.039	0.76
4	11.093	12.119	13.626	**97.76**

5g (water:ACN; 60:40)

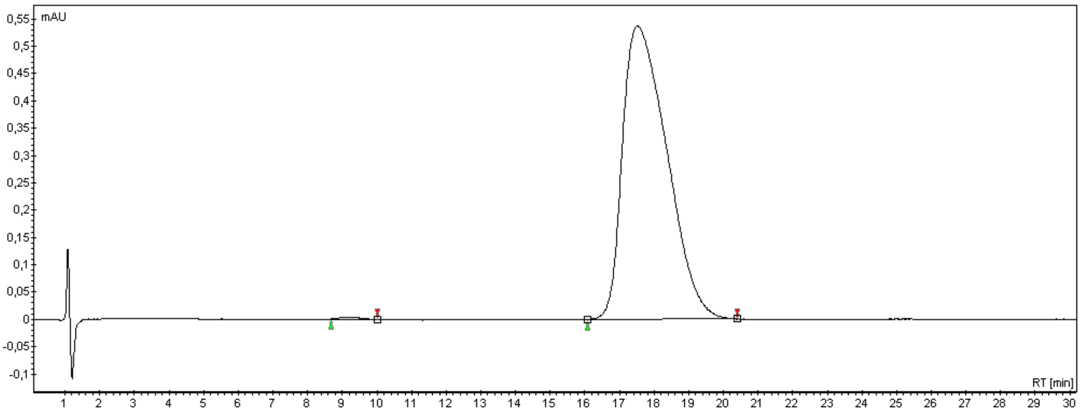

#	Start (min)	Peak (min)	End (min)	% Area
1	8.679	9.226	10.013	0.41
2	16.092	17.519	20.399	**99.59**

5h (water: ACN; 60:40)

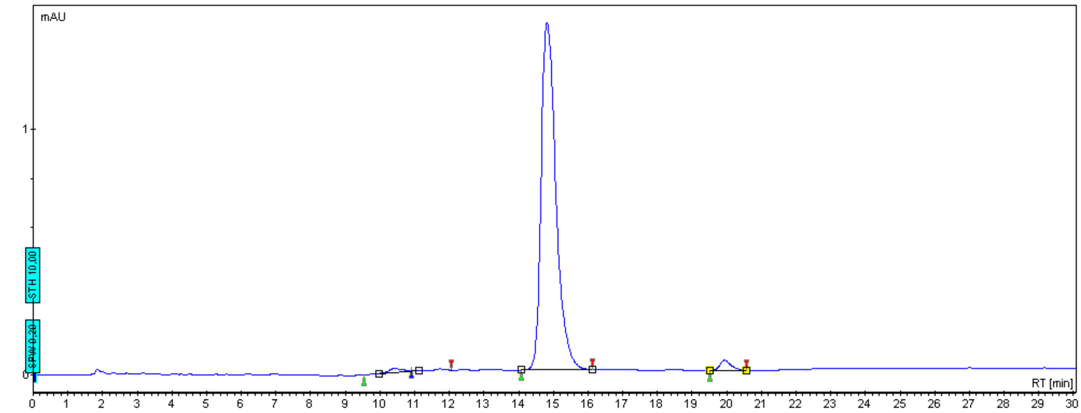

#	Start (min)	Peak (min)	End (min)	% Area
1	9.540	10.399	10.907	0.88
2	10.907	11.866	12.066	0.26
3	14.066	14.812	16.119	**96.73**
4	19.505	19.945	20.572	2.13

5i (water: ACN; 70:30)

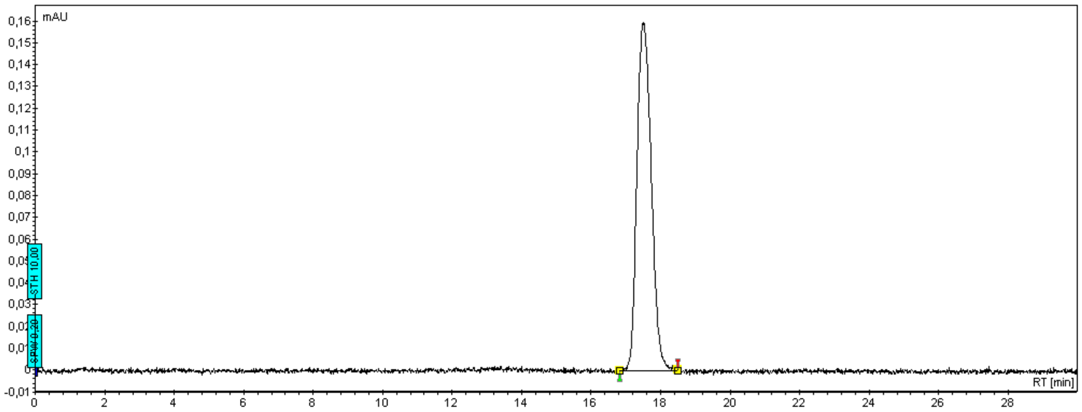

#	Start (min)	Peak (min)	End (min)	% Area
1	16.825	17.505	18.505	**100**

5j (water:ACN; 85:15)

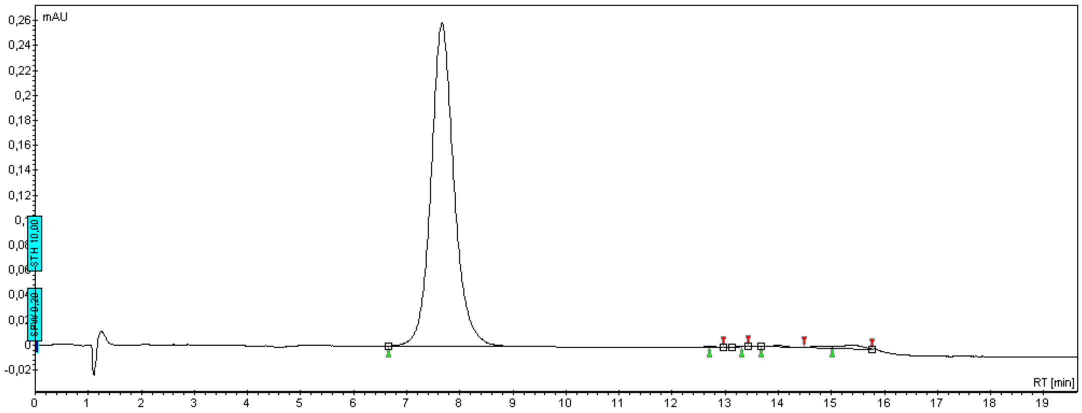

#	Start (min)	Peak (min)	End (min)	% Area
1	6.653	7.666	9.346	**98.53**
2	12.692	12.746	12.959	0.09
3	13306	13.406	13.439	0.03
4	13.679	13.999	14.492	0.34
5	15.012	15.412	15.772	1.01

6a (water:ACN; 85:15)

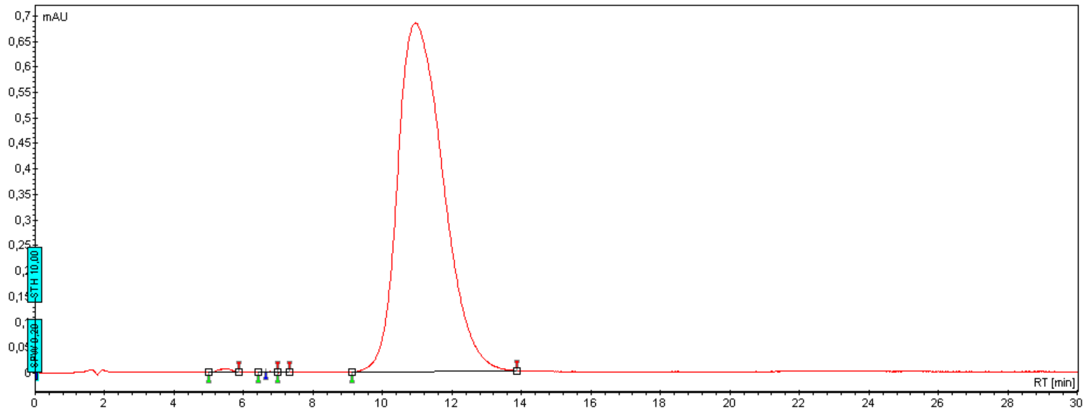

#	Start (min)	Peak (min)	End (min)	% Area
1	5.000	5.533	5.853	0.26
2	6.413	6.640	6.640	0.014
3	6.640	6.773	6.973	0.034
4	6.986	7.133	7.333	0.019
5	9.133	10.946	13.852	**99.67**

6b (water:ACN; 85:15)

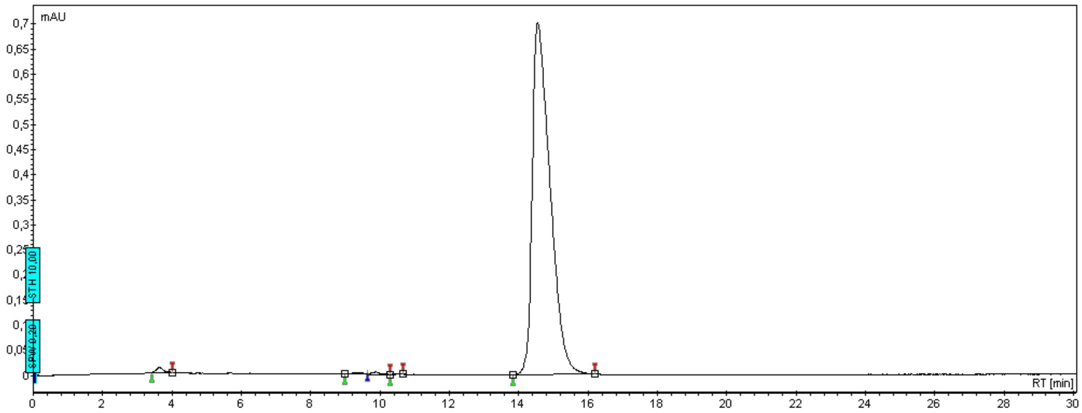

#	Start (min)	Peak (min)	End (min)	% Area
1	3.426	3.640	4.013	0.58
2	8.999	9.373	9.626	0.21
3	9.626	9.866	10.293	0.28
4	10.293	10.946	13.852	0.03
5	13.826	14.546	16.212	**98.90**

Monitoring of the lactamization reaction of 1 to 5:

(a) ^1H NMR (CD_3OD)

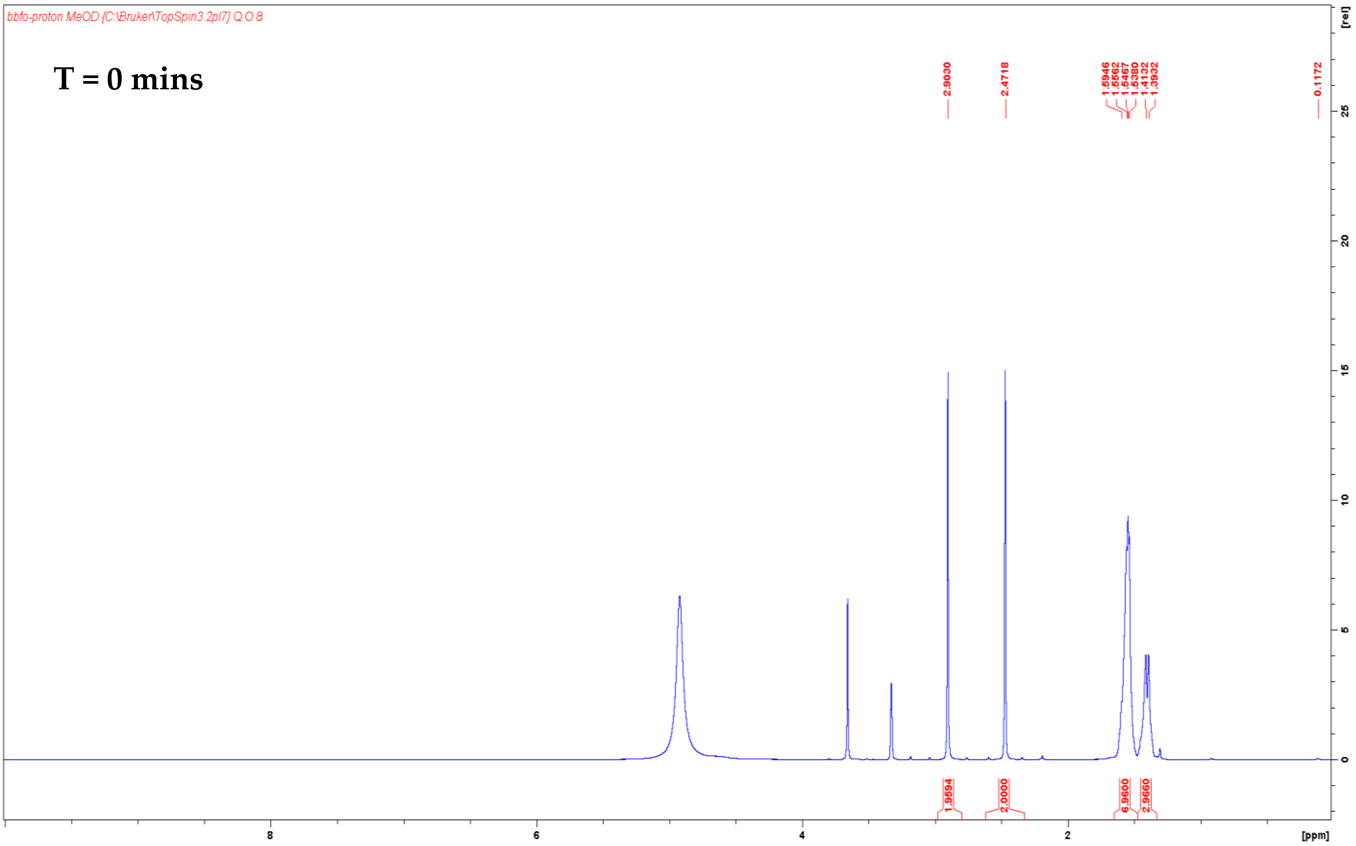

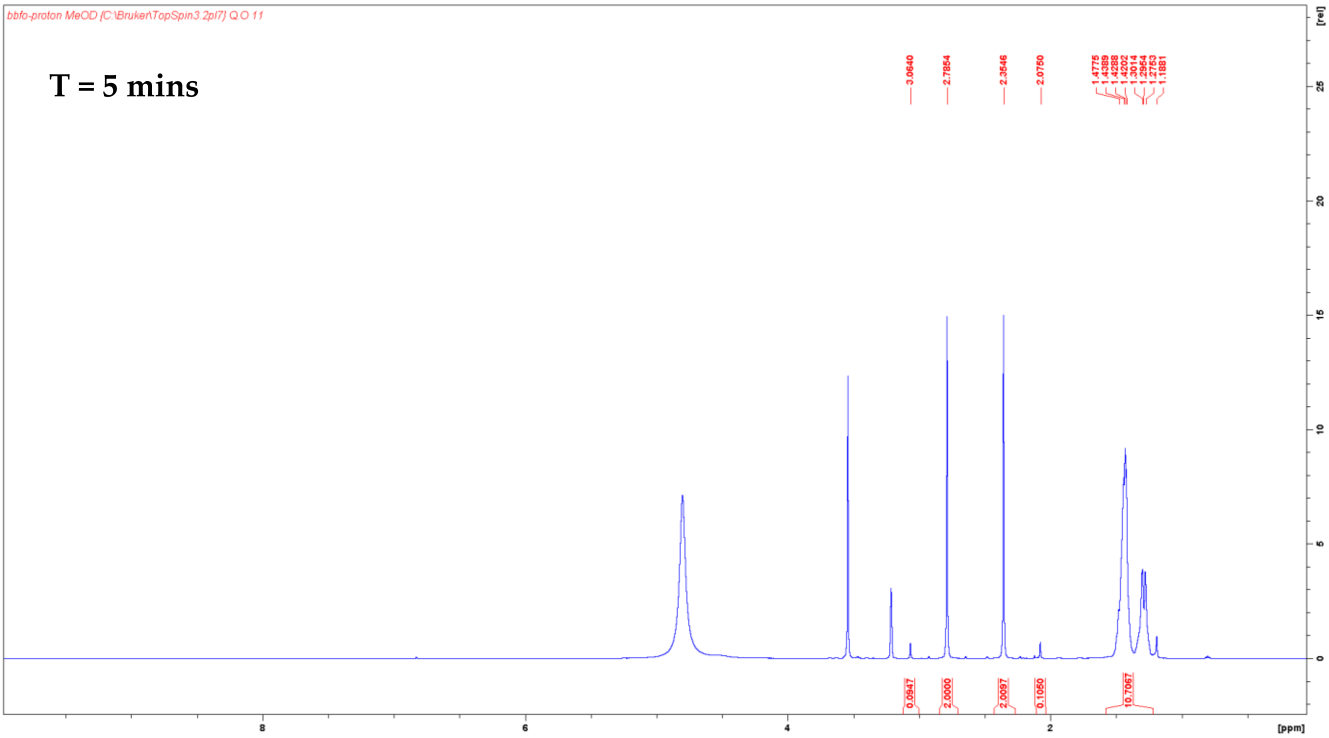

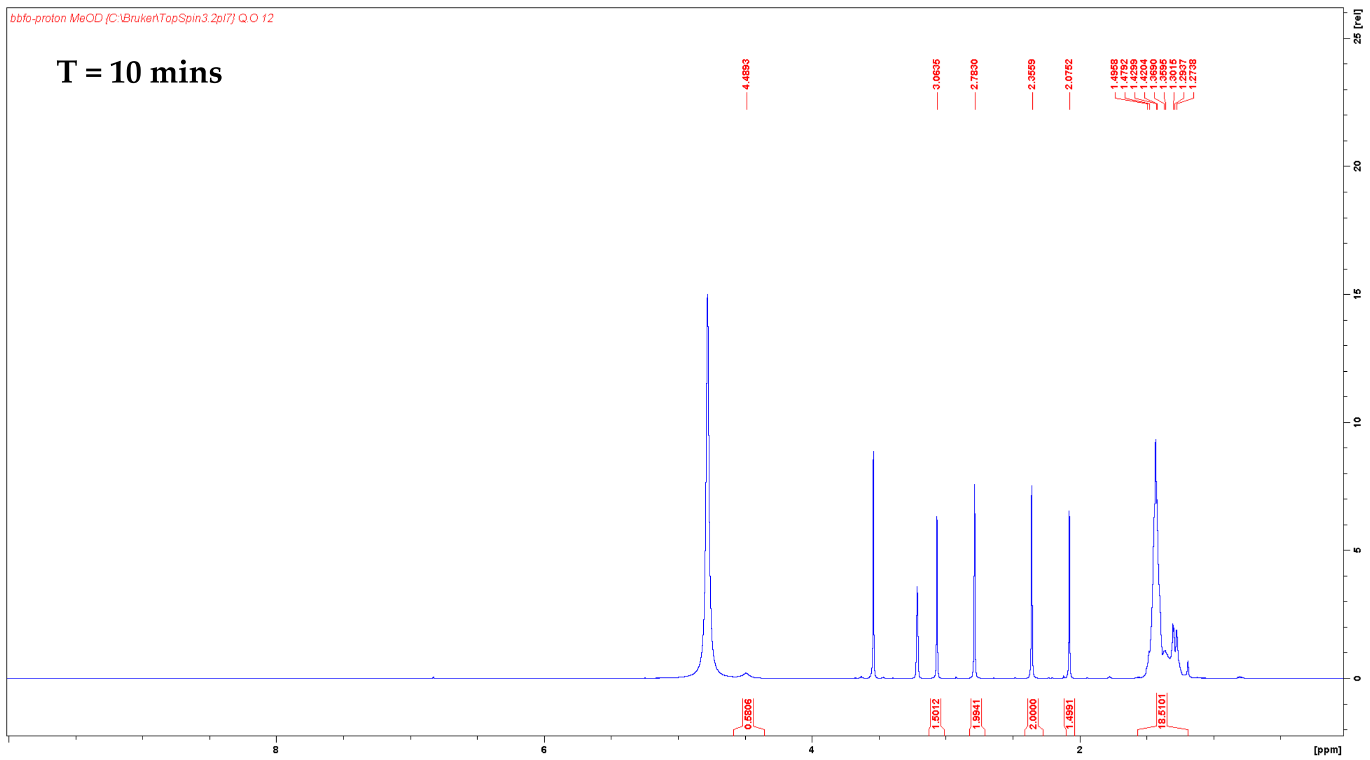
bbfo-proton MeOD {C:\Bruker\TopSpin3.2pl7} Q.O 12
T = 10 mins
4.4893
3.0635
2.7830
2.3559
2.0752
1.4958
1.4792
1.4299
1.4204
1.3690
1.3595
1.3015
1.2937
1.2738
0.5806
1.5012
1.9941
2.0000
1.4991
18.5101
[ppm]
[rel]

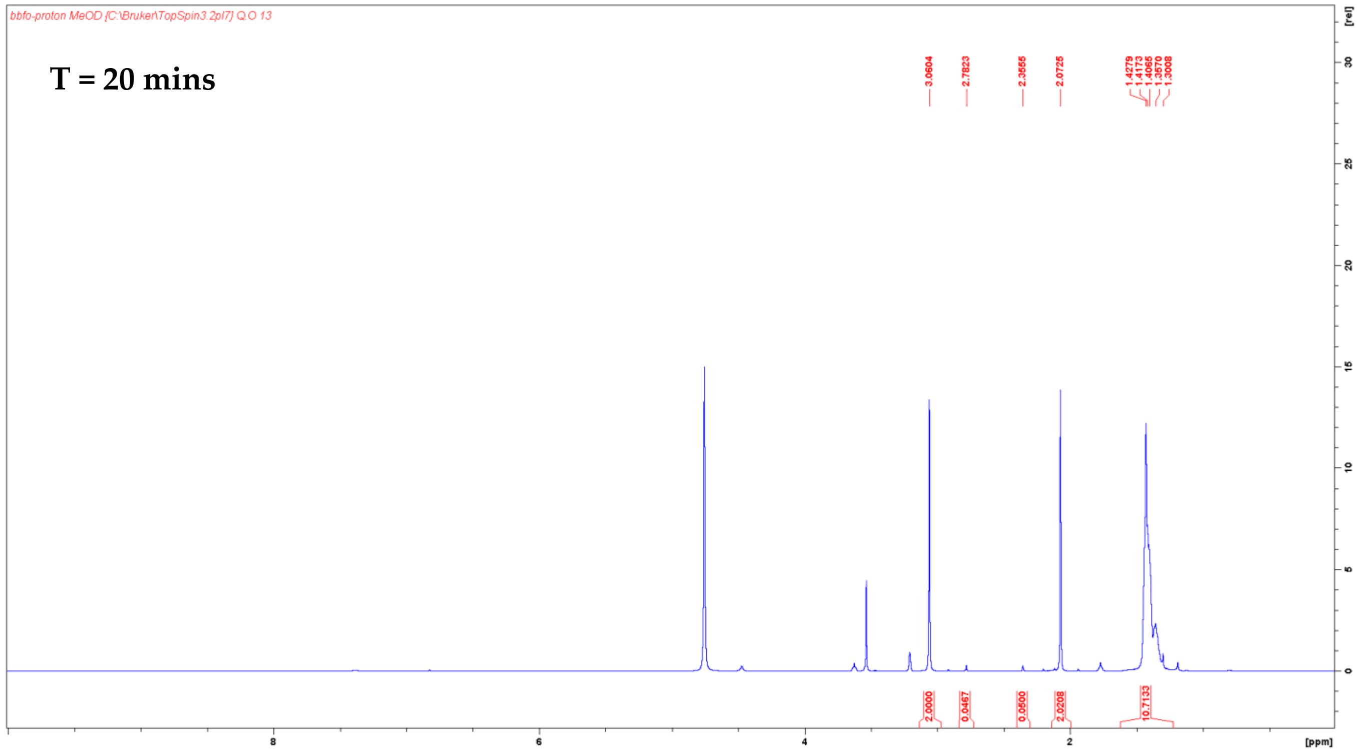
bbfo-proton MeOD {C:\Bruker\TopSpin3.2pl7} Q.O 13
T = 20 mins
3.0604
2.7823
2.3556
2.0725
1.4279
1.4173
1.4066
1.3670
1.3008
2.0000
0.0467
0.0500
2.0208
10.7133
[ppm]
[rel]

Overlay of all ^1H NMR spectra (500 MHz, CD_3OD) at different times during the reaction:

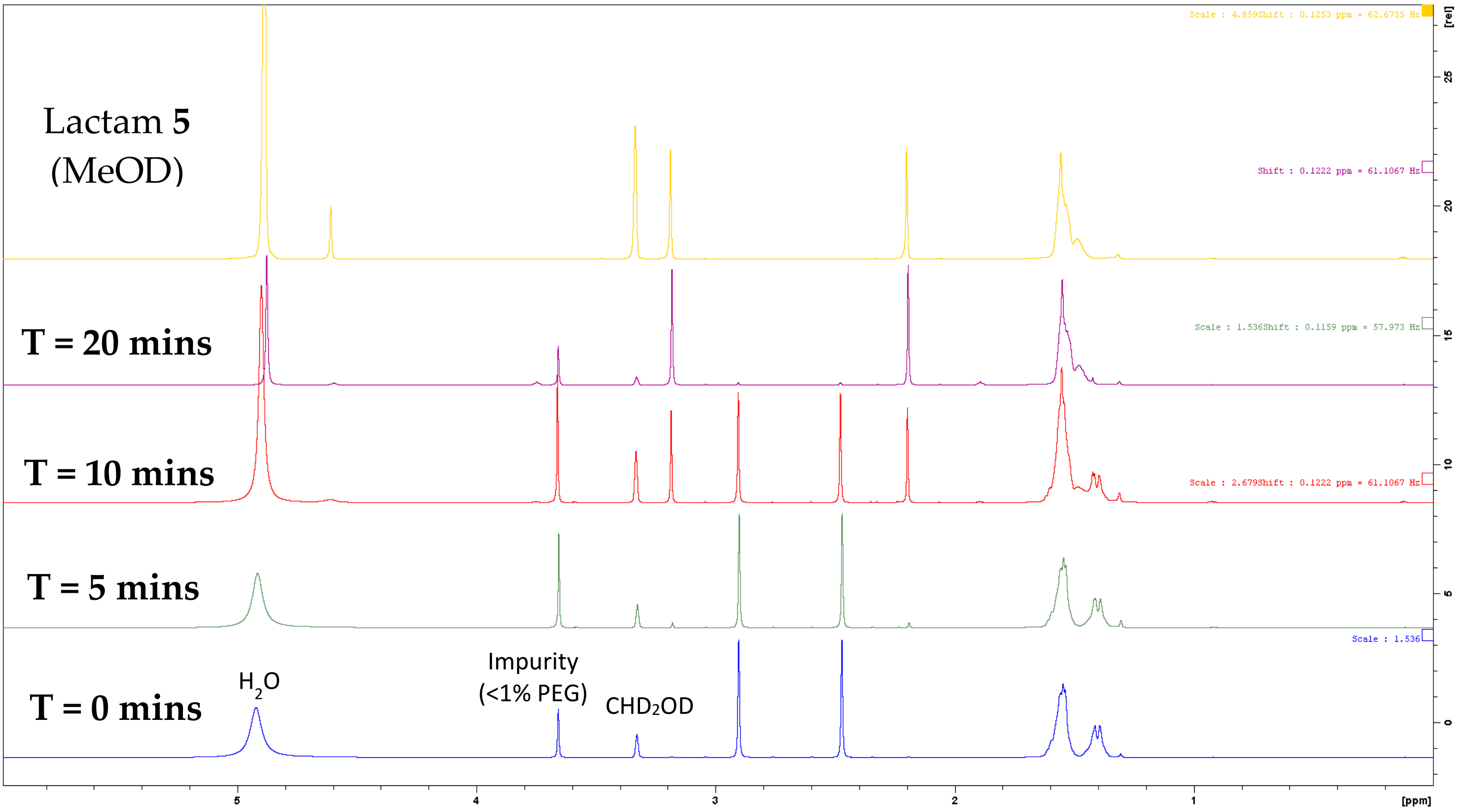

(b) LC-MS conditions

Spectrometer:

UPLC Aquity Hclass Waters with mass spectrometer Vion IMS Qtof Waters

Column:

Acquity UPLC BEH C18 1.7um

2.1 * 50mm

Sample concentration:

1ppm

General conditions:

Quaternary Solvent Manager

General

Solvent Name A: Water + 0.1% Formic Acid

Solvent Name B:

Solvent Name C:

Solvent Name D: Acetonitrile + 0.1% Formic Acid

Comment:

Low Pressure Limit: 0 psi

High Pressure Limit: 15000 psi

Gradient Table

Time (min)	Flow Rate (mL/min)	Compositio n A (%)	Compositio n B (%)	Compositio n C (%)	Compositio n D (%)	Curve
0.00	0.400	90.0	0.0	0.0	10.0	Initial
0.50	0.400	90.0	0.0	0.0	10.0	6
3.00	0.400	80.0	0.0	0.0	20.0	6
5.00	0.400	80.0	0.0	0.0	20.0	6
5.01	0.400	90.0	0.0	0.0	10.0	6
6.00	0.400	90.0	0.0	0.0	10.0	6

Ionization conditions:

Function 1 : MS

Mode: MS

Start time: 0.00 min

End time: 6.00 min

Low mass: 50 m/z

High mass: 800 m/z

Scan time: 0.150 s

Collision energy mode: Specific

Collision energy: 3 V

Enable Intelligent Data Capture: Yes

Function 2 : MRM

Mode: MRM

Start time: 0.00 min

End time: 6.00 min

Transitions

Precursor (m/z)	Precursor resolution	Precursor charge state	Product (m/z)	Collision energy (V)	Scan time (s)	Spectrum
172.13	Low	1	172.13	3	Automatic	Narrow

Function 3 : MRM

Mode: MRM

Start time: 0.00 min

End time: 6.00 min

Transitions

Precursor (m/z)	Precursor resolution	Precursor charge state	Product (m/z)	Collision energy (V)	Scan time (s)	Spectrum
154.12	Low	1	154.12	3	Automatic	Narrow

Source parameters

Source type: ESI

Source temperature: 100 °C

Desolvation temperature: 250 °C

Cone gas: 50 L/h

Desolvation gas: 1000 L/h

Capillary: 0.70 kV

Sample cone voltage: 20 V

Source offset voltage: 40 V

(c) LC-MS total ion count monitoring

T = 0 mins

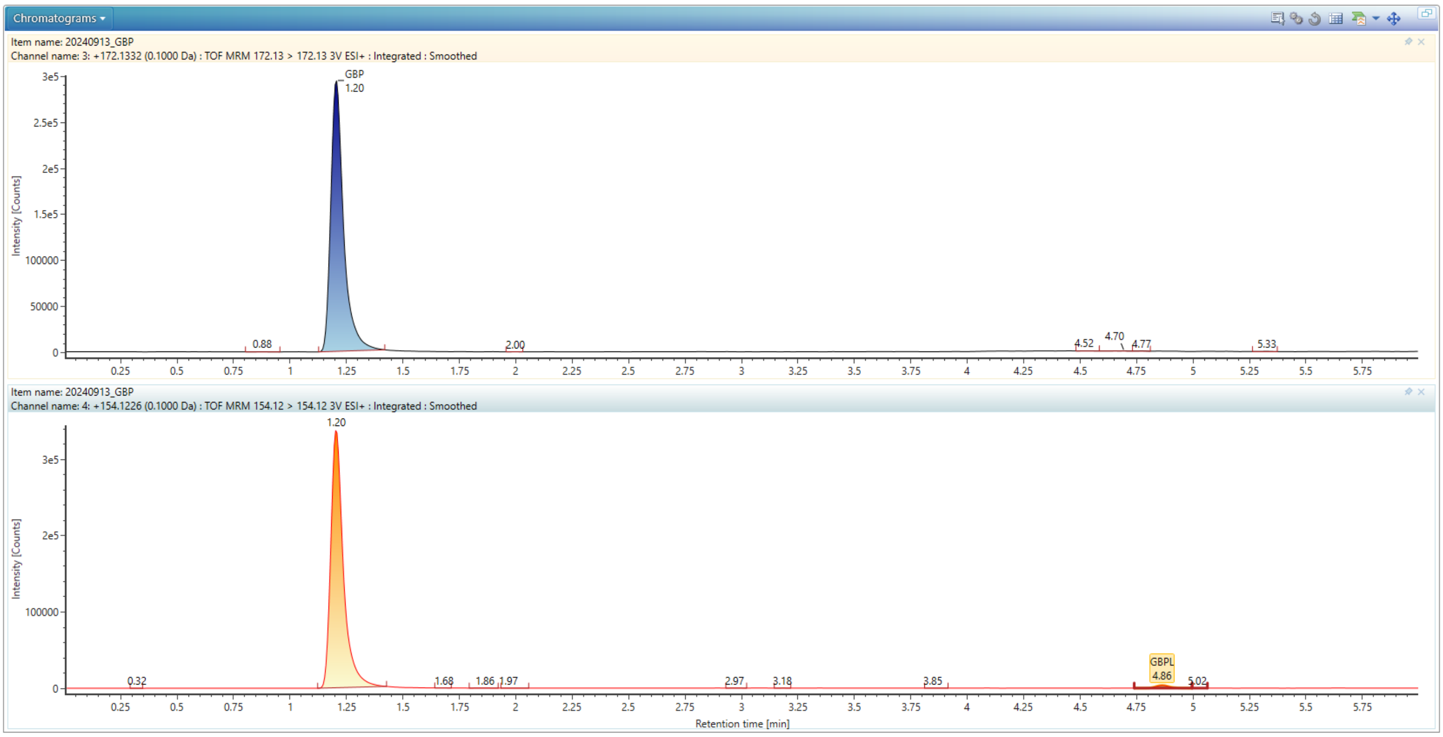

T = 10 mins

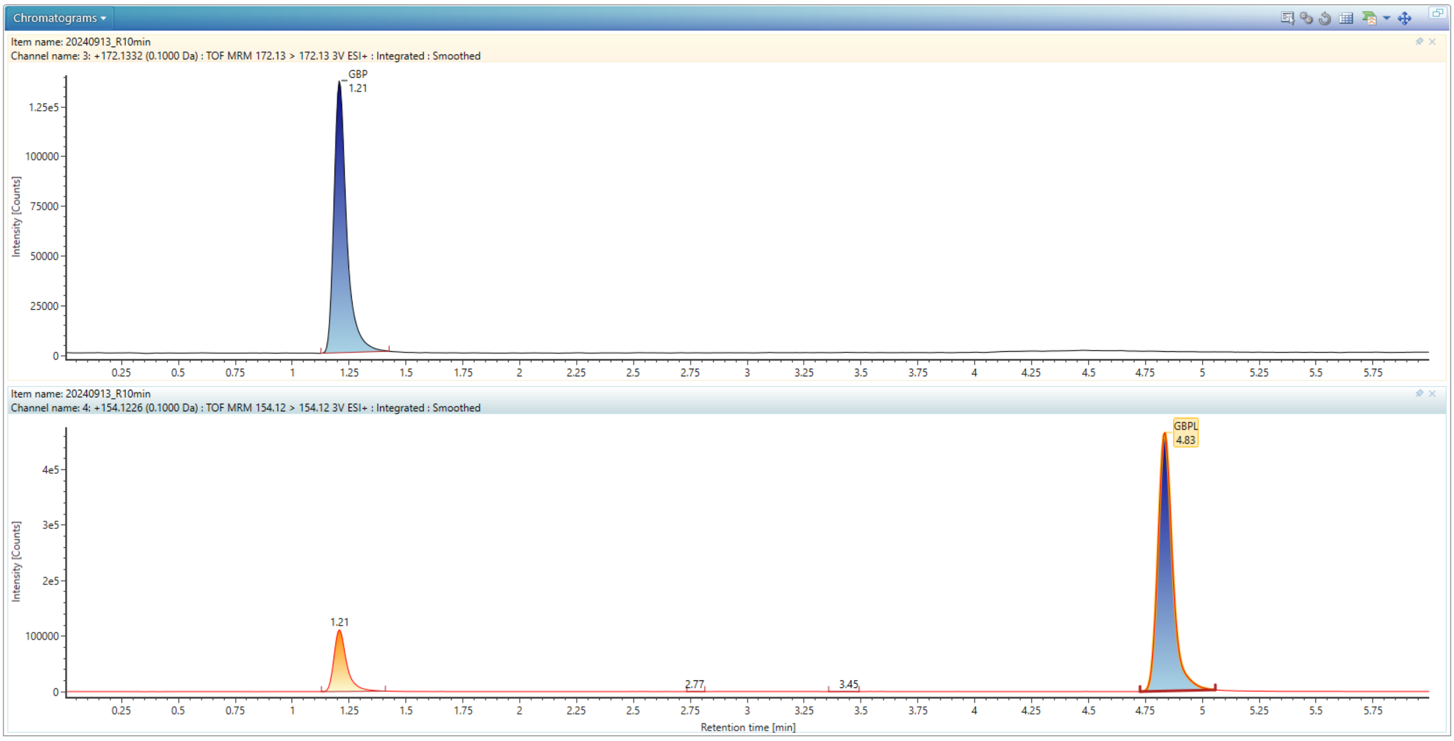

T = 20 mins

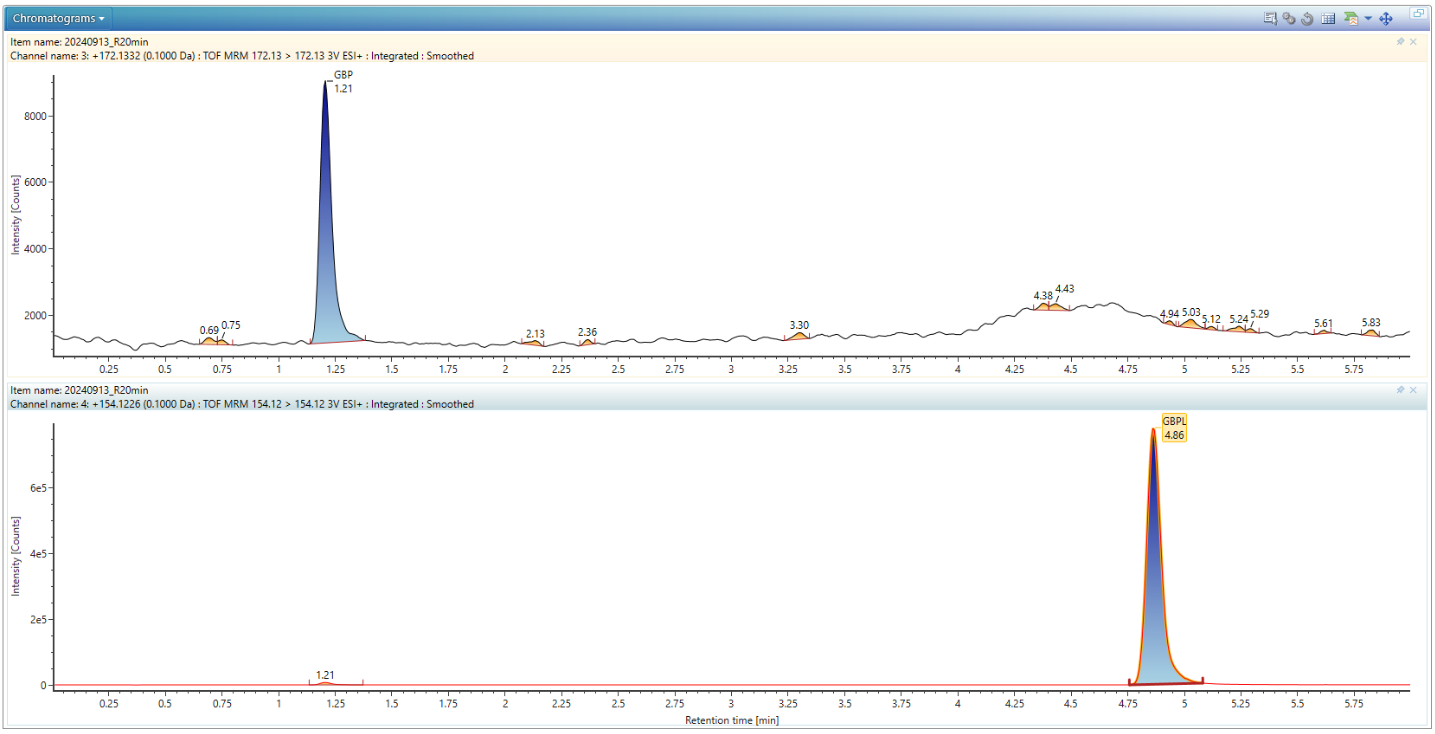
Chromatograms
Item name: 20240913_R20min
Channel name: 3: +172.1332 (0.1000 Da) : TOF MRM 172.13 > 172.13 3V ESI+ : Integrated : Smoothed
GBP
1.21
Intensity [Counts]
Item name: 20240913_R20min
Channel name: 4: +154.1226 (0.1000 Da) : TOF MRM 154.12 > 154.12 3V ESI+ : Integrated : Smoothed
GBPL
4.86
Retention time [min]

(d) Photos of the lactamization reaction of **1** to **5**:

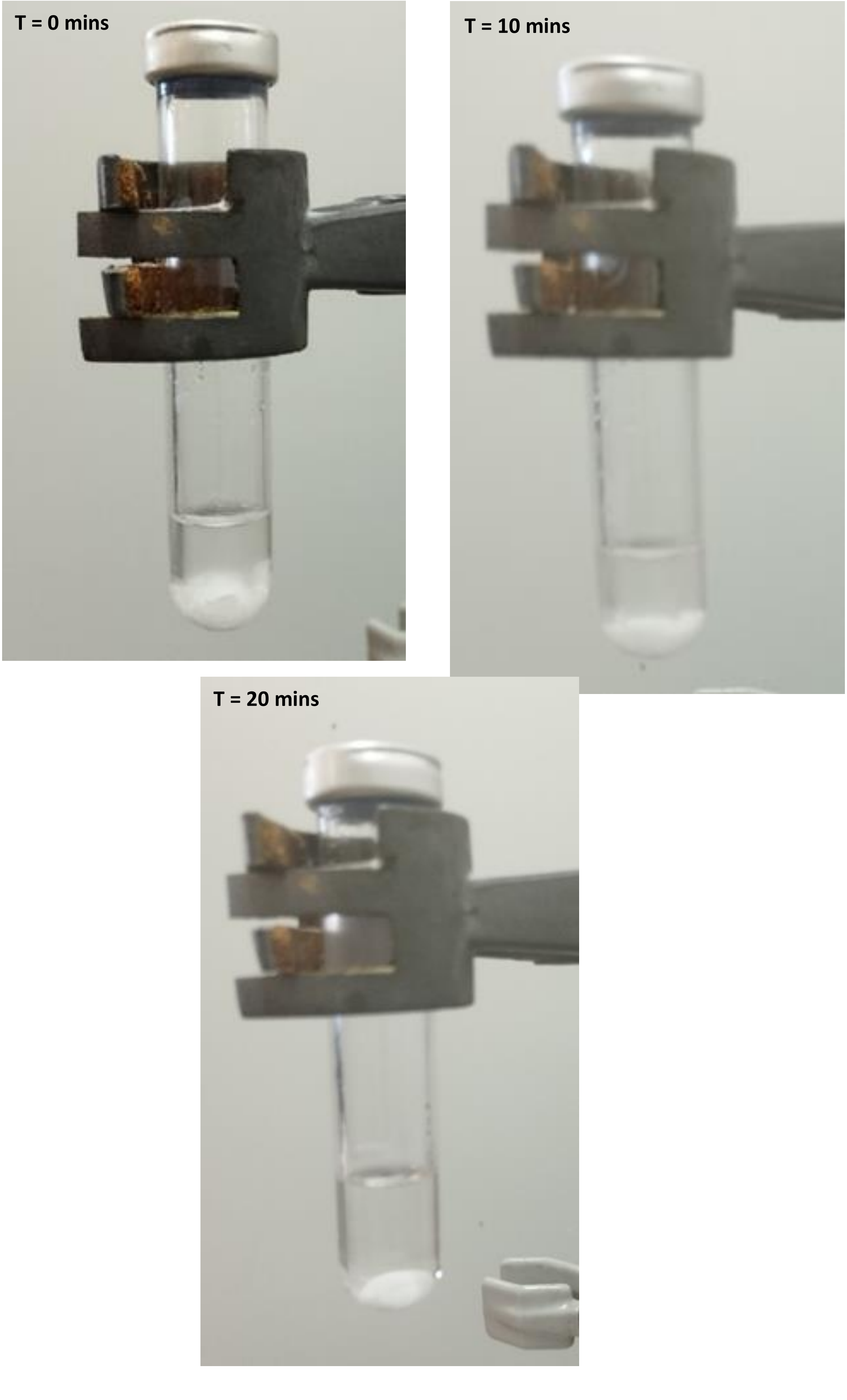

^{1}H NMR (CD_3OD) of GABA lactamization reaction in DMF:

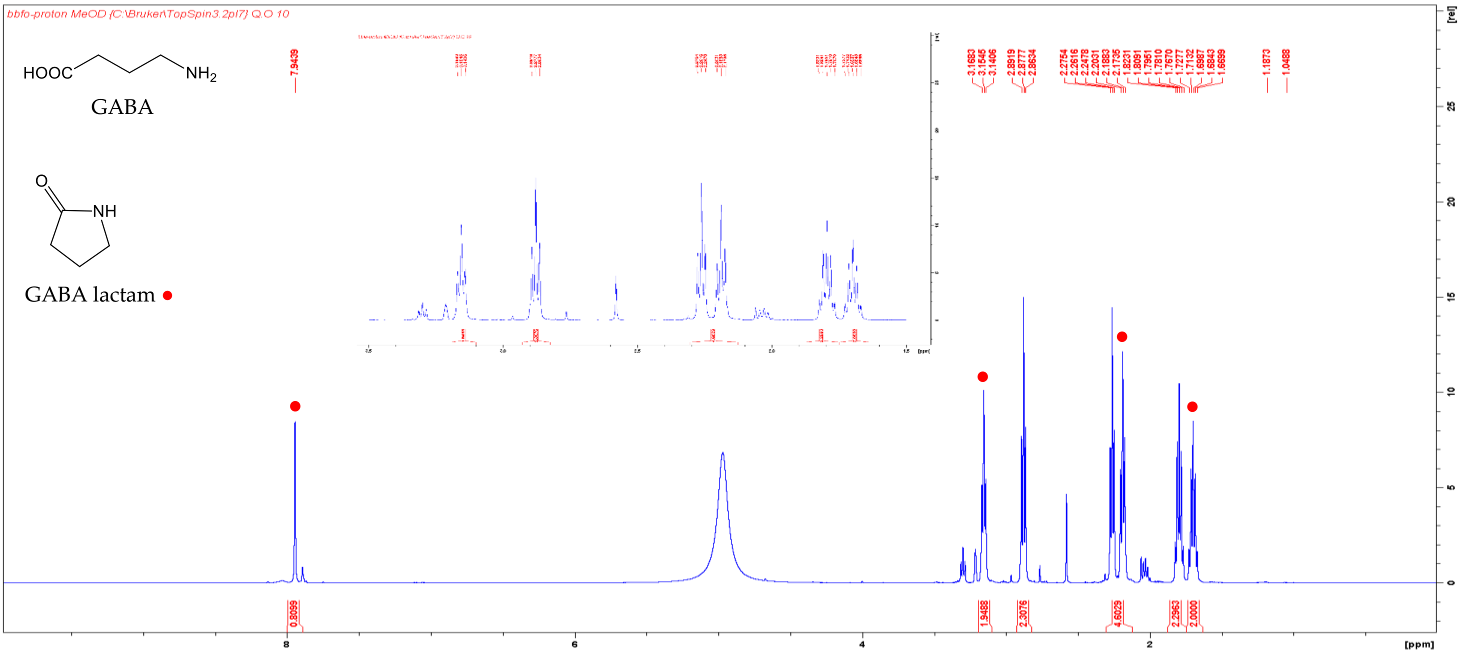

Supplement: Supplementary file 1 [file molecules-29-04811-s001.zip › molecules-3156957-supplementary.pdf]
